# Associations of socioeconomic status with infectious diseases mediated by lifestyle, environmental pollution and chronic comorbidities: a comprehensive evaluation based on UK Biobank

**DOI:** 10.1186/s40249-023-01056-5

**Published:** 2023-01-30

**Authors:** Xiangyu Ye, Yidi Wang, Yixin Zou, Junlan Tu, Weiming Tang, Rongbin Yu, Sheng Yang, Peng Huang

**Affiliations:** 1 Department of Epidemiology, Center for Global Health, School of Public Health, Nanjing Medical University, Nanjing, China; 2 Institute of Global Health and Infectious Diseases, University of North Carolina, Chapel Hill, CA USA; 3 Department of Biostatistics, Center for Global Health, School of Public Health, Nanjing Medical University, Nanjing, China

**Keywords:** Socioeconomic status, Infectious diseases, Healthy lifestyle, Environmental pollution, Chronic comorbidities

## Abstract

**Background:**

Socioeconomic status (SES) inequity was recognized as a driver of some certain infectious diseases. However, few studies evaluated the association between SES and the burden of overall infections, and even fewer identified preventable mediators. This study aimed to assess the association between SES and overall infectious diseases burden, and the potential roles of factors including lifestyle, environmental pollution, chronic disease history.

**Methods:**

We included 401,009 participants from the UK Biobank (UKB) and defined the infection status for each participant according to their diagnosis records. Latent class analysis (LCA) was used to define SES for each participant. We further defined healthy lifestyle score, environment pollution score (EPS) and four types of chronic comorbidities. We used multivariate logistic regression to test the associations between the four above covariates and infectious diseases. Then, we performed the mediation and interaction analysis to explain the relationships between SES and other variables on infectious diseases. Finally, we employed seven types of sensitivity analyses, including considering the Townsend deprivation index as an area level SES variable, repeating our main analysis for some individual or composite factors and in some subgroups, as well as in an external data from the US National Health and Nutrition Examination Survey, to verify the main results.

**Results:**

In UKB, 60,771 (15.2%) participants were diagnosed with infectious diseases during follow-up. Lower SES [odds ratio (*OR*) = 1.5570] were associated with higher risk of overall infections. Lifestyle score mediated 2.9% of effects from SES, which ranged from 2.9 to 4.0% in different infection subtypes, while cardiovascular disease (CVD) mediated a proportion of 6.2% with a range from 2.1 to 6.8%. In addition, SES showed significant negative interaction with lifestyle score (*OR* = 0.8650) and a history of cancer (*OR* = 0.9096), while a significant synergy interaction was observed between SES and EPS (*OR* = 1.0024). In subgroup analysis, we found that males and African (AFR) with lower SES showed much higher infection risk. Results from sensitivity and validation analyses showed relative consistent with the main analysis.

**Conclusions:**

Low SES is shown to be an important risk factor for infectious disease, part of which may be mediated by poor lifestyle and chronic comorbidities. Efforts to enhance health education and improve the quality of living environment may help reduce burden of infectious disease, especially for people with low SES.

**Supplementary Information:**

The online version contains supplementary material available at 10.1186/s40249-023-01056-5.

## Background

The rapid development of socioeconomic have been improving the life quality, but also exacerbated the wealth inequity within countries, regions and groups [1, 2]. Socioeconomic status (SES) inequity is still associated with disease and health-related outcomes [3]. For example, heavier burden of cardiovascular diseases (CAD), cancer, and physical disorders have been reported in groups with low SES [4–6]. The situation is always worse when it comes to ethnic differences, which are usually viewed as an important source of SES inequity [7–10]. In spite of the notable achievements in the work of infection prevention and control, have made the public pay more attention to chronic non-infectious diseases, epidemics and intermittent outbreaks of infections continue to arouse regional and international concerns [11–13]. Infectious diseases remain a main contributor to morbidity and mortality, especially in low- and middle-income countries and regions [14, 15]. It is still of great necessity and meaning to make efforts to reduce socioeconomic inequity and further reduce the burden of infectious diseases.

SES represents a composite measurement of an individual’s economic and sociological standing and is usually assessed from the perspectives of educational attainment, income, and occupation [16, 17]. Apart from determining the quality and accessibility of health care directly [18], SES profoundly impacts an individual's lifestyle and a regional environment [19, 20], both of which also have been reported as important drivers of morbidity and mortality [21, 22]. Recently, researchers are showing a growing interest in the joint influence of multiple lifestyle behaviors or ambient air pollutants on health-related outcomes, and have re-emphasized the importance of maintaining a healthy lifestyle and protecting the environment from a comprehensive perspective [23, 24]. Multiple lifestyle and environmental pollutants have also been demonstrated to be associated with the occurrence and progression of infectious diseases, mainly via distorting the immune system or affecting an individual’s chance of exposure to some pathogens [25–27]. However, several limitations existed in previous studies. First, most studies defined these exposures from a single perspective, which made it difficult to reflect these variables comprehensively. Second, existing studies were usually carried out on some specific infections, and thus hardly identify risk factors that contribute to the increased burden of overall infectious diseases from a holistic perspective. Third, it also remains unclear whether these associations identified varied across different sex and ethnic subgroups, which is important for the development of monitoring and management policies. Last but not least, even fewer studies have shown the association between infection and SES.

Here, we used prospective cohort data from the UK Biobank (UKB) to assess the associations between SES, as well as lifestyle, environmental pollution and several chronic comorbidity factors, and infectious diseases. We further explored the potential roles of lifestyle, environmental pollution, and these chronic comorbidities in the association between SES and infectious diseases. Finally, we conducted a series of subgroup analyses to evaluate these associations across sex and ethnic subpopulations. In addition, we also used data from the US National Health and Nutrition Examination Survey (US NHANES) to validate our findings.

## Methods

### Study population

**Fig. 1:**
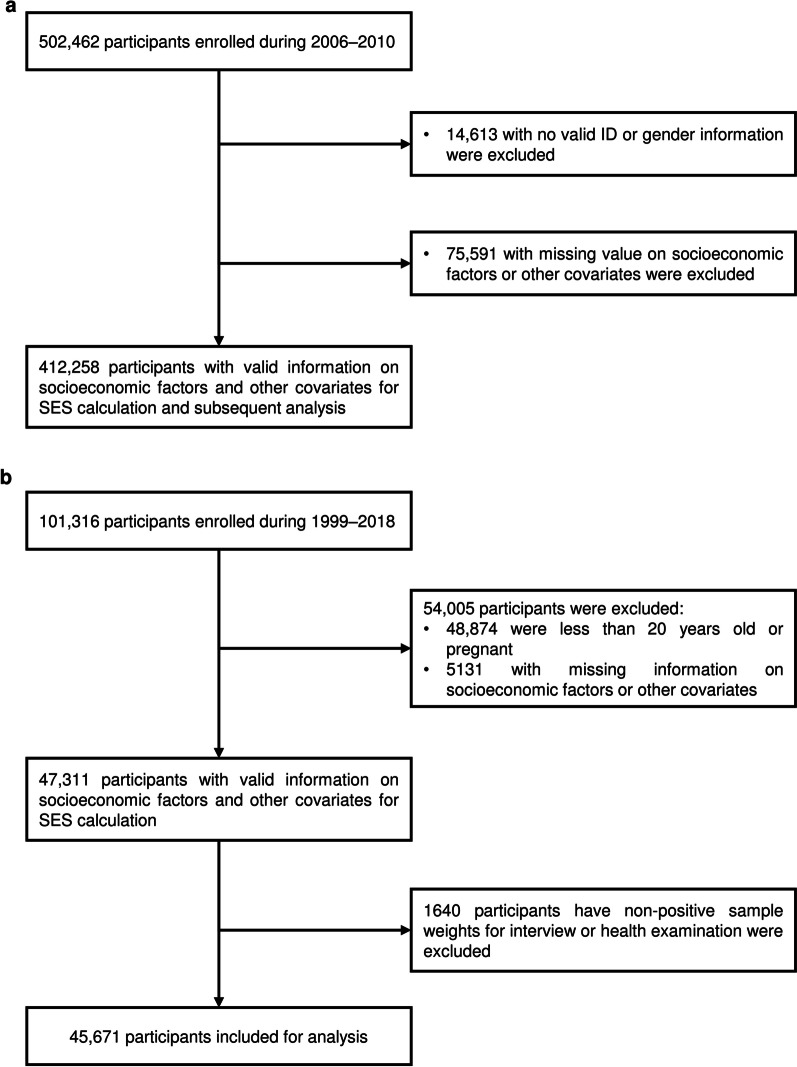
Flowchart of the participants selection in the UK Biobank (**a**) and US NHANES (**b**). *SES* socioeconomic status

UKB is a repository of research data sourced from ~ 500,000 UK-wide participants aged around 40–70 years old, recruited from 22 assessment centers during 2006–2010 [28]. We used data collected for each participant from enrollment to March 26, 2021. In brief, data in the UKB repository was grouped into 277 categories, and we retrieved those related to (i) socioeconomic factors (categories 100,066, 100,063, and 100,064); (ii) lifestyle factors (categories 100,058, 100,054, 100,052, 100,051, 100,057, and 143); (iii) environmental pollution factors (categories 114 and 115); (iv) health outcome factors (categories 2002, 100,074, 100,060, 137, and 100,092) (Additional file 1: Table S1) [29]. Note that although an individual's SES and lifestyle may change over time, we used the baseline survey data to define the socioeconomic and lifestyle status of each participant. A research protocol for our study has obtained all necessary approvals from the UKB’s review committees. We accessed to the UKB cohort consisting of 502,462 individuals. Following Yang and Zhou [30, 31], we removed individuals: (i) who have sex mismatched; (ii) who are redacted and thus do not have a corresponding ID; (iii) who have missing information on socioeconomic factors or other covariates. Finally, we retained 412,258 participants in UKB for subsequent analysis (Fig. 1a).

In US NHANES, we included 101,316 participants surveyed from 1999 to 2018, and followed Zhang et al. to remove individuals: (i) who were less than 20 years old; (ii) who were pregnant; (iii) who had missing information on socioeconomic factors or other covariates; (iv) who had non-positive sample weights for an interview or health examination in the datasets [32]. Finally, we retained 45,671 participants in US NHANES for subsequent analysis (Fig. 1b). Details about the introduction, the definitions of socioeconomic, lifestyle, and chronic comorbidity factors, and infectious diseases in US NHANES are provided in Additional file 1: Tables S2 and S3, and Additional file 2: Methods.

### Assessment of socioeconomic status

We followed Zhang et al. to assess the individual SES based on four variables collected at baseline, including family income level, education qualification, employment status, and health insurance coverage [32]. In particular, however, considering the implementation of the National Health Service, a publicly funded healthcare system in the UK, we used three variables, including the total household income level, education qualification and employment status, rather than the health insurance coverage, to assess the SES of each participant at individual level [33]. For total household income level before tax, participants chose an option from (i) < £18,000; (ii) £18,000–£30,999; (iii) £31,000–£51,999; (iv) £52,000–£100,000; (v) > £100,000; (vi) do not know; and (vii) prefer not to answer. We removed the participants choosing the last two options. Education qualification was recorded as (i) College or University degree; (ii) A levels, AS levels, or equivalent; (iii) O levels, GCSEs, or equivalent; (iv) CSEs or equivalent; (v) NVQ, HND, HNC, or equivalent; (vi) other professional qualifications; and (vii) none of the above (following Zhang et al. [32] we treated it as equivalent to or less than high school diploma); and (viii) prefer not to answer. We removed the individuals choosing the last option. Considering no clear rank order of employment status among candidate options, including (i) in paid employment or self-employed; (ii) retired; (iii) looking after home and/or family; (iv) unable to work because of sickness or disability; (v) unemployed; (vi) doing unpaid or voluntary work; (vii) full or part-time student; (viii) none of the above; and (ix) prefer not to answer, we removed participants choosing the last option and simply regrouped the remaining participants into two groups: employed (those chose (i), (ii), (vi) and (vii)) and unemployed (those chose others). Variable definitions were listed in Additional file 1: Table S1.

Following Zhang et al. [32] we then used latent class analysis (LCA), using multiple observed categorical variables to construct an unmeasured variable (i.e., latent variable), to estimate SES based on the above three variables in UKB. We used R package *poLCA* (v1.6.0) to implement the LCA procedure, and set the maximum times of iterations to 10,000, and the tolerance value for judging convergence to 1 × 10^–6^ [34]. To select a reasonable latent class number, we fitted the different LCA model with 2–10 latent classes. Models failed to converge when the class number is greater than five. We further used Akaike information criterion (AIC), Bayesian information criterion (BIC), and likelihood ratio statistic (*G*^*2*^) for parameter selection, and treated latent class with mean posterior probability higher than 0.7 as classification with acceptable uncertainty (Additional file 1: Table S3 and Additional file 2: Fig. S1). Finally, three latent classes were identified, which respectively represented a high, medium, and low SES according to the item-response probabilities (Additional file 1: Table S3).

In addition, for UKB, we also included the Townsend deprivation index (TDI) as an area level SES, which represents a comprehensive score of four key variables: unemployment, overcrowded household, non-car ownership, and non-home ownership, with a higher score representing higher levels of deprivation [35, 36].

### Assessment of lifestyle factors

Following Said et al., Fan et al., and Zhu et al. [37–39] we included information on five healthy lifestyle factors collected at baseline, including “no current smoking”, “regular physical activity”, “healthy diet pattern”, “no alcohol consumption”, and “healthy sleep pattern”. In addition, given that drug abuse behavior has been proved a high-risk factor for some infectious diseases [40, 41], we also regarded “no drug use” as the sixth healthy lifestyle factor. We then used the six healthy lifestyle factors to generate a comprehensive lifestyle score.

Lifestyle information in UKB was also obtained through structured questionnaires (Additional file 1: Table S1). “No current smoking” was defined as never smoking or former smoking but had quit for more than 30 years. “No alcohol consumption” was defined as never drinking alcohol. UKB records the use of cannabis, and “No drug use” was defined as never use cannabis. “Regular physical activity” was defined to meet one of the following: (i) from the perspective of frequency, to engage in vigorous physical activity for at least one day and moderate activity for at least five days per week; (ii) from the perspective of time, to exercise of vigorous activity for at least 75 min or moderate activity for 150 min per week. “Healthy diet pattern” includes (i) adequate consumption of fruit, (ii) vegetables, (iii) fish, and (iv) whole grains, but (v) reduced consumption of processed and (vi) unprocessed meats. The specific definition for each pattern was in Additional file 1: Table S1, and we defined a healthy diet pattern as following at least four factors. As for sleep patterns, five sleep factors, including chronotype, duration, insomnia, snoring, and involuntary daytime sleepiness, over the last four weeks were considered and surveyed [38]. “Healthy sleep pattern” was defined as: (i) self-reported as early chronotype; (ii) sleep 7–8 h per day; (iii) rarely suffer from insomnia; (iv) no snoring symptoms; and (v) infrequently doze off or fall asleep involuntarily during the daytime. The specific definition for each pattern was also in Additional file 1: Table S1, and we defined a healthy sleep pattern as following at least four of these five factors.

For each lifestyle factor, we assigned 1 point for a healthy level while 0 points for an unhealthy level. The lifestyle variable was defined as the summation of the six variables and was divided participants into 3 groups: poor group (0–1 point), medium (2–3 points) and healthy (4–6 points).

### Assessment of environmental pollution

Environmental pollution information was recorded only in UKB. Following Huang et al. and Furlong et al. [42, 43] we considered eight environmental pollution factors, including particulate matter ≤ 2.5 μm (PM_2.5_), particulate matter 2.5–10 μm (PM_2.5–10_), particulate matter ≤ 10 μm (PM_10_), nitrogen oxides (NO_x_), and nitrogen dioxide (NO_2_), noise, distance to nearest major road, and traffic intensity (Additional file 1: Table S1). All environmental pollution factors were estimated by the Small Area Health Statistics Unit as part of the BioSHaRE-EU Environmental Determinants of Health Project. Values of PM_2.5_, PM_2.5–10_, PM_10_, NO_x_, NO_2_ and noise were calculated in 2010 using a Land Use Regression (LUR) model developed as part of the European Study of Cohorts for Air Pollution Effects (ESCAPE) and represented annual averages of air pollution in 2010 for the reported residence at enrollment [44, 45]. Specifically, given that impacts of noise usually vary over a time period, a day-evening-night equivalent level with a 5 dB and 10 dB penalty added to the average sound level of noise pollution of the evening (19:00 to 23:00) and night-time (overnight 23:00 to 07:00), respectively. We used weighted average noise exposure level measured over a 24-h period to further analysis [43, 46, 47]. In addition, distance to the nearest major road and traffic intensity were measured based on the local road network from the Ordnance Survey Meridian 2 road network in 2009. We treated the estimated values for 2009 and 2010 as a proxy for a measure of chronic, long-term exposure to environmental pollutants, following previous studies [24, 43]. Note that to facilitate interpretation, we calculated the odds ratio (*OR*) per 10-unit increase in each environmental pollution factor to reflect its association with infection [43]. To demonstrate the reasonability of this proxy, we also conducted a side analysis using participants enrolled in 2010, which is also a part of sensitivity analyses.

We then created weighted environment pollution score (EPS) through adding measurements of eight environmental pollutants, weighted by the adjusted estimates from multivariable analysis on the prevalence of infectious diseases [48]. The equation is as follows:

$$\begin{array}{c}{EPS}_{i} = \frac{p}{\sum {{\varvec{\beta}}}_{j}}{\sum }_{j = 1}^{p}{{\varvec{\beta}}}_{j}{{\varvec{X}}}_{ij}\#\left(1\right)\end{array}$$

where $p$ represented the number of environmental pollutants; ${{\varvec{\beta}}}_{j}$ was adjusted coefficients of environmental pollutants $j$; ${{\varvec{X}}}_{ij}$ and ${EPS}_{i}$ was the measurements of $j$ th pollution of $i$ th individual. We also calculated a weighted air pollution score (APS) using PM_2.5_, PM_2.5–10_, PM_10_, NO_x_, NO_2_, as done in previous studies to serve as a sensitivity analysis. Note that for the analysis on the association of EPS and APS with infection, we divided the participants into five groups (Q1–Q5) according to the quantiles of the scores, and evaluated the association between score groups and infection, as well as *OR*s of groups with higher scores (Q2–Q5) to the group with lowest scores.

### Assessment of chronic comorbidities

We considered four types of chronic comorbidities, including cardiovascular disease (CVD), diabetes, psychiatric disorders and cancer (Additional file 1: Table S1). We followed Zhu et al. and Said et al. [39, 49] and used diagnosis records in UKB coded by International Classification of Diseases version-10 (ICD-10) to define participants with CVD, diabetes and cancer at baseline. Specifically, we totally defined 35,469 (8.8%) participants with CVD history, including 5055 (1.3%) CAD cases (ICD-9 codes 410–412; ICD-10 codes I21–I23, I24.1, and I25.2), 4824 (1.2%) atrial fibrillation (AF) cases (ICD-9 codes 4273; ICD-10 codes I48), 1945 (0.5%) stroke cases (ICD-9 codes 430, 431, 434, and 436; ICD-10 codes I60, I61, I63, and I64), and 29,294 (7.3%) hypertension cases (ICD-9 codes 401–405; ICD-10 codes I10–I13, I15, O10). We also defined 7922 (2.0%) and 30,176 (7.5%) participants with a history of diabetes (ICD-9 codes 250; ICD-10 codes E10–E14) and cancer (ICD-10 codes C00–D48), respectively. In terms of psychiatric disorders, we followed Davis et al. [50] and considered participants who had self-reported anxiety, depression or bipolar disorder. Specifically, we totally defined 58,381 (14.6%) participants with a history of psychiatric disorders, including 23,079 (5.8%), 45,023 (11.2%) and 1582 (0.4%) with anxiety (field 20,002 codes 1287; field 20,544 codes 15), depression (field 20,002 coded 1286; field 20,126 coded 3–5; field 20,544 codes 11) and bipolar disorder (field 20,002 coded 1291; field 20,126 coded 1–2; field 20,544 codes 10), respectively.

### Definition of outcome

In UKB, infectious diseases were also defined according to diagnosis records in UKB coded by the ICD-10 and ICD-9. We used data collected up to March 26, 2021. Referring to the coding terms, we defined a total of 60,771 (14.7%) cases with infectious diseases (ICD-10 codes A00–B99 and J00–J22; ICD-9 codes 001–139 and 480–487). Furthermore, we also defined three subtypes of infectious diseases from it: (i) respiratory infectious diseases (ICD-10 codes A15, A37, A39, B01, B02, B05, B06, B26, and J09–J11; ICD-9 codes 001, 012, 033, 036, 053, 055, 056, 072 and 487) with 2119 (3.5%) cases; (ii) digestive infectious diseases (ICD-10 codes A00–A09, B15, B17.2, B67, B68, B77, B80, and B82; ICD-9 codes 001–009, 0701, and 122) with 15,019 (24.7%) cases; (iii) blood or sexually transmitted infectious diseases (ICD-10 codes A50–A64, B16, B17.1, B18.0, B18.1, B18.2 and B20–B24; ICD-9 codes 0703 and 090–099) with 869 (1.4%) cases, to explore the association of research factors with common infectious diseases types (Additional file 1: Table S1). In addition, we also defined 71,335 participants enrolled in 2010, among whom 9682 (13.6%) were infected, to serve as sensitivity analysis.

### Statistical analysis

Baseline characteristics of three SES groups were compared using the unpaired, 2-tailed *t* test or Mann–Whitney test for continuous variables depending on the data distribution, and the *χ*^2^ test was used for categorical variables. Continuous variables are presented as mean (SD) or median (quartile); categorical variables are presented as number (percentage). Second, multivariable logistic regression was used to test association of SES, lifestyle factors, environmental pollution, and chronic comorbidity factors with infectious diseases. We treated age, sex, ethnicity and assessment center as covariates, and reported adjusted *OR* with 95% confidence intervals (*CIs*). Third, multiplicative interaction analysis, along with stratified analysis, was used to ask about the moderation effects of SES on association of lifestyle, environmental pollution, and chronic comorbidity factors with infectious diseases. A two-sided *P* < 0.05 was considered statistically significant. All analyses were performed using the statistical software R 4.1.0 (Lucent Technologies, Jasmine Mountain, USA).

A mediation analysis was conducted to evaluate the proportion mediated by lifestyle, environmental pollution, and chronic comorbidity factors for the association between SES and infectious diseases. Associations of lifestyle, environmental pollution, and chronic comorbidity factors on infections were tested using logistic regression. Associations of SES on individual lifestyle factors were also analyzed using logistic regression, while those of SES on lifestyle scores, EPS and individual environmental pollutant were analyzed using linear regression. All regression analyses were adjusted for age, sex, ethnic and assessment center.

### Sensitivity analyses

To ensure the robustness of our result, we considered seven kinds of sensitivity analyses. First, in terms of socioeconomic factors, we additionally considered the TDI as an area level SES variable. We not only directly explored its association with infectious diseases, but also took it as a covariate in the association analysis of individual-level SES on infection. Second, in terms of lifestyle, environmental pollution and chronic comorbidities, we repeated all main analyses conducted in those composite variables for each individual factor. Third, in terms of environmental pollution, we further calculated a weighted APS using five air pollution factors, including PM_2.5_, PM_2.5–10_, PM_10_, NO_x_, NO_2_, as done in previous studies [24, 48]. Fourth, in terms of infectious diseases, considering that we took environmental pollutants measurement in 2009 and 2010 as a proxy for chronic, long-term exposure estimation, we also repeated the main analysis in a subset of participants enrolled in 2010. Fifth, given the case–control imbalance in analysis of different infectious diseases subgroups, we performed a propensity score matching (PSM). We treated age, sex, ethnicity and assessment center as matching covariates, and used the nearest neighbor method to make a 1:4 matching. Finally, we additionally used data from US NHANES to validate our main results. We repeated the main analysis in US NHANES, except for those on environmental pollution variables. In particular, due to the application of oversampling in US NHANES survey, we considered sample weights recorded in US NHANES, which indicate a measure of the number of people in the population represented by a specific person, in descriptive and other analysis to obtain accurate point estimates and standard errors. Note that frequency was reported directly based on the sample data (i.e., the 47,311 sampled participants), while other statistics were estimated and reported in a weighted manner. *Survey* (v 4.1.1) and *svrepmisc* (v 0.2.2) packages were used to account for the sample weights. Covariates used for US NHANES included age, sex, ethnicity and survey cycle.

## Results

### Population characteristics

We totally included 412,258 participants from UKB participants (Fig. 1a). All participants were enrolled during 2006–2010, and data for each participant was collected from enrollment to March 26, 2021. The variable definitions are described in the Methods section (Additional file 1: Table S1–S3 and Additional file 2: Fig. S1). Table 1 shows the baseline characteristics. The participants have a mean age of 56.16 ± 8.08 years, among whom 215,933 (52.4%) were women, 80,949 (19.6%) were of high SES, 215,967 (52.4%) of medium SES, and 115,342 (28.0%) of low SES.

**Table 1:** Baseline characteristics and infection status of all participants from UK biobank

Variable	All (*N* = 412,258)	High SES (*N*_1_ = 79,407)	Medium SES (*N*_2_ = 210,475)	Low SES (*N*_3_ = 111,127)	*P*
White ethnicity or race^a^	392,279 (95.2)	77,577 (95.8)	206,527 (95.6)	108,175 (93.8)	< 0.0001
TDI^b^	− 2.1744 (− 3.6664, 0.4384)	− 2.7174 (− 4.011, − 0.5936)	− 2.4366 (− 3.7872, − 0.2287)	− 0.9134 (− 2.9558, 2.2916)	< 0.0001
Female^a^	215,933 (52.4)	39,320 (48.6)	111,935 (51.8)	64,678 (56.1)	< 0.0001
Age^b^	56.1625 ± 8.0839	52.5195 ± 7.2159	55.7701 ± 7.9982	59.4541 ± 7.5375	< 0.0001
BMI^b^	27.3836 ± 4.7556	26.4616 ± 4.2599	27.3404 ± 4.6486	28.1116 ± 5.1484	< 0.0001
BMI group^a^					
≤ 18.5	2055 (0.5)	388 (0.5)	936 (0.4)	731 (0.6)	< 0.0001
18.51–29.9	311,295 (75.5)	66,480 (82.1)	164,607 (76.2)	80,208 (69.5)	
≥ 30	98,908 (24.0)	14,081 (17.4)	50,424 (23.3)	34,403 (29.8)	
SES ^a^					
High	80,949 (19.6)	80,949 (100)	0 (0)	0 (0)	< 0.0001
Medium	215,967 (52.4)	0 (0)	215,967 (100)	0 (0)	
Low	115,342 (28.0)	0 (0)	0 (0)	115,342 (100)	
Income (£)^a^					
> 100,000	22,422 (5.4)	21,977 (27.1)	0 (0)	445 (0.4)	< 0.0001
52,000–100,000	84,213 (20.4)	57,688 (71.3)	26,525 (12.3)	0 (0)	
31,000–51,999	107,833 (26.2)	1284 (1.6)	106,067 (49.1)	482 (0.4)	
18,000–30,999	104,914 (25.4)	0 (0)	83,375 (38.6)	21,539 (18.7)	
< 18,000	92,876 (22.5)	0 (0)	0 (0)	92,876 (80.5)	
Education^a^					
College or University degree	144,681 (35.1)	63,515 (78.5)	65,794 (30.5)	15,372 (13.3)	< 0.0001
A levels/AS levels or equivalent	48,049 (11.7)	13,909 (17.2)	25,961 (12.0)	8179 (7.1)	
O levels/GCSEs or equivalent	87,724 (21.3)	2024 (2.5)	64,330 (29.8)	21,370 (18.5)	
CSEs or equivalent	22,284 (5.4)	250 (0.3)	15,863 (7.3)	6171 (5.4)	
NVQ or HND or HNC or equivalent	27,310 (6.6)	490 (0.6)	18,833 (8.7)	7987 (6.9)	
Other professional qualifications	20,944 (5.1)	761 (0.9)	15,190 (7.0)	4993 (4.3)	
None of the above	61,266 (14.9)	0 (0)	9996 (4.6)	51,270 (44.5)	
Unemployed^a^	30,261 (7.3)	4850 (6.0)	3407 (1.6)	22,004 (19.1)	< 0.0001
Lifestyle scores^a^					
0–1	77,022 (26.5)	15,471 (24.8)	38,605 (25.0)	22,946 (31.1)	< 0.0001
2–3	159,055 (54.8)	36,400 (58.3)	86,751 (56.2)	35,904 (48.7)	
4–6	54,341 (18.7)	10,613 (17.0)	28,896 (18.7)	14,832 (20.1)	
Lifestyle factors					
No current smoking^a^	243,416 (59.2)	52,813 (65.3)	130,188 (60.4)	60,415 (52.6)	< 0.0001
Regular physical activity^a^	95,450 (23.5)	15,571 (19.3)	51,377 (24.0)	28,502 (25.5)	< 0.0001
Healthy diet^a^	96,570 (23.6)	19,229 (23.8)	50,883 (23.7)	26,458 (23.3)	0.006
No alcohol consumption^a^	15,550 (3.8)	1476 (1.8)	6508 (3.0)	7566 (6.6)	< 0.0001
Healthy sleep pattern^a^	136,438 (35.5)	32,085 (41.9)	72,595 (36.0)	31,758 (29.9)	< 0.0001
Never use cannabis^a^	106,210 (76.8)	26,058 (67.6)	60,802 (79.6)	19,350 (83.2)	< 0.0001
APS^b^	64.1294 (58.4819, 69.7389)	63.2826 (56.7590, 69.6608)	63.6288 (58.1162, 69.0290)	65.4867 (60.2623, 71.0704)	< 0.0001
EPS^b^	116.3913 (106.8239, 126.3875)	115.0965 (104.0680, 126.3135)	115.5374 (106.1875, 125.1584)	118.6827 (109.7671, 128.6168)	< 0.0001
Environmental pollutants					
PM_2.5_^b^	9.9200 (9.2800, 10.5600)	9.8000 (9.0800, 10.4800)	9.8800 (9.2400, 10.4800)	10.0900 (9.4800, 10.7300)	< 0.0001
PM_2.5–10_^b^	6.1000 (5.8400, 6.6300)	6.1100 (5.8300, 6.6500)	6.0900 (5.8300, 6.5900)	6.1300 (5.8600, 6.6600)	< 0.0001
PM_10_^b^	16.0200 (15.2300, 16.9900)	16.0000 (15.0700, 17.0400)	15.9800 (15.1800, 16.9200)	16.1000 (15.4200, 17.0900)	< 0.0001
NO_2_^b^	26.1000 (21.3600, 31.1400)	25.8500 (20.2900, 31.5800)	25.6000 (21.0500, 30.5100)	27.1500 (22.6800, 31.9300)	< 0.0001
NO_X_^b^	42.2600 (34.2300, 50.6300)	41.0500 (32.0600, 50.4300)	41.5300 (33.7300, 49.5600)	44.2200 (36.7300, 52.6700)	< 0.0001
Noise^b^	55.0900 (53.6600, 57.1533)	55.100 (53.6800, 57.2300)	55.0433 (53.6200, 57.0800)	55.1700 (53.7300, 57.2533)	< 0.0001
Traffic intensity^b^	17,044 (12,581, 25,195)	17,005 (12,591, 24,107)	16,989 (12,378, 25,182)	17,314 (12,802, 25,598)	< 0.0001
Distance to road^b^	378.7879 (167.7852, 751.8797)	374.5318 (165.8375, 757.5758)	390.6250 (172.7116, 775.1938)	362.3188 (159.7444, 714.2857)	< 0.0001
Chronic comorbidities					
CVD^a^	37,144 (9.0)	3312 (4.1)	16,717 (7.7)	17,115 (14.8)	< 0.0001
CAD^a^	5308 (1.3)	430 (0.5)	2237 (1.0)	2641 (2.3)	< 0.0001
AF^a^	5080 (1.2)	578 (0.7)	2363 (1.1)	2139 (1.9)	< 0.0001
Stroke^a^	2034 (0.5)	225 (0.3)	796 (0.4)	1013 (0.9)	< 0.0001
Hypertension^a^	30,701 (7.4)	2522 (3.1)	13,694 (6.3)	14,485 (12.6)	< 0.0001
Diabetes^a^	8379 (2.0)	600 (0.7)	3404 (1.6)	4375 (3.8)	< 0.0001
Psychiatric disorders^a^	60,105 (14.6)	11,349 (14.0)	30,778 (14.3)	17,978 (15.6)	< 0.0001
Anxiety^a^	23,690 (5.7)	4923 (6.1)	12,753 (5.9)	6014 (5.2)	< 0.0001
Depression^a^	46,419 (11.3)	8422 (10.4)	23,451 (10.9)	14,546 (12.6)	< 0.0001
Bipolar disorder^a^	1635 (0.4)	261 (0.3)	653 (0.3)	721 (0.6)	< 0.0001
Cancer^a^	31,322 (7.6)	4619 (5.7)	15,975 (7.4)	10,728 (9.3)	< 0.0001
Infectious diseases^a^	60,771 (14.7)	7276 (9.0)	28,383 (13.1)	25,112 (21.8)	< 0.0001
Respiratory infectious diseases^a^	2119 (0.5)	247 (0.3)	933 (0.4)	939 (0.8)	< 0.0001
Digestive infectious diseases^a^	15,019 (3.5)	1806 (2.2)	6933 (3.1)	6280 (5.2)	< 0.0001
Blood or sexually transmitted infectious diseases^a^	869 (0.2)	106 (0.1)	349 (0.2)	414 (0.4)	< 0.0001

*TDI* Townsend deprivation, *BMI* body mass index, *SES* socioeconomic status, *APS* air pollution score, *EPS* environment pollution score, *PM*_*2.5*_ particulate matter ≤ 2.5 μm, *PM*_*2.5-10*_ particulate matter 2.5–10 μm, *PM*_*10*_ particulate matter ≤ 10 μm, *NOx* nitrogen oxides, *NO*_*2*_ nitrogen dioxide, *CVD* cardiovascular disease, *CAD* cardiovascular diseases, *AF* atrial fibrillation

^a^Categorical variables are presented as number (percentage)

^b^Continuous variables are presented as mean ± standard deviation or median (quartile)

Participants with low SES were more likely to be women, non-white people, and reasonably with higher TDI (all *P* < 0.0001). Low SES tended to be associated with heavy environmental pollution, and several lifestyle factors, such as higher smoking rate, cannabis use rate, less healthy sleep, but more regular physical activity and lower alcohol consumption (all *P* < 0.0001). Participants with low SES also had higher rates of some chronic comorbidities, including CVD, diabetes, psychiatric disorders and cancer with the only exception of anxiety, which had a higher prevalence in the high SES (all *P* < 0.0001) (Table 1).

In addition, socioeconomic factors, lifestyle factors, air pollution factors, and chronic comorbidity factors all showed high inner correlations (all *P* < 0.0001) (Additional file 2: Fig. S2). Notably, TDI showed a high correlation with air pollution factors (all *P* < 0.0001) (Additional file 2: Fig. S2), which suggested a close relationship between area economic and environmental conditions.

Associations of SES, lifestyle, environmental pollution and chronic comorbidity factors with infectious diseases.

**Fig. 2:**
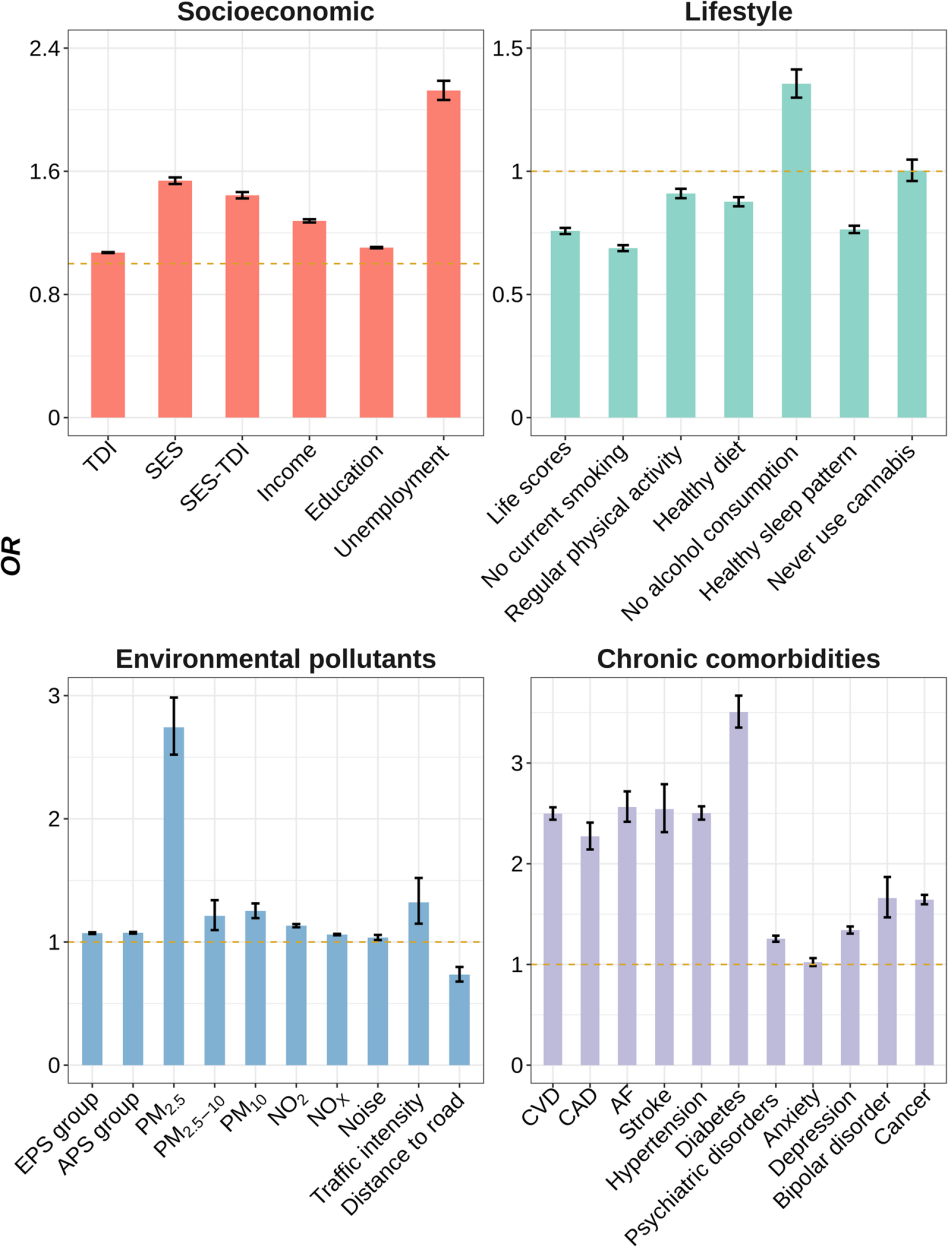
Bar plots indicating socioeconomic, lifestyle, environmental pollution, and chronic comorbidity factors on infectious diseases in participants from UK biobank. Odds ratios (*ORs*) were adjusted for age, sex, ethnic and assessment center. Dashed line represents no significant association. *TDI* Townsend deprivation, *SES* socioeconomic status, *EPS* environment pollution score, *APS* air pollution score, *PM*_*2.5*_ particulate matter ≤ 2.5 μm, *PM*_*2.5–10*_ particulate matter 2.5–10 μm, *PM*_*10*_ particulate matter ≤ 10 μm, *NO*_*x*_ nitrogen oxides, *NO*_*2*_ nitrogen dioxide, *CVD* cardiovascular disease, *CAD* cardiovascular diseases, *AF* atrial fibrillation

Among 412,258 participants included, 60,771 (14.7%) were diagnosed with infectious diseases during follow-up. We observed significant associations of infectious diseases with all socioeconomic factors (*P* < 0.0001) (Table 2 and Fig. 2). Both higher TDI (*OR* = 1.0720, 95% *CI*: 1.0690–1.0750) and lower individual level SES (*OR* = 1.5385, 95% *CI*: 1.5174–1.5600; *OR* = 1.4441, 95% *CI*: 1.4237–1.4649, additionally adjusted for TDI) were potential risk factors for infectious diseases. Participants with lower income level (*OR* = 1.2779, 95% *CI*: 1.2675–1.2883) or education qualifications (*OR* = 1.1041, 95% *CI*: 1.0998–1.1085), and those in unemployed status (*OR* = 2.1245, 95% *CI*: 2.0630–2.1876) were all at higher risk of infection. We further evaluated the associations in several common types of infectious diseases subgroups, and found that lower SES stands risk factors for all selected subtypes of infectious diseases, which also remained significant in the corresponding PSM cohorts, as well as the cohort comprised of participants enrolled in 2010 (Additional file 1: Tables S4–S8 and Additional file 2: Fig. S3).

**Table 2:** Associations of SES, lifestyle, environmental pollution and chronic comorbidity factors with infectious diseases in UK Biobank participants under multivariate linear regression

Variable	Description		*OR* (95% *CI*)	*P*
	Infection (*N*_1_ = 60,771)	Non-infection (*N*_2_ = 351,487)		
TDI	− 1.7577 (− 3.4268, 1.3194)	− 2.2374 (− 3.6969, 0.2835)	1.0720 (1.0690–1.0750)	< 0.0001
SES ^a^				
High	7276 (12.0)	73,673 (21.0)	1.5385 (1.5174–1.5600)	< 0.0001
Medium	28,383 (46.7)	187,584 (53.4)	1.4441 (1.4237–1.4649)	< 0.0001
Low	25,112 (41.3)	90,230 (25.7)		
Income (£) ^a^				
> 100,000	1832 (3.0)	20,590 (5.9)	1.2779 (1.2675–1.2883)	< 0.0001
52,000–100,000	8242 (13.6)	75,971 (21.6)		
31,000–51,999	13,388 (22.0)	94,445 (26.9)		
18,000–30,999	16,744 (27.6)	88,170 (25.1)		
< 18,000	20,565 (33.8)	72,311 (20.6)		
Education ^a^				
College or University degree	16,525 (27.2)	128,156 (36.5)	1.1041 (1.0998–1.1085)	< 0.0001
A levels/AS levels or equivalent	5926 (9.8)	42,123 (12.0)		
O levels/GCSEs or equivalent	12,663 (20.8)	75,061 (21.4)		
CSEs or equivalent	3124 (5.1)	19,160 (5.5)		
NVQ or HND or HNC or equivalent	4866 (8.0)	22,444 (6.4)		
Other professional qualifications	3473 (5.7)	17,471 (5.0)		
None of the above	14,194 (23.4)	47,072 (13.4)		
Unemployed ^a^	6767 (11.1)	23,494 (6.7)	2.1245 (2.0630–2.1876)	< 0.0001
Lifestyle scores ^a^				
0–1	13,607 (34.2)	63,415 (25.3)	0.7576 (0.7454–0.7701)	< 0.0001
2–3	19,409 (48.8)	139,646 (55.7)		
4–6	6739 (17.0)	47,602 (19.0)		
Lifestyle factors				
No current smoking ^a^	30,928 (51.1)	212,488 (60.6)	0.6879 (0.6760–0.7001)	< 0.0001
Regular physical activity ^a^	13,416 (22.6)	82,034 (23.6)	0.9096 (0.8907–0.9289)	< 0.0001
Healthy diet ^a^	13,271 (22.1)	83,299 (23.9)	0.8762 (0.8578–0.8949)	< 0.0001
No alcohol consumption ^a^	3018 (5.0)	12,532 (3.6)	1.3550 (1.2988–1.4133)	< 0.0001
Healthy sleep pattern ^a^	16,945 (29.9)	119,493 (36.4)	0.7640 (0.7492–0.7790)	< 0.0001
Never use cannabis ^a^	11,688 (79.1)	94,522 (76.6)	1.0029 (0.9605,1.0473)	0.8972
APS group ^a^				
Q1	9977 (17.8)	65,524 (20.4)	1.0744 (1.0674–1.0814)	< 0.0001
Q2	10,822 (19.3)	64,679 (20.1)		
Q3	11,372 (20.3)	64,129 (19.9)		
Q4	11,624 (20.8)	63,877 (19.9)		
Q5	12,138 (21.7)	63,364 (19.7)		
EPS group ^a^				
Q1	9988 (17.9)	65,513 (20.4)	1.0717 (1.0647–1.0787)	< 0.0001
Q2	10,822 (19.3)	64,679 (20.1)		
Q3	11,492 (20.5)	64,009 (19.9)		
Q4	11,538 (20.6)	63,963 (19.9)		
Q5	12,093 (21.6)	63,409 (19.7)		
Environmental pollutants				
PM_2.5_	9.9900 (9.3500, 10.6300)	9.9100 (9.2700, 10.5400)	2.7429 (2.5207–2.9844)	< 0.0001
PM_2.5–10_	6.1100 (5.8500, 6.6500)	6.1000 (5.8400, 6.6200)	1.2121 (1.0965–1.3394)	0.0002
PM_10_	16.0400 (15.2900, 17.0300)	16.0100 (15.2200, 16.9800)	1.2520 (1.1937–1.3130)	< 0.0001
NO_2_	26.5200 (21.9200, 31.4700)	26.0300 (21.2700, 31.0800)	1.1323 (1.1192–1.1455)	< 0.0001
NO_X_	43.0300 (35.1600, 51.5600)	42.1200 (34.0700, 50.4700)	1.0603 (1.0545–1.0660)	< 0.0001
Noise	55.1050 (53.6733, 57.1833)	55.0833 (53.6533, 57.1533)	1.0361 (1.0152–1.0574)	0.0006
Traffic intensity	17,053 (12,669, 25,324)	17,044 (12,574, 25,136)	1.3214 (1.1486–1.5198)	< 0.0001
Distance to road	373.1343 (164.7446, 735.2941)	380.2281 (168.0672, 757.5758)	0.7354 (0.6785–0.7972)	< 0.0001
Chronic comorbidities				
CVD ^a^	11,643 (19.2)	25,501 (7.3)	2.4982 (2.4374–2.5604)	< 0.0001
CAD ^a^	1754 (2.9)	3554 (1.0)	2.2716 (2.1416–2.4087)	< 0.0001
AF ^a^	1827 (3.0)	3253 (0.9)	2.5632 (2.4165–2.7181)	< 0.0001
Stroke ^a^	685 (1.1)	1349 (0.4)	2.5416 (2.3136–2.7896)	< 0.0001
Hypertension ^a^	9806 (16.1)	20,895 (5.9)	2.5030 (2.4377–2.5700)	< 0.0001
Diabetes ^a^	3423 (5.6)	4956 (1.4)	3.5073 (3.3521–3.6693)	< 0.0001
Psychiatric disorders ^a^	9730 (16.0)	50,375 (14.3)	1.2551 (1.2252–1.2857)	< 0.0001
Anxiety ^a^	3322 (5.5)	20,368 (5.8)	1.0230 (0.9846–1.0627)	0.2422
Depression ^a^	7849 (12.9)	38,570 (11.0)	1.3412 (1.3061–1.3770)	< 0.0001
Bipolar disorder ^a^	343 (0.6)	1292 (0.4)	1.6581 (1.4675–1.8685)	< 0.0001
Cancer ^a^	7241 (11.9)	24,081 (6.9)	1.6436 (1.5976–1.6907)	< 0.0001

Odds ratios (*OR*) and *P* values were adjusted for age, sex, ethnic and assessment center

*TDI* Townsend deprivation, *SES* socioeconomic status, *APS* air pollution score, *EPS* environment pollution score, *PM*_*2.5*_ particulate matter ≤ 2.5 μm, *PM*_*2.5-10*_ particulate matter 2.5–10 μm, *PM*_*10*_ particulate matter ≤ 10 μm, *NOx* nitrogen oxides, *NO*_*2*_ nitrogen dioxide, *CVD* cardiovascular disease, *CAD* cardiovascular diseases, *AF* atrial fibrillation

^a^Categorical variables are presented as number (percentage)

^b^Continuous variables are presented as median (quartile)

Moreover, we directly evaluated the associations of infection with other three baseline variables: lifestyle, environmental pollution and chronic comorbidities (Table 2, Additional file 1: Table S9 and Fig. 2). We found that adhering to healthier lifestyle (*OR* = 0.7576, 95% *CI*: 0.7454–0.7701) had protective effects, with only exception of fewer alcohol consumption (*OR* = 1.3550, 95% *CI*: 1.2988–1.4133). Heavier environmental pollution (*OR* = 1.0744, 95% *CI*: 1.0674–1.0814), including higher APS, PM_2.5_, PM_2.5–10_, PM_10_, NO_x_, and NO_2_, higher sound level of noise pollution, heavier traffic intensity and living closer to the main road resulted in higher infection risk. In addition, participants ever diagnosed of CVD (*OR* = 2.4982, 95% *CI*: 2.4374–2.5604), diabetes (*OR* = 3.5073, 95% *CI*: 3.3521–3.6693), psychiatric disorders (*OR* = 1.2551, 95% *CI*: 1.2252–1.2857) or cancer (*OR* = 1.6436, 95% *CI*: 1.5976–1.6907) had a higher infection risk.

We also performed subgroup analysis in each infectious diseases subtypes cohorts, and found that most results kept largely consistent with the main analyses (Additional file 2: Fig. S3 and Additional file 1: Tables S4–S7 and S9). Notably, lower rates of cannabis use (*OR* = 0.2583, 95% *CI*: 0.1962–0.3389; *OR* = 0.3177, 95% *CI*: 0.2340–0.4296, in matching cohort) were associated with lower risk of blood or sexually transmitted infectious diseases specifically, while associations of regular physical activity (*OR* = 0.9081, 95% *CI*: 0.8725–0.9449; *OR* = 0.8967, 95% *CI*: 0.8586–0.9363, in matching cohort) and bipolar disorder (*OR* = 1.8593, 95% *CI*: 1.4995–2.2774; *OR* = 2.0183, 95% *CI*: 1.5726–2.5733, in matching cohort) remained significant only in digestive infection subgroup. Sensitivity analysis in participants enrolled in 2010 also showed similar results (Additional file 2: Fig. S3 and Additional file 1: Tables S8–S9).

### Mediation effects of lifestyle and environmental pollution on SES to infectious diseases

**Fig. 3:**
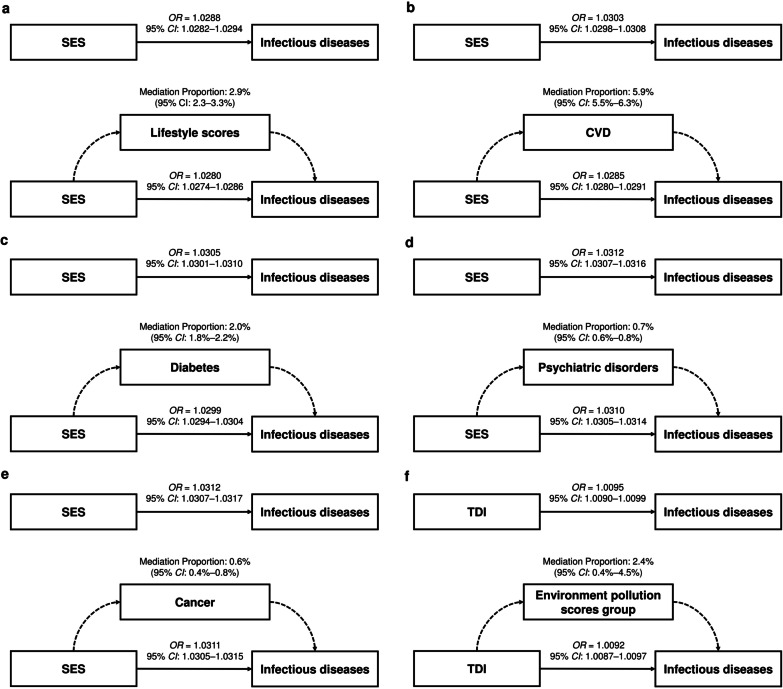
Mediation effects of SES on infectious diseases by Lifestyle scores (**a**), CVD (**b**), diabetes (**c**), psychiatric disorders (**d**), and cancer (**e**), and TDI by EPS (**f**). Regression analyses of SES on mediators, and mediators on infection were all adjusted for age, sex, ethnic and assessment center. *TDI* Townsend deprivation, *SES* socioeconomic status, *CVD* cardiovascular disease

Considering the significant correlations between socioeconomic factors and lifestyle, environmental pollution, or chronic comorbidity factors (Additional file 2: Fig. S2), we hypothesized that they may mediate partly the effect of SES on infectious diseases. With the lifestyle score additionally adjusted, an *OR* of SES on infection dropped to 1.4895 (95% *CI*: 1.4646–1.5148), and the proportion mediated by lifestyle score was 2.9% (95% *CI*: 2.6–3.3%) (Table 3 and Fig. 3a), which may mainly come from no current smoking (5.1%, 95% *CI*: 4.7–5.5%) and healthy sleep pattern (3.3%, 95% *CI*: 3.0–3.7%) (Additional file 1: Table S10). In infection subtypes cohorts, we observed similar patterns, and the mediation proportions by lifestyle score ranged from 2.9% (95% *CI*: 2.3–3.7%, for digestive infectious diseases) to 4.1% (95% *CI*: 2.1–6.6%, for respiratory infectious diseases) (Table 3).

**Table 3:** Mediation effects of socioeconomic factors on infectious diseases by lifestyle, environmental pollution or chronic comorbidity factors in UK Biobank

Outcome	Exposure	Mediator	Effect with mediator adjusted (95% *CI*)	Indirect effect by mediator (95% *CI*)	Mediation proportion (%) (95% *CI*)	*P*
Infectious diseases	SES	Lifestyle scores	1.4895 (1.4646–1.5148)	1.0008 (1.0008–1.0009)	2.9 (2.6–3.3)	< 0.001
		CVD	1.4815 (1.4610–1.5023)	1.0018 (1.0016–1.0019)	5.9 (5.5–6.3)	< 0.001
		Diabetes	1.5081 (1.4873–1.5291)	1.0006 (1.0005–1.0007)	2.0 (1.8–2.2)	< 0.001
		Psychiatric disorders	1.5338 (1.5127–1.5551)	1.0002 (1.0002–1.0003)	0.7 (0.6–0.8)	< 0.001
		Cancer	1.5366 (1.5155–1.5580)	1.0002 (1.0001–1.0002)	0.6 (0.3–0.8)	< 0.001
	TDI	EPS group	1.0708 (1.0673–1.0743)	1.0002 (1.0000–1.0004)	2.3 (0.3–4.3)	0.014
Respiratory infectious diseases	SES	Lifestyle scores	1.4835 (1.3508–1.6302)	1.0016 (1.0008–1.0024)	4.1 (2.1–6.6)	< 0.001
		CVD	1.4757 (1.3656–1.5953)	1.0027 (1.0019–1.0037)	6.8 (4.7–9.8)	< 0.001
		Diabetes	1.5103 (1.3982–1.6321)	1.0009 (1.0004–1.0014)	2.2 (1.1–3.6)	< 0.001
		Psychiatric disorders	1.5465 (1.4323–1.6706)	1.0001 (0.9997–1.0004)	0.2 (− 0.7–1.0)	0.576
		Cancer	1.5485 (1.4339–1.6730)	1.0001 (0.9993–1.0007)	0.2 (− 1.7–1.7)	0.804
	TDI	EPS group	1.0606 (1.0418–1.0797)	1.0015 (1.0001–1.0028)	12.7 (1.4–25.7)	0.028
Digestive infectious diseases	SES	Lifestyle scores	1.4466 (1.3971–1.4979)	1.0011 (1.0009–1.0013)	2.9 (2.3–3.7)	< 0.001
		CVD	1.4313 (1.3907–1.4732)	1.0025 (1.0021–1.0028)	6.4 (5.5–7.4)	< 0.001
		Diabetes	1.4511 (1.4100–1.4936)	1.0011 (1.0009–1.0013)	2.7 (2.2–3.3)	< 0.001
		Psychiatric disorders	1.4830 (1.4413–1.5261)	1.0003 (1.0001–1.0005)	0.7 (0.3–1.2)	< 0.001
		Cancer	1.4851 (1.4433–1.5283)	1.0002 (1.0000–1.0004)	0.5 (0.1–1.0)	0.018
	TDI	EPS group	1.0641 (1.0571–1.0711)	0.9999 (0.9994–1.0003)	− 1.4 (− 6.2–3.1)	0.584
Blood or sexually transmitted infectious diseases	SES	Lifestyle scores	2.4246 (2.0957–2.8124)	1.0015 (1.0004–1.0027)	3.3 (1.0–6.0)	0.004
		CVD	2.3007 (2.0435–2.5945)	1.0010 (1.0005–1.0017)	2.0 (1.1–3.5)	< 0.001
		Diabetes	2.3386 (2.0780–2.6363)	1.0006 (1.0001–1.0012)	1.1 (0.3–2.5)	0.002
		Psychiatric disorders	2.3447 (2.0844–2.6422)	1.0011 (1.0003–1.0020)	2.2 (0.6–4.1)	0.012
		Cancer	2.3866 (2.1208–2.6905)	1.0005 (0.9996–1.0014)	1.1 (− 0.8–2.9)	0.242
	TDI	EPS group	1.1350 (1.1056–1.1654)	0.9995 (0.9972–1.0018)	− 2.6 (− 14.5–9.2)	0.642
Infectious diseases in 2010	SES	Lifestyle scores	1.4439 (1.3864–1.5039)	1.0005 (1.0004–1.0007)	2.0 (1.5–2.8)	< 0.001
		CVD	1.4129 (1.3657–1.4618)	1.0019 (1.0016–1.0022)	7.1 (6.1–8.4)	< 0.001
		Diabetes	1.4400 (1.3920–1.4896)	1.0006 (1.0005–1.0008)	2.3 (1.8–2.9)	< 0.001
		Psychiatric disorders	1.4642 (1.4157–1.5145)	1.0002 (1.0002–1.0004)	0.9 (0.6–1.3)	< 0.001
		Cancer	1.4656 (1.4170–1.5161)	1.0003 (1.0001–1.0005)	1.0 (0.2–1.7)	0.014
	TDI	EPS group	1.0617 (1.0531–1.0704)	0.9999 (0.9995–1.0002)	− 1.5 (− 7.0–3.4)	0.568

Effect and proportion estimations and *P* values were adjusted for age, sex, ethnic and assessment center

*TDI* Townsend deprivation, *SES* socioeconomic status, *EPS* environment pollution score, *CVD* cardiovascular disease

As for the chronic comorbidity factors, it showed that a history of CVD mediated the largest proportion in the effect of SES on infection (5.9%, 95% *CI*: 5.5–6.3%) (Table 3 and Fig. 3b–e), which ranged from 2.0% (95% *CI*: 1.1–3.5%, for blood or sexually transmitted infectious diseases) to 6.8% (95% *CI*: 4.7–9.8%, for respiratory infectious diseases). The mediation effects by CVD may mainly come from hypertension (Additional file 1: Table S10). Note that psychiatric disorders mediated the largest proportion of 2.2% (95% *CI*: 0.6–4.1%) in blood or sexually transmitted infectious diseases, and the main contributor may be depression (Table 3 and Additional file 1: Table S10).

We also explored the effects of area TDI mediated by EPS. The results showed that the proportion mediated by EPS was 2.3% (95% *CI*: 0.3–4.3%) (Table 3 and Figs. 3f), which may mainly come from PM_2.5_ (Additional file 1: Table S10). Of note, EPS showed specific mediation effects on SES to respiratory infectious diseases (12.7%, 95% *CI*: 1.4–25.8%) in subgroup analysis (Table 3 and Additional file 1: Table S10).

In addition, sensitivity analysis in 2010 subgroup also supported the results above (Table 3).

### Interaction and joint analysis of lifestyle, environmental pollution, or chronic comorbidity factors and SES on infectious diseases

**Fig. 4:**
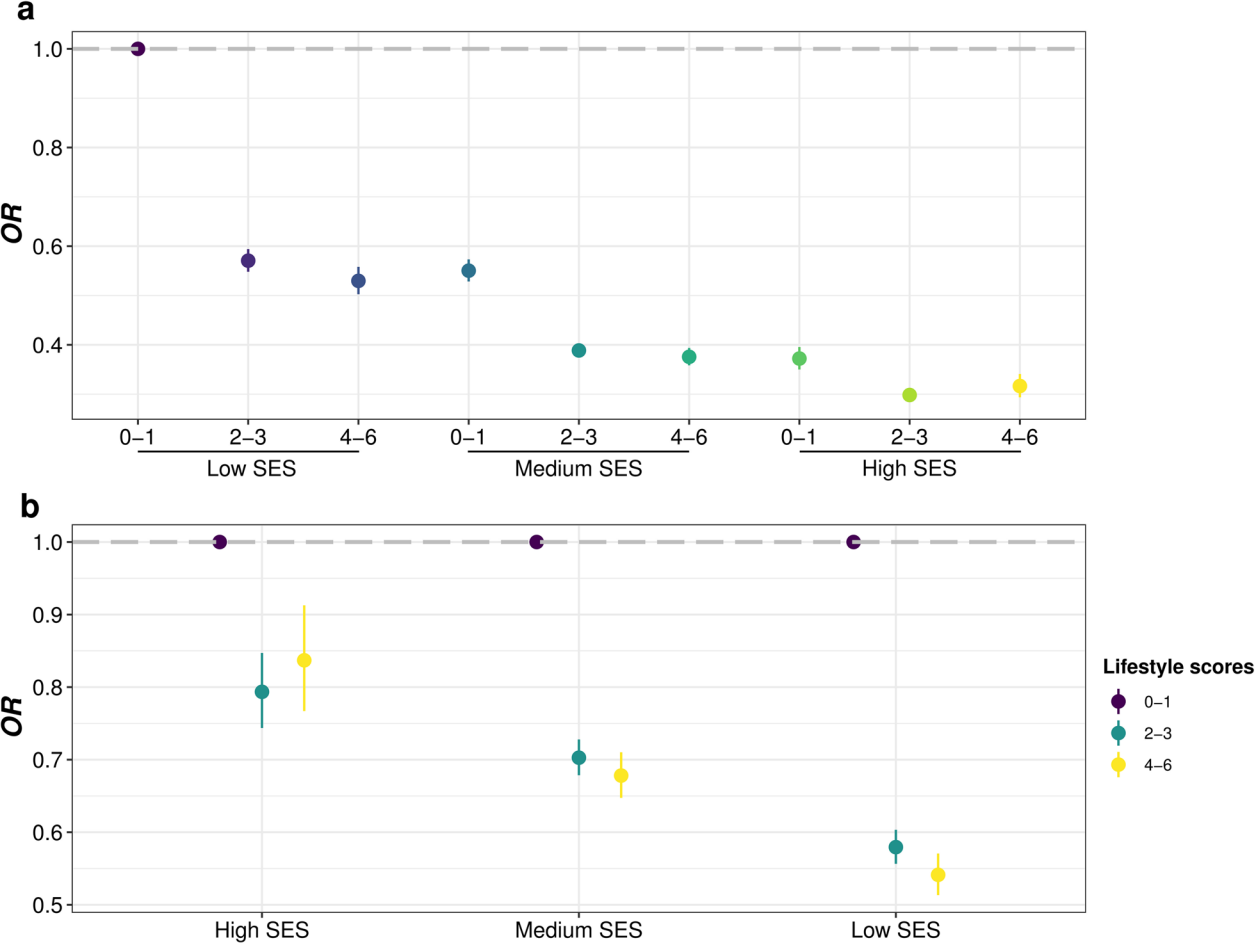
Forest plot indicating lifestyle scores on infectious diseases in different SES subgroups from UK biobank. The group with low SES and poor lifestyle scores (0–1) was selected as the overall control group (**a**), or for each SES subgroup individually, that with poor lifestyle scores (0–1) was selected as the control group (**b**). Odds ratios (*ORs*) were adjusted for age, sex, ethnic and assessment center. Dashed line represents no significant association. *SES* socioeconomic status

In order to evaluate the impact of socioeconomic status on the effects of other risk factors, we performed a series of interaction and joint analysis. First, we observed negative interaction effect between SES and lifestyle score (*OR* = 0.8699, 95% *CI*: 0.8492–0.8912), as well as several individual factors including no current smoking, regular physical activity, healthy diet and healthy sleep pattern, on infectious diseases (Table 4 and Additional file 1: Table S11), suggesting that adhering to a healthier lifestyle may alleviate the risk effect of lower SES on infection. We further explored theses interactions in each subtype cohort, but only got consistent results in digestive infectious diseases cohort, with regular physical activity significant in respiratory infection cohort, while never use cannabis significant specifically in blood or sexually transmitted infectious diseases (Table 4 and Additional file 1: Table S11). The joint analysis showed that participants with both higher socioeconomic status and healthier lifestyles had much lower risk of infection (Additional file 1: Table S12, Fig. 4a, and Additional file 2: Figs. S6a, S11a, and S16a), while in low SES subgroups higher lifestyle scores exhibited stronger protective effects on infection (Additional file 1: Tables S12–S13, Fig. 4b, and Additional file 2: Figs. S6b, S11b, and S16b), in agreement with the interaction results above.

**Table 4:** Interaction between SES and lifestyle, environmental pollution or chronic comorbidity factors on infectious diseases in UK Biobank

Outcome	Variable	SES		SES-TDI	
		*OR* (95% *CI*)	*P*	*OR* (95% *CI*)	*P*
Infectious diseases	Lifestyle scores	0.8699 (0.8492, 0.8912)	< 0.0001	0.8800 (0.8591, 0.9014)	< 0.0001
	EPS group	1.0325 (1.0225, 1.0426)	< 0.0001	1.0285 (1.0186, 1.0385)	< 0.0001
	CVD	0.9792 (0.9420, 1.0179)	0.2874	0.9662 (0.9296, 1.0044)	0.0818
	Diabetes	0.9797 (0.9095, 1.0557)	0.5886	0.9617 (0.8929, 1.0363)	0.3040
	Psychiatric disorders	0.9916 (0.9564, 1.0282)	0.6473	0.9890 (0.9541, 1.0253)	0.5480
	cancer	0.9047 (0.8664, 0.9447)	< 0.0001	0.9097 (0.8714, 0.9499)	< 0.0001
Respiratory infectious diseases	Lifestyle scores	0.8792 (0.7695, 1.0045)	0.0582	0.8879 (0.7776, 1.0140)	0.0792
	EPS group	1.0462 (0.9905, 1.1049)	0.1058	1.0423 (0.9872, 1.1005)	0.1351
	CVD	1.0806 (0.8752, 1.3382)	0.4743	1.0627 (0.8608, 1.3160)	0.5740
	Diabetes	0.9574 (0.6428, 1.4396)	0.8320	0.9628 (0.6458, 1.4486)	0.8536
	Psychiatric disorders	1.1490 (0.9372, 1.4124)	0.1842	1.1447 (0.9345, 1.4062)	0.1945
	cancer	0.8180 (0.6492, 1.0333)	0.0900	0.8306 (0.6593, 1.0491)	0.1170
Digestive infectious diseases	Lifestyle scores	0.9000 (0.8565, 0.9458)	< 0.0001	0.9070 (0.8632, 0.9529)	0.0001
	EPS group	1.0331 (1.0128, 1.0537)	0.0013	1.0294 (1.0094, 1.0499)	0.0039
	CVD	1.0101 (0.9324, 1.0946)	0.8068	0.9994 (0.9227, 1.0829)	0.9887
	Diabetes	0.9785 (0.8406, 1.1403)	0.7797	0.9626 (0.8268, 1.1218)	0.6241
	Psychiatric disorders	0.9467 (0.8811, 1.0173)	0.1350	0.9449 (0.8797, 1.0153)	0.1216
	cancer	0.8949 (0.8185, 0.9789)	0.0150	0.8984 (0.8219, 0.9825)	0.0186
Blood or sexually transmitted infectious diseases	Lifestyle scores	0.9455 (0.7625, 1.1729)	0.6096	0.9712 (0.7853, 1.2016)	0.7873
	EPS group	1.0424 (0.9564, 1.1355)	0.3428	1.0385 (0.9545, 1.1291)	0.3782
	CVD	0.6587 (0.4571, 0.9591)	0.0269	0.6644 (0.4604, 0.9685)	0.0308
	Diabetes	0.5265 (0.2871, 0.9743)	0.0382	0.5722 (0.3097, 1.0632)	0.0740
	Psychiatric disorders	1.0111 (0.7649, 1.3447)	0.9389	1.0051 (0.7608, 1.3358)	0.9719
	cancer	0.6344 (0.4380, 0.9267)	0.0171	0.6508 (0.4493, 0.9499)	0.0242
Infectious diseases in 2010	Lifestyle scores	0.8681 (0.8186, 0.9205)	< 0.0001	0.8743 (0.8247, 0.9269)	< 0.0001
	EPS group	1.0225 (0.9981, 1.0475)	0.0711	1.0260 (1.0017, 1.0510)	0.0358
	CVD	0.9456 (0.8649, 1.0344)	0.2205	0.9337 (0.8541, 1.0212)	0.1321
	Diabetes	0.9412 (0.7931, 1.1194)	0.4900	0.9266 (0.7810, 1.1017)	0.3847
	Psychiatric disorders	0.9875 (0.9052, 1.0777)	0.7774	0.9861 (0.9042, 1.0758)	0.7514
	cancer	0.8488 (0.7669, 0.9401)	0.0016	0.8534 (0.7712, 0.9450)	0.0022

Effect and proportion estimations and *P* values were adjusted for age, sex, ethnic and assessment center

*TDI* Townsend deprivation, *SES* socioeconomic status, *EPS* environment pollution score, *CVD* cardiovascular disease

**Fig. 5:**
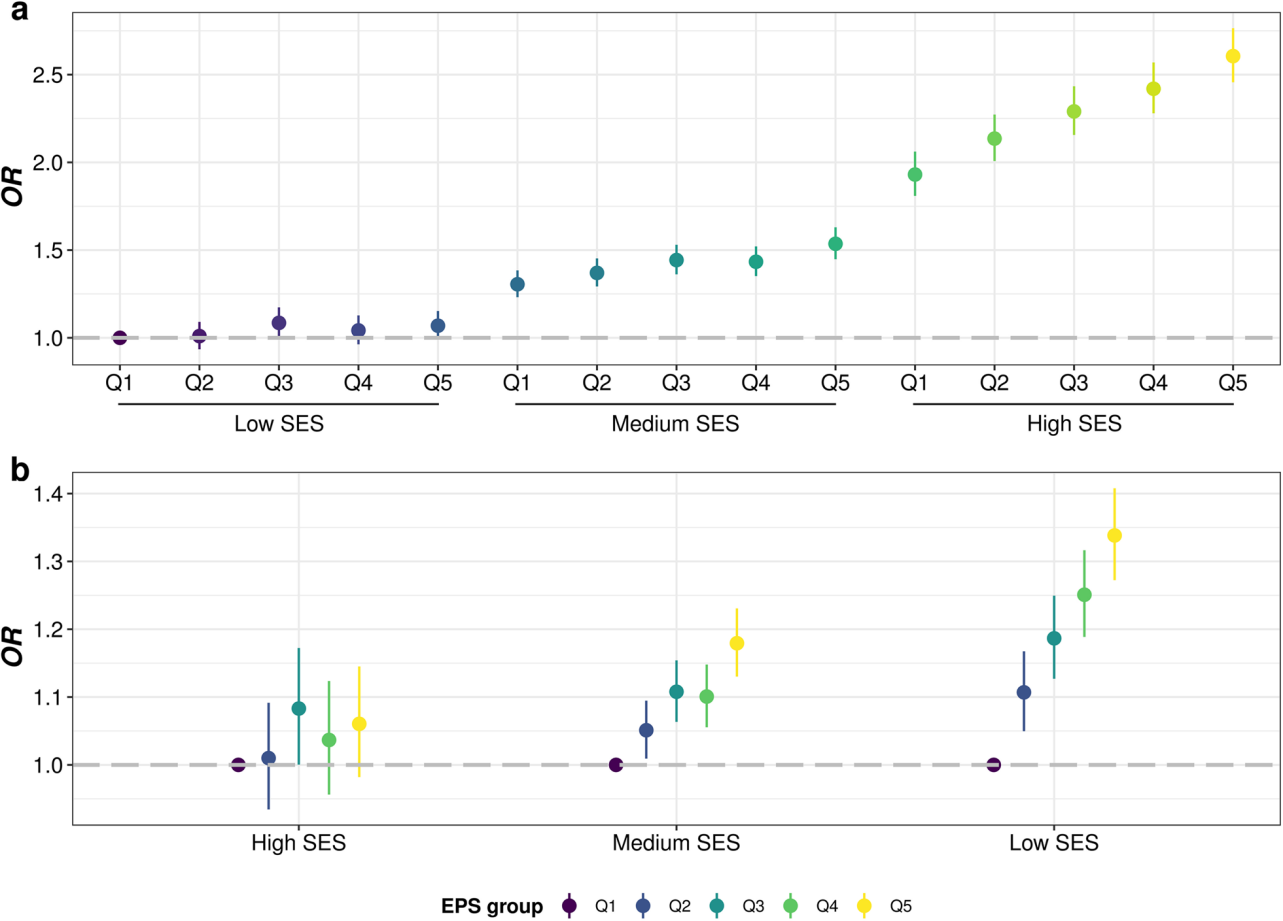
Forest plot indicating environmental pollution score (EPS) groups on infectious diseases in different SES subgroups from UK biobank. The group with high SES and low EPS (top fifth, Q1) was selected as the overall control group (**a**), or for each SES subgroup individually, that with low EPS (Q1) was selected as the control group (**b**). Odds ratios (*ORs*) were adjusted for age, sex, ethnic and assessment center. Dashed line represents no significant association. *SES* socioeconomic status

Second, in terms of air pollution factors, we observed significant synergy effects between SES and EPS on infectious diseases (*OR* = 1.0325, 95% *CI*: 1.0225–1.0426) and digestive infection (*OR* = 1.0331, 95% *CI*: 1.0128–1.0537) (Table 4). Some individual factors, including PM_2.5_, PM_10_, NO_2_, NO_X_ and traffic intensity, as well as the APS also showed similar results (Additional file 1: Table S11). In joint analysis, we also found that participants with lower SES and living in heavier environmental pollution were in much higher risk of infection, with increase of infection risk across pollution groups also more pronounced in the low SES group (Additional file 1: Table S14–S15, Fig. 5, and Additional file 2: Fig. S4). In addition, APS, PM_2.5_, PM_10_, NO_2_, NO_X_, and traffic intensity tend to show higher risk effects on infection in low SES subgroups (Additional file 2: Fig. S5), consistent with the observed synergy effects and indicating that poor individual socioeconomic status may further aggravate the risk effects of environmental pollution on infection. Similar trends of EPS, APS, PM_2.5_, NO_2_, and NO_x_ were also observed in each subtype cohort (Additional file 1: Tables S11, S13–S15, and Additional file 2: Figs. S7–S9, S12–S14, and S17–S19).

**Fig. 6:**
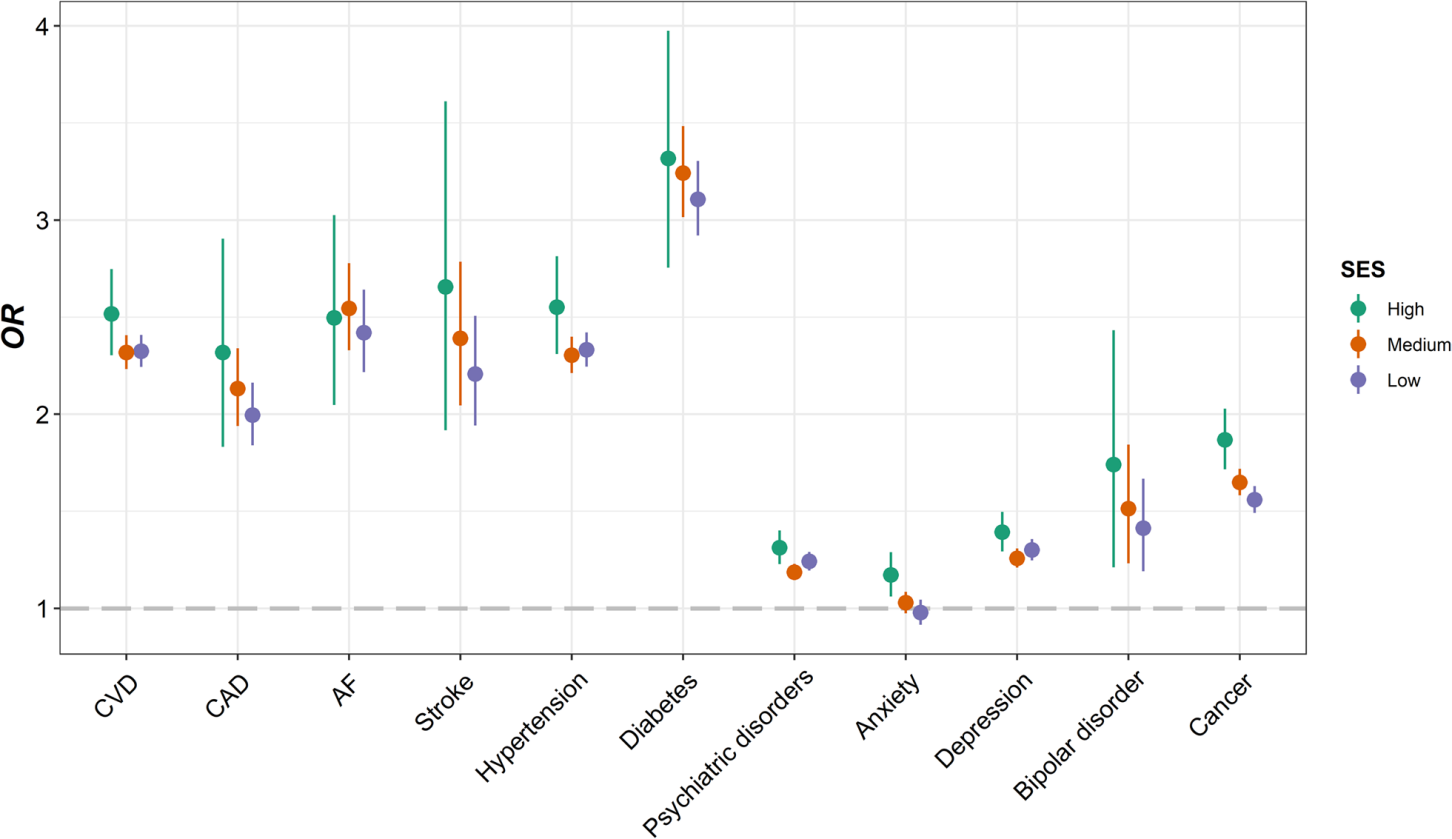
Forest plot indicating chronic comorbidity factors on infectious diseases in different socioeconomic status (SES) subgroups from UK biobank. Odds ratios (*ORs*) were adjusted for age, sex, ethnic and assessment center. Dashed line represents no significant association. *SES* socioeconomic status, *CVD* cardiovascular disease, *CAD* cardiovascular diseases, *AF* atrial fibrillation

Third, in term of chronic comorbidity factors, only a history of cancer showed significant negative interaction with SES on infectious diseases (*OR* = 0.9047, 95% *CI*: 0.8664–0.9447) (Table 4, and Additional file 1: Table S11). Interestingly, however, nearly all types of chronic comorbidity factors showed higher risk effect in higher SES subgroup (Additional file 1: Table S13 and Fig. 6), among which cancer showed similar trend pattern in different subtypes cohorts (Additional file 1: Table S13 and Additional file 2: Figs. S10, S15, and S20).

Finally, we repeated all these analysis with additional adjustment for area SES (i.e., TDI), and also performed subgroup analysis in the 2010 subgroup. Most results remained relatively robust (Additional file 1: Tables S11 and S13, and Additional file 2: Figs. S21–S25).

### Effects of socioeconomic inequity on infection in different sex and ethnic subgroups

**Fig. 7:**
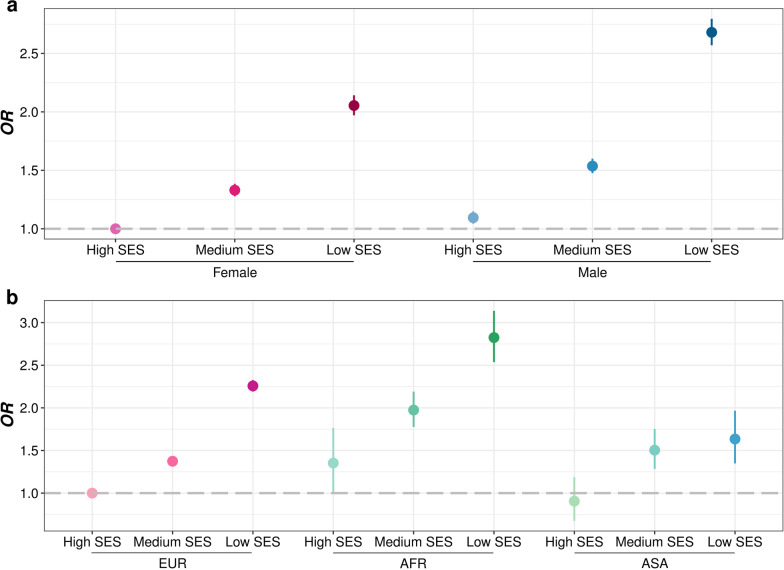
Forest plot indicating risk of infectious diseases in different sex (**a**) or ethnic (**b**) by SES subgroups from UK biobank. Odds ratios (*ORs*) were adjusted for age, sex (analysis on ethnic), ethnic (analysis on sex) and assessment center. Dashed line represents no significant association. *SES* socioeconomic status, *EUR* European, *AFR* African, *ASA* Asian

We also found that males (*OR* = 1.1498, 95% *CI*: 1.1300–1.1700) and African (AFR) people (*OR* = 1.5333, 95% *CI*: 1.4273–1.6455) had higher risk of infection compared with females and European (EUR) people, respectively (Additional file 1: Table S16). We further explored the effects of socioeconomic inequity on infection in different sex and ethnic subgroups (Additional file 1: Table S16). We observed relative higher risk effect of low SES on infections in males (*OR* = 1.5733, 95% *CI*: 1.5433–1.6039) than females (*OR* = 1.5094, 95% *CI*: 1.4798–1.5397). The joint analysis also showed that male with lower SES tend to have much higher risk of infection (Additional file 1: Table S17 and Fig. 7a). Interestingly, we observed a higher risk effect of low SES in EUR people (*OR* = 1.5412, 95% *CI*: 1.5194–1.5633) than AFR (*OR* = 1.4384, 95% *CI*: 1.2793–1.6191) and Asian (ASA, *OR* = 1.2888, 95% *CI*: 1.1027–1.5087) people, indicating that SES gap brings less differences of infection risk in AFR and ASA people. However, in the joint analysis, AFR people showed higher infection risk than EUR people across all socioeconomic status, though this was not observed in ASA people (Additional file 1: Table S17 and Fig. 7b). The mediation effects of EPS on TDI were also significant only in the male (4.0%, 95% *CI*: 1.7–6.7%) and EUR (2.6%, 95% *CI*: 0.5–4.5%) subgroups. In addition, it showed that the mediation effects of lifestyle scores on SES were not significant in either the AFR or ASA subgroups. The interaction direction and strength between SES and lifestyle scores, EPS, and those chronic comorbidity factors were relatively consistent across subgroups.

### Validation using US NHANES data

We totally included 45,671 sampled participants from US NHANES (Fig. 1b). The participants have a mean age of 47.35 ± 16.87 years, among whom 23,360 (50.2% weighted) were women, 13,809 (29.2% weighted) were of high SES, 18,284 (38.7% weighted) of medium SES, and 15,218 (32.2% weighted) of low SES. Participants with low SES were more likely to be female, non-white, and older. Low SES also tends to be associated with poorer lifestyle and a higher rate of chronic comorbidities (Additional file 1: Table S18).

Laboratory tests showed that 10,434 (23.2% weighted) participants had at least one infectious disease when surveyed. And participants with lower SES (*OR* = 1.2471, 95% *CI*: 1.1817–1.3161), or comorbid psychiatric disorders (*OR* = 1.9025, 95% *CI*: 1.0984–3.2951) had a higher risk of infections, and those adhering to healthier lifestyle were in lower risk of infections (*OR* = 0.5585, 95% *CI*: 0.5131–0.6079) (Additional file 1: Table S19), in agreement with the results of UKB. The mediated proportion by lifestyle score in the effects of SES on infections was up to 27.2% (95% *CI*: 27.1–27.3%), which mainly comes from no current smoking (35.5%, 95% *CI*: 33.5–37.5%) (Additional file 1: Table S20). SES also shows negative interaction effect with lifestyle score (*OR* = 0.8866, 95% *CI*: 0.8113–0.9688), no current smoking (*OR* = 0.8348, 95% *CI*: 0.7647–0.9113), and healthy diet (*OR* = 0.8418, 95% *CI*: 0.7423–0.9546) (Additional file 1: Table S21). And as expected, the joint analysis showed that participants with both higher SES and healthier lifestyles had much lower risk of infection, and adhering to healthy lifestyle can also bring stronger protective effects in participants with low SES (Additional file 1: Table S22 and Additional file 2: Fig. S26). No significant interaction was observed between SES and chronic comorbidity factors in US NHANES (Additional file 1: Tables S21 and S23 and Additional file 2: Fig. S27).

Both direct and indirect effects of SES show relatively consistent across sex subgroups, and males with lower SES also tend to have much higher risk of infection as observed in UKB (Additional file 1: Tables S24–S25 and Additional file 2: Fig. S28a). Notably, however, SES showed no significant associations with infection. We further observed that though non-white people with different SES tend to have similar infection risks, non-white people still showed higher infection risk than white people across all SES subgroups, suggesting that heavy infection burden may exist even in non-white people with high SES (Additional file 1: Tables S24–S25 and Additional file 2: Fig. S28b).

## Discussion

In this comprehensive analysis, we explored the associations between SES and infectious diseases in a large-scale prospective cohort data, and found that low SES was an important risk factor for infections, part of which may be mediated by poor lifestyle, heavy pollution in living environment, and chronic comorbidities. We also found significant interactions between SES and several lifestyles, environmental pollution and chronic comorbidity factors. We employed a series of sensitivity analyses, including to repeat our main analysis in an external data from the US, and obtained almost consistent results.

Although associations between SES and infectious diseases have got discussed before, previous studies usually defined SES from a single perspective, and focused only on some certain infections [51]. For example, Donnelly et al. used a national cohort in the US to examine the associations of neighborhood socioeconomic status (nSES) with risk of hospitalization for infection and sepsis, and found that participants residing in high-nSES neighborhoods have lower infection rates, where physical weakness and diabetes played certain mediation roles [52]. Another case–control study in Sweden also found that participants who were in unemployed status, had a lower level of educational attainment or income were more likely to be with invasive bacterial diseases, blood-borne infectious diseases, tuberculosis, and antibiotic-resistant infections [53]. However, it remained unclear whether SES has a consistent impact on the overall burden of infectious diseases. In our study, we used family income level, education qualification, employment status, and health insurance coverage to jointly define a SES, and evaluate the association between the composite SES with individual’s burden of infectious diseases. We found that people in low SES had a higher risk of overall infections, which showed much stronger in males and non-white people. We also evaluated each individual SES variable, and obtained comparable results with previous investigations, which may indicate the credibility of our results [52–55].

Recent studies have demonstrated that SES could influence an individual's lifestyle and a regional environment, and so as to affects the incidence of cardiovascular disease and allergic airway diseases [32, 56]. However, no studies have been conducted to assess the potential roles of lifestyle and environmental pollution in the association between SES and infectious diseases. We thus employed a mediation analysis in this study, and found that about 2.9% of the association between individual SES and infectious diseases could be explained by lifestyle. However, in subgroup analysis by ethnicity, the mediation role of lifestyle showed statistical significance only in EUR participants. And when we randomly sampled 8,000 participants from the EUR subgroup and re-performed the mediation analysis, we got a similar result (3.1%, 95% *CI*: 1.2–6.0%), which ruled out the case caused by the gap in sample size. We further explored the distribution of lifestyle factors across ethnic subgroups in UKB and were surprised to find that participants in the EUR subgroup were in a worse lifestyle compared with those in the AFR or ASA subgroups (*P* < 0.0001), which may mainly come from the much higher proportions of smokers and drinkers (Additional file 1: Table S26). It is important that a positive correlation between lifestyle scores and SES the was also only observed in the EUR subgroup, which may be an important cause of heterogeneity results in the mediation analysis (Additional file 1: Table S26). In addition, about 2.3% of the association between TDI and infections could be explained by environmental pollution, while the mediation role of environmental pollution showed statistical significance only in males, indicating that males may be more susceptible to the environmental pollution caused by low SES.

Previous study has demonstrated that risk of many adverse health-related outcomes, including infectious diseases, could be reduced through lifestyle modification [57–59]. In our study, we consistently found that adhering to a healthy lifestyle alone had a protective effect on overall infections across all SES subgroups. Importantly, the protective effect of healthy lifestyle showed much stronger among those with low SES, which indicated the potential modification effects of healthy lifestyle on poor SES, and emphasized the necessity to enhance health education, especially among those with low SES who were more vulnerable to infection. Similar trends have ever been observed in another two UKB-based studies, which evaluated the joint effects of socioeconomic and lifestyle factors on CVD and all-cause mortality [19, 32].

The associations between individual environmental pollutants and infection were usually established in some respiratory infectious diseases. For example, a study from Wuhan, China identified short-term exposure to NO_2_, sulfur dioxide (SO_2_), and ground-level ozone (O_3_) as risk factors of influenza incidence [60]. Several air pollutants such as PM_2.5_, PM_10_, SO_2_, NO_2_, and NO_x_ were also found to be associated with tuberculosis [26, 61, 62]. In this study, we created an EPS to assess the joint exposure to various environmental pollutants, and further expanded the associations to the overall burden of infectious diseases, as well as digestive and blood or sexually transmitted infections. In fact, that environmental pollutants can affect multiple organ systems is not a new topic in chronic disease research. And oxidative stress, systemic inflammation, and autonomic imbalance were usually widely accepted mechanisms [63, 64]. Previous studies have reported that exposure to environmental pollutants impacted both innate and adaptive immunity [65]. For example, increased monocyte and CD8 + T proportion but decreased B lymphocyte have been found in children from the polluted area [66]. Pollutants can stimulate the epithelium and macrophages to release inflammatory cytokines [65]. Another study also found that exposure to heavy NO_2_ and PM_10_ pollution resulted in a weakened type II interferon response, with the decrease in the Th1 pathway indicating impaired antiviral cellular effects [67]. In addition, epigenetic and transcriptome analysis also revealed alteration in gene expression and DNA methylation caused by exposure to NO_2_, NO_x_, and PM_2.5_, though conflicting evidence existed in their effects on circulating markers of inflammation [68–70]. Furthermore, we also observed significant synergy interaction between high EPS and low SES, suggesting that participants with low SES and living with heavy environmental pollution may at a much higher risk of infection, which was also confirmed by the results of the joint analysis. And we further observed a pronounced increase in infection risk across EPS groups in the low SES group, indicating that heavier environmental pollution may do bring more infection risk for participants with low SES.

In addition, we found participants with some chronic diseases were in higher infection risk. A notable point is that cancer history had a significant negative interaction with SES, and it showed higher risk effects in high SES subgroup, indicating that a cancer history may bring more risk for participants in high SES. Considering the cancer disparities across SES shown in our data (Table 1 and Additional file 2: Fig. S2) and reported previously [71], we hypothesized that it might be caused by the distribution disparities of cancer tumor behaviors among different SES subgroups. However, as we repeated the analysis using the recorded cancer tumor behavior in UKB, both association (*OR*: 1.1375, 95% *CI*: 1.1290–1.1460) and interaction (*OR*: 0.9730, 95% *CI*: 0.9620–0.9842) results remained consistent with the main analysis. A potential explanation may exist elsewhere like the genetic susceptibility [72–74]. Since we failed to validate it in the US NHANES, and few related studies are available now, the inherently complex relationship may wait for further exploration and validation. Certainly, those with low SES and with chronic comorbidities still have a much higher infection risk, indicating the necessity of enhanced focus on these individuals.

To the best of our knowledge, this study is the first to evaluate the contribution of socioeconomic factors to the overall infectious diseases. We also explore the underlying mediators that link SES with infections risk from the perspectives of lifestyle, environmental pollution, and chronic comorbidities. The main strength of our study might be the large sample size of the UKB and US NHANES, and the clear and standardized definitions on research variables. The relatively high consistency of the main results from the two cohorts made our findings quite robust. Apart from that, we constructed several composite scores in this study, which may provide a more comprehensive reflection on SES, lifestyle, and environmental pollution respectively, as compared with previous studies. In addition, we also considered different sex and ethnic subgroups, and successfully defined potential subpopulations in higher risk, which may further facilitate the implementation of more precise control measures in the future.

Nonetheless, our study still has some limitations. First, in the UKB, we only used the baseline survey data to define the socioeconomic and the lifestyle status of each participant. Although it can be helpful to confirm the temporal sequence of exposures and infections, it may lead to inaccurate estimates of associations for that SES and lifestyle of an individual may be changeable over time. Second, for the definition of environmental pollutants of the UKB, we treated the measurements in 2009 and 2010 as a proxy for chronic, long-term exposure estimation as previous studies did [43, 47], which may also lead to information bias. To account for this, we further conducted a sensitivity analysis using participants enrolled in 2010 only. Since the results were largely consistent, this proxy may be reasonability to some extent. Third, since each of the target infection subtype in the UKB only comprised a limited number of cases that took part a fairly small fraction of the entire cohort, the case–control imbalance may result in high false-positive rates. To account for this, we included a PSM cohort in sensitivity analyses, as described in the method section, and obtained relatively consistent results. Fourth, the external validation data used was from a cross-sectional study, which is less persuasive in association analysis, and differences existed in the data collection process and variable structure between the US NAHES and UKB. Although we have tried to harmonize the variable definitions as much as possible and did get similar results, we still should interpret them with caution. Fifth, information on socioeconomic factors and lifestyle factors in UKB and US NAHES was mainly based on self-reports, which may inevitably cause information bias, though strict quality control measures were implemented in both studies.

## Conclusions

In this large-scale cohort-based analysis, we confirmed the associations between low SES and burdens of overall infectious diseases. We found that SES may impact overall incident infections risk not only by affecting individuals’ lifestyle, environmental pollution and chronic comorbidities directly, but also by distorting the effects of lifestyle and environmental pollution indirectly. In addition, males and non-white people could be more vulnerable to the adverse effect of low SES. Our findings highlighted the importance of improving infections prevention and control in people with low SES. Efforts to enhance health education, such as to encourage smoking cessation and maintaining a healthy diet, and improve the quality of living environment may help reduce burden of infectious disease, especially for people with low SES.

## Supplementary Information

**Additional file 1: Table S1.** Definitions of socioeconomic status, environment pollution, healthy lifestyle, environment pollution and chronic comorbidity factors, and infectious diseases from UK biobank. **Table S2.** Definitions of socioeconomic status, healthy lifestyle, environment pollution and chronic comorbidity factors, and infectious diseases from and US NHANES. **Table S3.** Mean posterior probabilities, prevalence of latent classes, and item-response probabilities in models with three to five latent classes in the UK Biobank and US NHANES. **Table S4.** Associations of SES, lifestyle, environment pollution and chronic comorbidity factors with respiratory, digestive and blood or sexually transmitted infectious diseases in UK Biobank participants under multivariate linear regression. **Table S5.** Associations of SES, lifestyle, environment pollution and chronic comorbidity factors with respiratory infectious diseases in UK Biobank PSM matching subgroup under univariate linear regression. **Table S6.** Associations of SES, lifestyle, environment pollution and chronic comorbidity factors with digestive infectious diseases in UK Biobank PSM matching subgroup under univariate linear regression. **Table S7.** Associations of SES, lifestyle, environment pollution and chronic comorbidity factors with blood or sexually transmitted infectious diseases in UK Biobank PSM matching subgroup under univariate linear regression. **Table S8.** Associations of SES, lifestyle and environment pollution factors with infectious diseases in UK Biobank participants in 2010 under univariate linear regression. **Table S9.** Adjusted odds ratios for environmental pollution on infection in each quantile in the UK Biobank study. **Table S10.** Mediation effects of socioeconomic factors on infectious diseases by individual lifestyle, environment pollution or chronic comorbidity factors in UK Biobank. **Table S11.** Interaction between SES and individual lifestyle, environment pollution or chronic comorbidity factors on infectious diseases in UK Biobank. **Table S12.** Lifestyle scores on infectious diseases in different SES subgroups from UK biobank. **Table S13.** Individual lifestyle factors, and environment pollution and chronic comorbidity factors on infectious diseases in different SES subgroups from UK biobank. **Table S14.** EPS on infectious diseases in different SES subgroups from UK biobank. **Table S15.** APS on infectious diseases in different SES subgroups from UK biobank. **Table S16.** SES on infection in different gender and ethnic/race subgroups in UK Biobank. **Table S17.** Sex and race on infectious diseases in different SES subgroups from UK Biobank. **Table S18.** Baseline characteristics and infection status of all participants from US NHANES. **Table S19.** Associations of SES, lifestyle, environment pollution and chronic comorbidity factors with infectious diseases in US NHANES participants under multivariate linear regression. **Table S20.** Mediation effects of socioeconomic factors on infectious diseases by individual lifestyle, environment pollution or chronic comorbidity factors in US NHANES. **Table S21.** Interaction between SES and lifestyle or chronic comorbidity factors on infectious diseases in US NHANES. **Table S22.** Lifestyle scores on infectious diseases in different SES subgroups from US NHANES. **Table S23.** Individual lifestyle factors, and chronic comorbidity factors on infectious diseases in different SES subgroups from US NHANES. **Table S24.** SES on infection in different gender and ethnic subgroups in US NHANES. **Table S25.** Sex and race on infectious diseases in different SES subgroups from US NHANES. **Table S26.** Distribution of lifestyle factors across ethnic/race subgroups in UK Biobank.

**Additional file 2: Figure S1.** G2 statistics, AIC, and BIC in models with different numbers of latent classes in the UK biobank (a) and US NHANES (b). **Figure S2.** Correlation heatmap of different variables in the UK biobank. **Figure S3.** Bar plots indicating socioeconomic, lifestyle, environment pollution, and chronic comorbidity factors on infection subtypes in matching subgroups from UK biobank. **Figure S4.** Forest plot indicating air pollution score (APS) groups on infectious diseases in different SES subgroups from UK biobank.. **Figure S5.** Forest plot indicating individual environment pollution factors on infectious diseases in different SES subgroups from UK biobank. **Figure S6.** Forest plot indicating lifestyle scores on respiratory infectious diseases in different SES subgroups from UK biobank. **Figure S7.** Forest plot indicating environmental pollution score (EPS) groups on respiratory infectious diseases in different SES subgroups from UK biobank. **Figure S8.** Forest plot indicating air pollution score (APS) groups on respiratory infectious diseases in different SES subgroups from UK biobank. **Figure S9.** Forest plot indicating individual environment pollution factors on respiratory infectious diseases in different SES subgroups from UK biobank. **Figure S10.** Forest plot indicating chronic comorbidity factors on respiratory infectious diseases in different SES subgroups from UK biobank. **Figure S11.** Forest plot indicating lifestyle scores on digestive infectious diseases in different SES subgroups from UK biobank. **Figure S12.** Forest plot indicating environmental pollution score (EPS) groups on digestive infectious diseases in different SES subgroups from UK biobank. **Figure S13.** Forest plot indicating air pollution score (APS) groups on digestive infectious diseases in different SES subgroups from UK biobank. **Figure S14.** Forest plot indicating individual environment pollution factors on digestive infectious diseases in different SES subgroups from UK biobank. **Figure S15.** Forest plot indicating chronic comorbidity factors on digestive infectious diseases in different SES subgroups from UK biobank. **Figure S16.** Forest plot indicating lifestyle scores on blood or sexually transmitted infectious diseases in different SES subgroups from UK biobank. **Figure S17.** Forest plot indicating environmental pollution score (EPS) groups on blood or sexually transmitted infectious diseases in different SES subgroups from UK biobank. **Figure S18.** Forest plot indicating air pollution score (APS) groups on blood or sexually transmitted infectious diseases in different SES subgroups from UK biobank. **Figure S19.** Forest plot indicating individual environment pollution factors on blood or sexually transmitted infectious diseases in different SES subgroups from UK biobank. **Figure S20.** Forest plot indicating chronic comorbidity factors on blood or sexually transmitted infectious diseases in different SES subgroups from UK biobank. **Figure S21.** Forest plot indicating lifestyle scores on infectious diseases in 2010 in different SES subgroups from UK biobank. **Figure S22.** Forest plot indicating environmental pollution score (EPS) groups on infectious diseases in 2010 in different SES subgroups from UK biobank. **Figure S23.** Forest plot indicating air pollution score (APS) groups on infectious diseases in 2010 in different SES subgroups from UK biobank. **Figure S24.** Forest plot indicating individual environment pollution factors on infectious diseases in 2010 in different SES subgroups from UK biobank. **Figure S25.** Forest plot indicating chronic comorbidity factors on infectious diseases in 2010 in different SES subgroups from UK biobank. **Figure S26.** Forest plot indicating lifestyle scores on infectious diseases in different SES subgroups from US NHANES. **Figure S27.** Forest plot indicating individual lifestyle factors and chronic comorbidity factors on infectious diseases in different SES subgroups from US NHANES. **Figure S28.** Forest plot indicating SES on infectious diseases in different sex (a) and ethnic (b) subgroups from US NHANES

## Data Availability

This study has been conducted using UK Biobank resource under Application Number 67665 (http://www.ukbiobank.ac.uk). UK Biobank was established by the Wellcome Trust medical charity, Medical Research Council, Department of Health, Scottish Government, and the Northwest Regional Development Agency. It has also had funding from the Welsh Assembly Government, British Heart Foundation, and Diabetes UK. Publicly available data of NHANES, as well as details on the study design and data collection, can be obtained from the official website (https://www.cdc.gov/nchs/nhanes). All code related to the analyses of the manuscript is available on github repository (https://github.com/XiangyuYe/Infection_SES).

## Abbreviations

SES: Socioeconomic status
CAD: Cardiovascular diseases
UKB: UK Biobank
US NHANES: US National Health and Nutrition Examination Survey
PIR: Poverty to income ratio
HHS: Health and Human Services
LCA: Latent class analysis
AIC: Akaike information criterion
BIC: Bayesian information criterion
TDI: Townsend deprivation index
MEC: Mobile examination center
MET: Metabolic equivalent
HEI: Healthy eating index
NCI: National Cancer Institute
USDA: US Department of Agriculture
FPED: Food Patterns Equivalents Database
PM_2.5_: Particulate matter ≤ 2.5 μm
PM_2.5–10_: Particulate matter 2.5–10 μm
PM_10_: Particulate matter ≤ 10 μm
NO_x_: Nitrogen oxides
NO_2_: Nitrogen dioxide
LUR: Land Use Regression
ESCAPE: European Study of Cohorts for Air Pollution Effects
EPS: Environment pollution score
APS: Air pollution score
CVD: Cardiovascular disease
ICD: International Classification of Diseases
AF: Atrial fibrillation
HBV: Hepatitis B virus
HIV: Human immunodeficiency virus
HSV-2: Herpes simplex virus type 2
HPV: Human papillomavirus
*OR*: Odds ratio
*CI*: Confidence interval
AFR: African
ASA: Asian
nSES: Neighborhood socioeconomic status
SO_2_: Sulfur dioxide
O_3_: Ground-level ozone
EUR: European
