# Supplementary material for: Associations of socioeconomic status with infectious diseases mediated by lifestyle, environmental pollution and chronic comorbidities: a comprehensive evaluation based on UK Biobank
Source: Infect Dis Poverty. 2023 Jan 30;12:5. doi: 10.1186/s40249-023-01056-5 (PMC9885698; doi:10.1186/s40249-023-01056-5)
Supplement: Supplementary file 2 — Additional file 2: Figure S1. G2 statistics, AIC, and BIC in models with different numbers of latent classes in the UK biobank (a) and US NHANES (b). Figure S2. Correlation heatmap of different variables in the UK biobank. Figure S3. Bar plots indicating socioeconomic, lifestyle, environment pollution, and chronic comorbidity factors on infection subtypes in matching subgroups from UK biobank. Figure S4. Forest plot indicating air pollution score (APS) groups on infectious diseases in different SES subgroups from UK biobank.. Figure S5. Forest plot indicating individual environment pollution factors on infectious diseases in different SES subgroups from UK biobank. Figure S6. Forest plot indicating lifestyle scores on respiratory infectious diseases in different SES subgroups from UK biobank. Figure S7. Forest plot indicating environmental pollution score (EPS) groups on respiratory infectious diseases in different SES subgroups from UK biobank. Figure S8. Forest plot indicating air pollution score (APS) groups on respiratory infectious diseases in different SES subgroups from UK biobank. Figure S9. Forest plot indicating individual environment pollution factors on respiratory infectious diseases in different SES subgroups from UK biobank. Figure S10. Forest plot indicating chronic comorbidity factors on respiratory infectious diseases in different SES subgroups from UK biobank. Figure S11. Forest plot indicating lifestyle scores on digestive infectious diseases in different SES subgroups from UK biobank. Figure S12. Forest plot indicating environmental pollution score (EPS) groups on digestive infectious diseases in different SES subgroups from UK biobank. Figure S13. Forest plot indicating air pollution score (APS) groups on digestive infectious diseases in different SES subgroups from UK biobank. Figure S14. Forest plot indicating individual environment pollution factors on digestive infectious diseases in different SES subgroups from UK biobank. Figure S15. Fore [file 40249_2023_1056_MOESM2_ESM.docx]

**Additional File 2 for:**

**Associations of** **socioeconomic status with infectious diseases mediated by lifestyle, environmental pollution and chronic comorbidities: a comprehensive evaluation based on UK Biobank**

Xiangyu Ye^1#^, Yidi Wang^1#^, Yixin Zou^1^, Junlan Tu^1^, Weiming Tang^1,2^, Rongbin Yu^1^, Sheng Yang^3*^, Peng Huang^1*^

^1^ Department of Epidemiology, Center for Global Health, School of Public Health, Nanjing Medical University, Nanjing, China

^2^ Institute of Global Health and Infectious Diseases, University of North Carolina, Chapel Hill, CA, USA.

^3^ Department of Biostatistics, Center for Global Health, School of Public Health, Nanjing Medical University, Nanjing, China

^#^ Xiangyu Ye and Yidi Wang contributed equally to this work.

Corresponding authors (*): Peng Huang: [huangpeng@njmu.edu.cn](mailto:huangpeng@njmu.edu.cn), Sheng Yang: [yangsheng@njmu.edu.cn](mailto:yangsheng@njmu.edu.cn)

**Supplementary methods**

**Study population for US NHANES**

US NHANES is a program designed to assess the health and nutritional status of adults and children in US. [1] The survey used a complex, multistage, probability sampling design to select participants representative of the civilian, non-institutionalized US population. Continuous data collection began in 1999 with public data released in a 2-year cycle on about 10,000 participants. [2] We included 101,316 participants surveyed from 1999 to 2018, and followed Zhang et al. to remove individuals: (i) who were less than 20 years old; (ii) who were pregnant; (iii) who had missing information on socioeconomic factors or other covariates; (iv) who had non-positive sample weights for an interview or health examination in the datasets. [3] Finally, we retained 45,671 participants in US NHANES for subsequent analysis (**Fig.1b**).

**Assessment of socioeconomic status**

We used all four variables, including family income level, education qualification, employment status, and health insurance coverage, to assess the individual level SES in US NHANES (**Additional File 1: Table S2**). Family income level was assessed using family poverty to income ratio (PIR), which reﬂected the family income relative to the federal poverty level and was calculated by dividing family income by the poverty guidelines released by the Department of Health and Human Services (HHS) annually. [4] PIR was obtained through the family questionnaire in US NHANES, and was recorded as continuous values with higher values indicating a higher income level. Following Odutayo et al., [5] we to regrouped it into three levels: (i) ≤ 1; (ii) 1–4; and (iii) ≥4. Education, employment status, and health insurance were obtained through a survey participant questionnaire. Education qualification was recorded as seven levels: (i) Less Than 9th Grade; (ii) 9–11th Grade (Includes 12th grade with no diploma); (iii) High School Grad/GED or Equivalent; (iv) Some College or AA degree; (v) College Graduate or above; (vi) Refused; and (vii) Don't Know. We simply kept the order recorded but removed the individuals choosing the last option. Employment status was categorized based on two questions: “Type of work done last week” and “Main reason did not work last week”. We still simply regrouped participants into two groups as in UK biobank (UKB): employed (those answered as: (i) Working at a job or business; (ii) With a job or business but not at work; (iii) Not working at a job or business due to going to school or (iv) due to retirement), and unemployed (those answered: (i) Looking for work; or (ii) Not working at a job or business due to other reasons). In terms of health insurance, participants were surveyed on insurance coverage, and types of insurance coverage. We classified them into three groups: (i) private health insurance coverage (including any private health insurance, Medi-Gap, or single-service plan); (ii) public health insurance only; and (iii) no health insurance coverage. [6]

We also used latent class analysis (LCA) implanted in R package *poLCA* (v1.6.0) to estimate SES based on the above four variables with maximum times of iterations and the tolerance value for judging convergence set 10,000 and 1×10^-6^, respectively. [3, 7] We fitted the different LCA model with 2–10 latent classes to select a reasonable latent class number, and models failed to converge when the class number is greater than six, respectively. Based on Akaike information criterion (AIC), Bayesian information criterion (BIC), likelihood ratio statistic (*G^2^*), and mean posterior probability, three latent classes were identified, which respectively represented a high, medium, and low SES according to the item-response probabilities (**Additional File 1: Table S3** and **Additional File 2: Figure S1**).

**Assessment of lifestyle factors**

Information on lifestyle factors in US NHANES, including smoking status, physical activities and sleep patterns information, were obtained through SP questionnaire, alcohol consumption and drug use information through mobile examination center (MEC) questionnaire, and diet information through 24-hour dietary recall. [8] Certain differences in the survey process and data structure exist between UKB and US NHANES, and we also tried to define healthy levels in US NHANES as in UKB for harmonization (**Additional File 1: Table S2**). Specifically, “No current smoking” was defined as smoking less than 100 cigarettes in life or had quit for more than 30 years. “No alcohol consumption” was defined as having less than 12 alcohol drinks in life. Limited information on sleep habits and disorders was recorded in US NHANES, and “Healthy sleep pattern” was defined as sleep 7–8 hours per day and no self-reported or informed sleep disorder. Since 2007, participants in US NHANES were asked about time and frequency of doing moderate and vigorous physical activity in leisure time, and those who either engaged in vigorous activity for at least one day and moderate physical activity for at least five days per week from the perspective of frequency, or exercised of vigorous activity for at least 75 minutes or moderate activity for 150 minutes per week from the perspective of time were defined as achieving “Regular physical activity”. However, from 1999 to 2006, details on moderate and vigorous physical activity were not supplied, but metabolic equivalent (MET) score was available alternatively. We thus followed Zhang et al. to defined those with top third MET score as having “Regular physical activity”. [3] Ever and current use of marijuana or hashish, cocaine, heroin, and methamphetamine were recorded in US NHANES, and we defined “No drug use” as never using any type of these drugs. Following Liu et al. and Li et al., [9, 10] we used the updated healthy eating index (HEI-2015), which was released by the US Department of Health and Human Services’ National Cancer Institute (NCI) and the US Department of Agriculture (USDA) and contains 13 components that sum to a total maximum score of 100 points, to assess dietary patterns in US NHANES. Methods for HEI-2015 scores calculation can be found on NCI website, [11] and weights for foods to USDA Food Patterns components conversion were downloaded from the Food Patterns Equivalents Database (FPED). [12] Those with HEI-2015 in the top two fifths of distribution as having “Healthy diet pattern”.

We also assigned 1 point for a healthy level while 0 points for an unhealthy level for each lifestyle factor as in UKB. The lifestyle variable was defined as the summation of the six variables and was divided participants into 3 groups: poor group (0–1 point), medium (2–3 points) and healthy (4–6 points).

**Assessment of chronic disease history**

Information on disease history was obtained through SP and MEC questionnaires in US NHANES. We totally defined 4199 (weighted proportion of 27.7%) participants who had previously been diagnosed with hypertension as having a history of CVD. We also defined 1322 (7.0% weighted) participants with the history of diabetes, and 169 (10.7% weighted) with the history of psychiatric disorders, including 122 (8.0% weighted) with depression, 43 (3.1% weighted) with anxiety, and 44 (2.6% weighted) with panic (**Additional File 1: Table S2**).

**Definition of infectious diseases**

For infectious diseases in US NHANES, laboratory tests for limited types of infectious diseases were conducted in sampled participants, and we only included those that could be used to clearly define current infections instead of immunity, including chlamydia, gonorrhea, hepatitis B virus (HBV), HCV, HDV, HEV, human immunodeficiency virus (HIV), herpes simplex virus type 2 (HSV-2), human papillomavirus (HPV), syphilis, and tuberculosis. We totally defined 10,434 (23.2% weighted) cases with infectious diseases (**Additional File 1: Table S2**).

**Reference**

1. Ahluwalia N, Dwyer J, Terry A, Moshfegh A, Johnson C: **Update on NHANES Dietary Data: Focus on Collection, Release, Analytical Considerations, and Uses to Inform Public Policy**. *Adv Nutr* 2016, **7**(1):121-134.

2. **Health and nutrition examination survey plan and operations, 1999-2010**. In*.* Edited by National Center for Health S, vol. 56. Hyattsville, MD; 2013.

3. Zhang Y-B, Chen C, Pan X-F, Guo J, Li Y, Franco OH *et al*: **Associations of healthy lifestyle and socioeconomic status with mortality and incident cardiovascular disease: two prospective cohort studies**. *BMJ* 2021, **373**:n604.

4. Oddo VM, Jones-Smith JC: **Gains in income during early childhood are associated with decreases in BMI z scores among children in the United States**. *Am J Clin Nutr* 2015, **101**(6):1225-1231.

5. Odutayo A, Gill P, Shepherd S, Akingbade A, Hopewell S, Tennankore K *et al*: **Income Disparities in Absolute Cardiovascular Risk and Cardiovascular Risk Factors in the United States, 1999-2014**. *JAMA Cardiol* 2017, **2**(7):782-790.

6. Le P, Chaitoff A, Misra-Hebert AD, Ye W, Herman WH, Rothberg MB: **Use of Antihyperglycemic Medications in U.S. Adults: An Analysis of the National Health and Nutrition Examination Survey**. *Diabetes Care* 2020, **43**(6):1227-1233.

7. Linzer DA, Lewis JB: **poLCA: An R Package for Polytomous Variable Latent Class Analysis**. *J Stat Softw* 2011, **42**(10):1 - 29.

8. Dwyer J, Picciano MF, Raiten DJ: **Collection of Food and Dietary Supplement Intake Data: What We Eat in America–NHANES**. *J Nutr* 2003, **133**(2):590S-600S.

9. Liu J, Rehm CD, Onopa J, Mozaffarian D: **Trends in Diet Quality Among Youth in the United States, 1999-2016**. *JAMA* 2020, **323**(12):1161-1174.

10. Li Y, Schoufour J, Wang DD, Dhana K, Pan A, Liu X *et al*: **Healthy lifestyle and life expectancy free of cancer, cardiovascular disease, and type 2 diabetes: prospective cohort study**. *BMJ* 2020, **368**:l6669.

11. **HEI Scoring Algorithm** [<https://epi.grants.cancer.gov/hei/hei-scoring-method.html>]. Accessed 20 Aug 2022

12. **FPED databases** [<https://www.ars.usda.gov/northeast-area/beltsville-md-bhnrc/beltsville-human-nutrition-research-center/food-surveys-research-group/docs/fped-databases/>]. Accessed 16 Aug 2022

**Supplementary Figures**


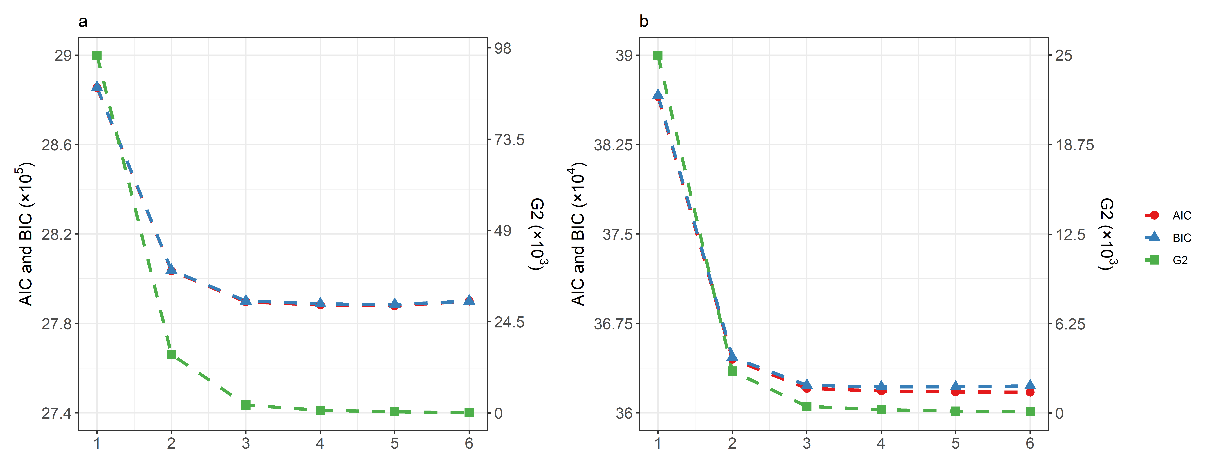


**Figure S1 G2 statistics, AIC, and BIC in models with different numbers of latent classes in the UK biobank (a) and US NHANES (b).** Family income level, occupation, and education level were used to generate an overall SES parameter via latent class analysis. Abbreviations: AIC: Akaike information criterion; BIC: Bayesian information criterion.


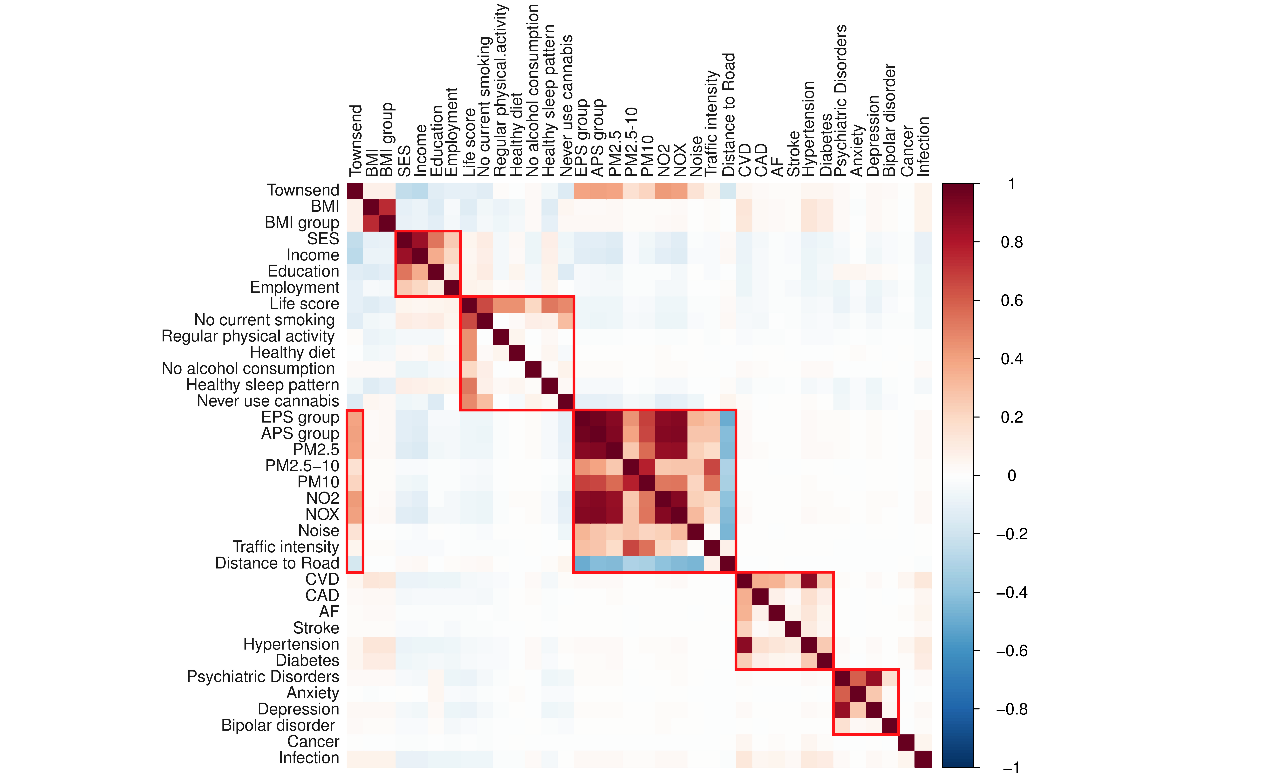


**Figure S2 Correlation heatmap of different variables in the UK biobank.** Colors from red to white, and to blue represent correlation from strong positive to weak correlation, and to strong negative. Note that socioeconomic factors, including SES, income, education, employment, were all coded as from low to high. Abbreviations: BMI: Body mass index; SES: Socioeconomic status; APS: Air pollution score; EPS: Environment pollution score; PM_2.5_: Particulate matter ≤ 2.5 μm; PM_2.5–10_: Particulate matter 2.5–10 μm; PM_10_: Particulate matter ≤ 10 μm; NO_x_: Nitrogen oxides; NO_2_: Nitrogen dioxide; CVD: Cardiovascular disease; CAD: Cardiovascular diseases; AF: Atrial fibrillation.


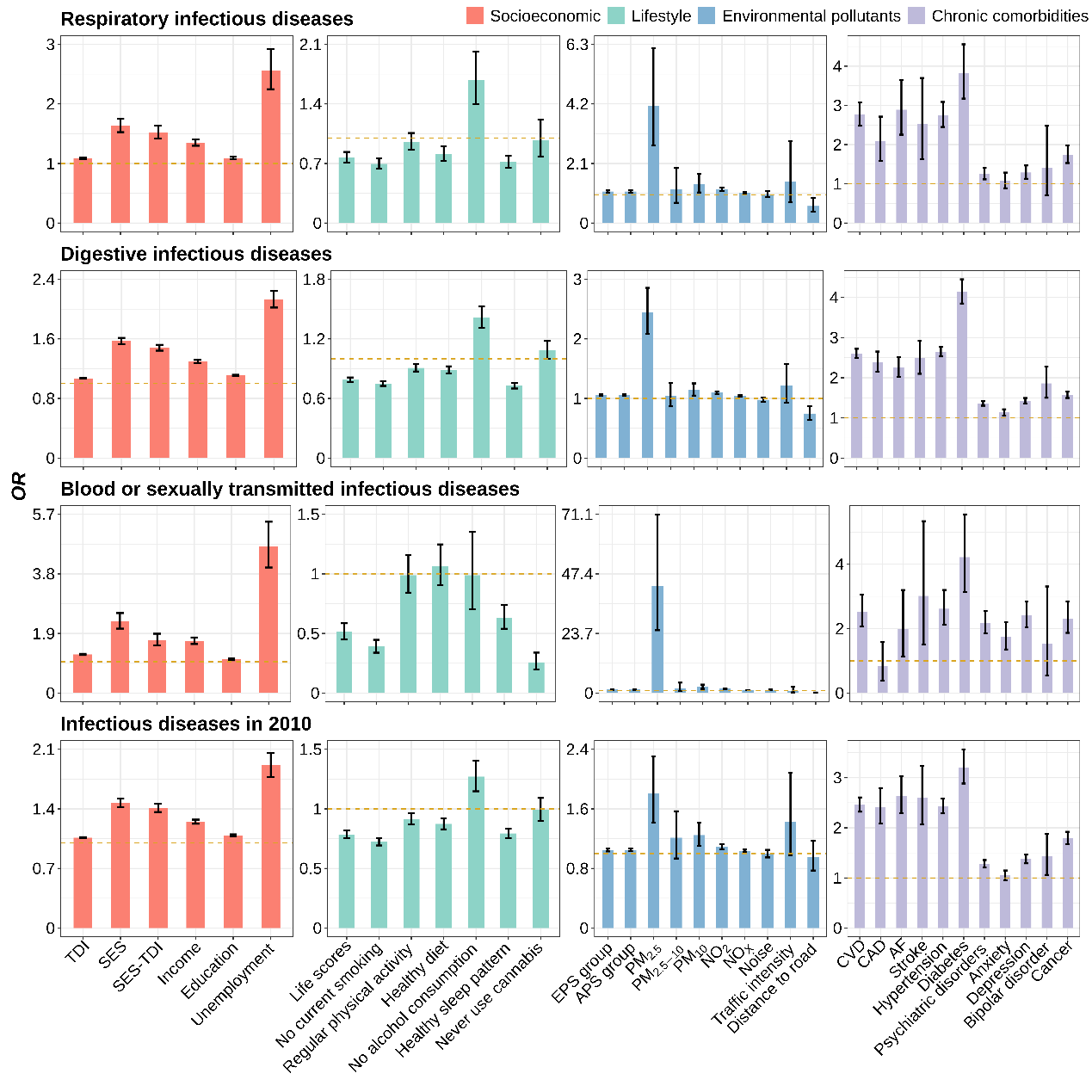


**Figure S3 Bar plots indicating socioeconomic, lifestyle, environment pollution, and chronic comorbidity factors on infection subtypes in matching subgroups from UK biobank.** Odds ratios (*ORs*) were adjusted for age, sex, ethnic and assessment center. Dashed line represents no significant association. Abbreviations: TDI: Townsend deprivation; SES: Socioeconomic status; APS: Air pollution score; EPS: Environment pollution score; PM_2.5_: Particulate matter ≤ 2.5 μm; PM_2.5–10_: Particulate matter 2.5–10 μm; PM_10_: Particulate matter ≤ 10 μm; NO_x_: Nitrogen oxides; NO_2_: Nitrogen dioxide; CVD: Cardiovascular disease; CAD: Cardiovascular diseases; AF: Atrial fibrillation.


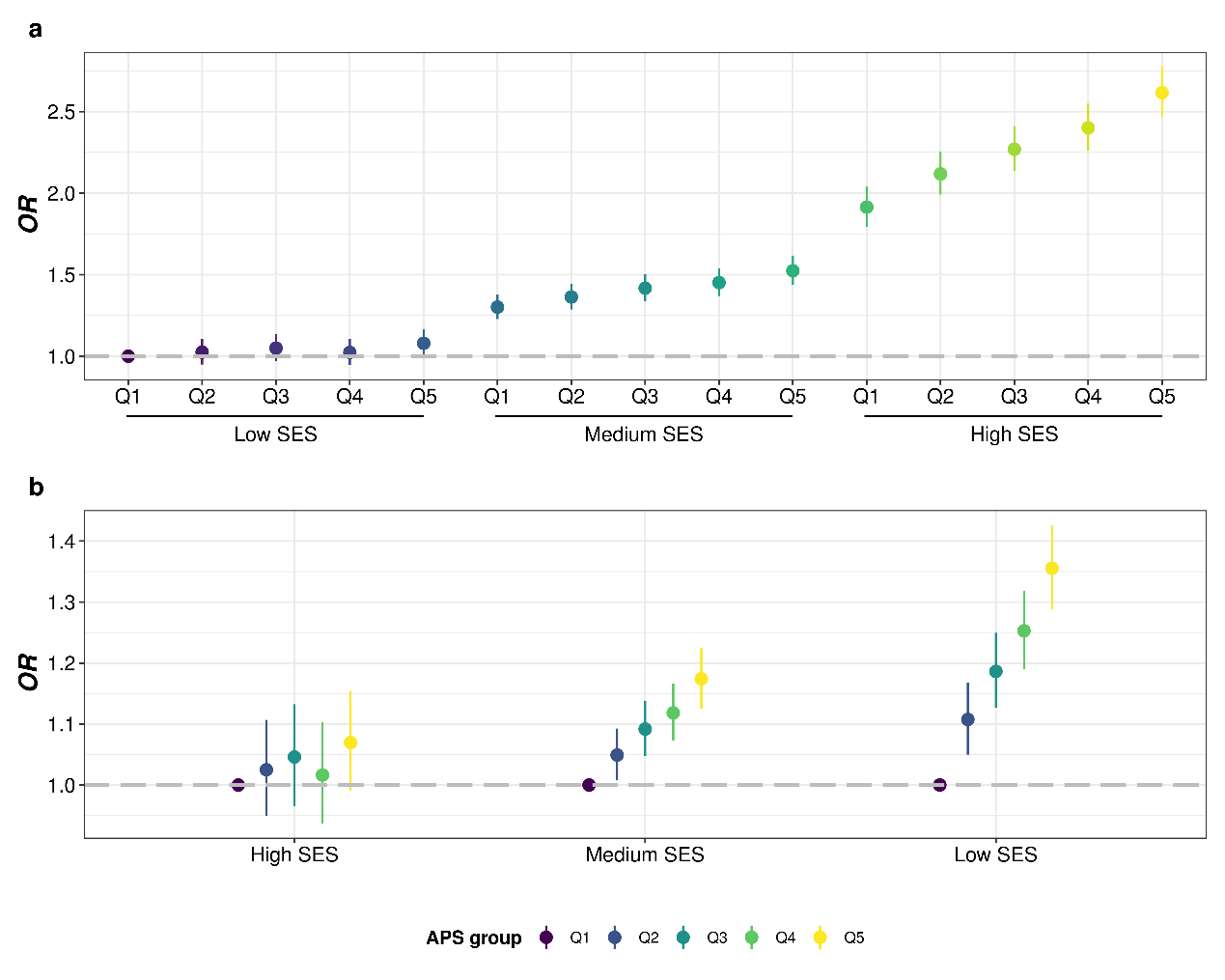


**Figure S4 Forest plot indicating air pollution score (APS) groups on infectious diseases in different SES subgroups from UK biobank.** The group with high SES and low APS (top fifth, Q1) was selected as the overall control group **(a)**, or for each SES subgroup individually, that with low APS (Q1) was selected as the control group **(b)**. Odds ratios (*ORs*) were adjusted for age, sex, ethnic and assessment center. Dashed line represents no significant association. Abbreviations: SES: Socioeconomic status.


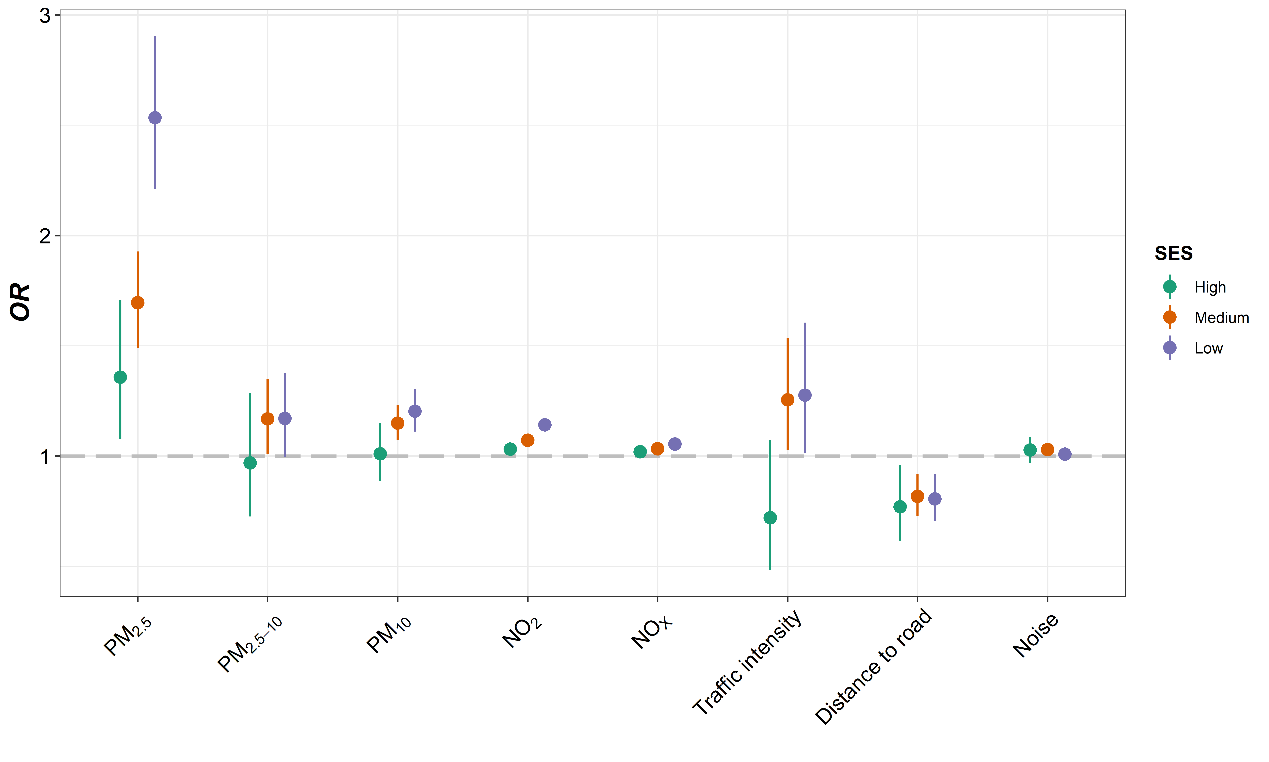


**Figure S5 Forest plot indicating individual environment pollution factors on infectious diseases in different SES subgroups from UK biobank.** Odds ratios (*ORs*) were estimated on per 10-unit increase, and were adjusted for age, sex, ethnic and assessment center. Dashed line represents no significant association. Abbreviations: SES: Socioeconomic status; PM_2.5_: Particulate matter ≤ 2.5 μm; PM_2.5–10_: Particulate matter 2.5–10 μm; PM_10_: Particulate matter ≤ 10 μm; NO_x_: Nitrogen oxides; NO_2_: Nitrogen dioxide.


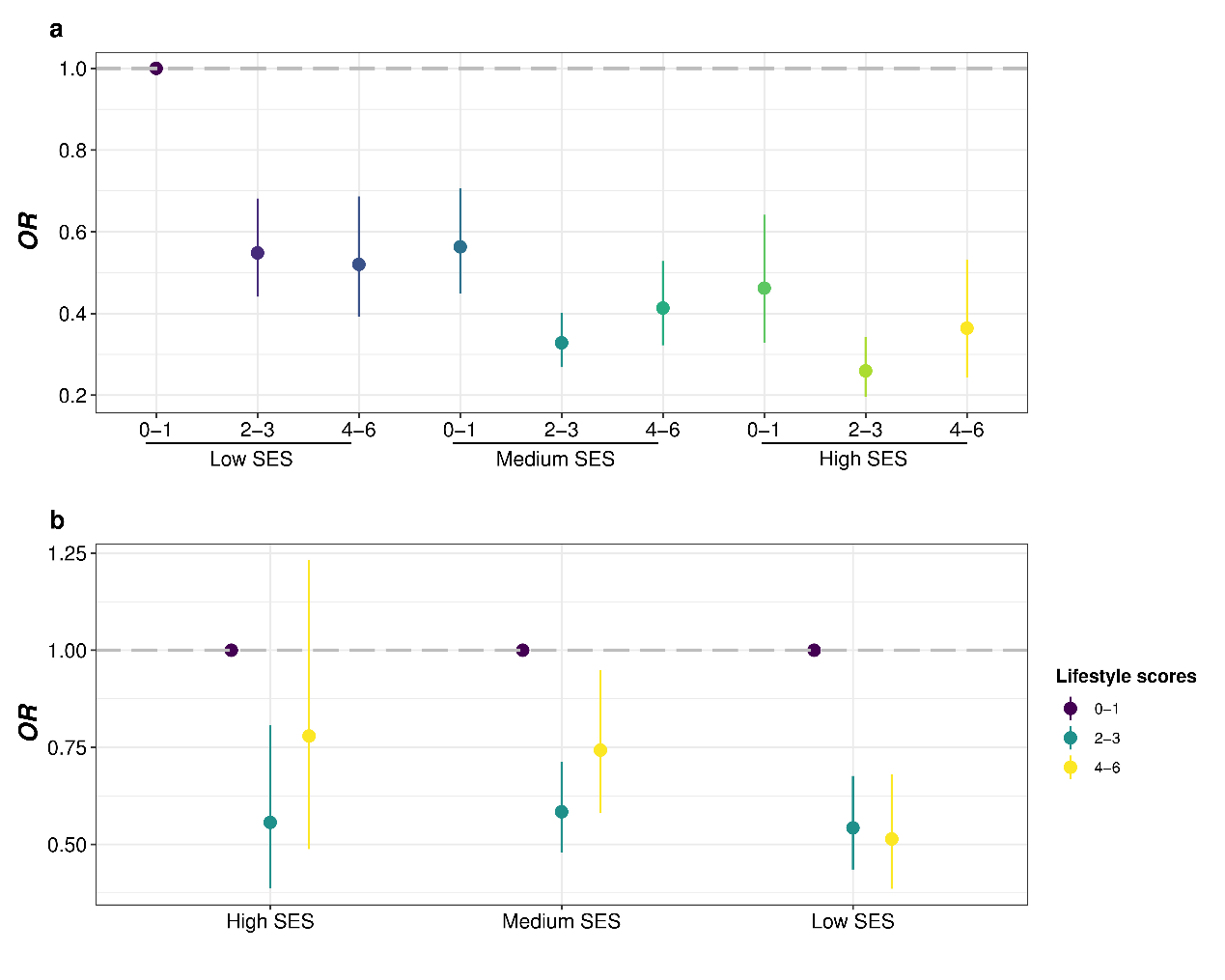


**Figure S6 Forest plot indicating lifestyle scores on respiratory infectious diseases in different SES subgroups from UK biobank.** The group with low SES and poor lifestyle scores (0–1) was selected as the overall control group **(a)**, or for each SES subgroup individually, that with poor lifestyle scores (0–1) was selected as the control group **(b)**. Odds ratios (*ORs*) were adjusted for age, sex, ethnic and assessment center. Dashed line represents no significant association. Abbreviations: SES: Socioeconomic status.


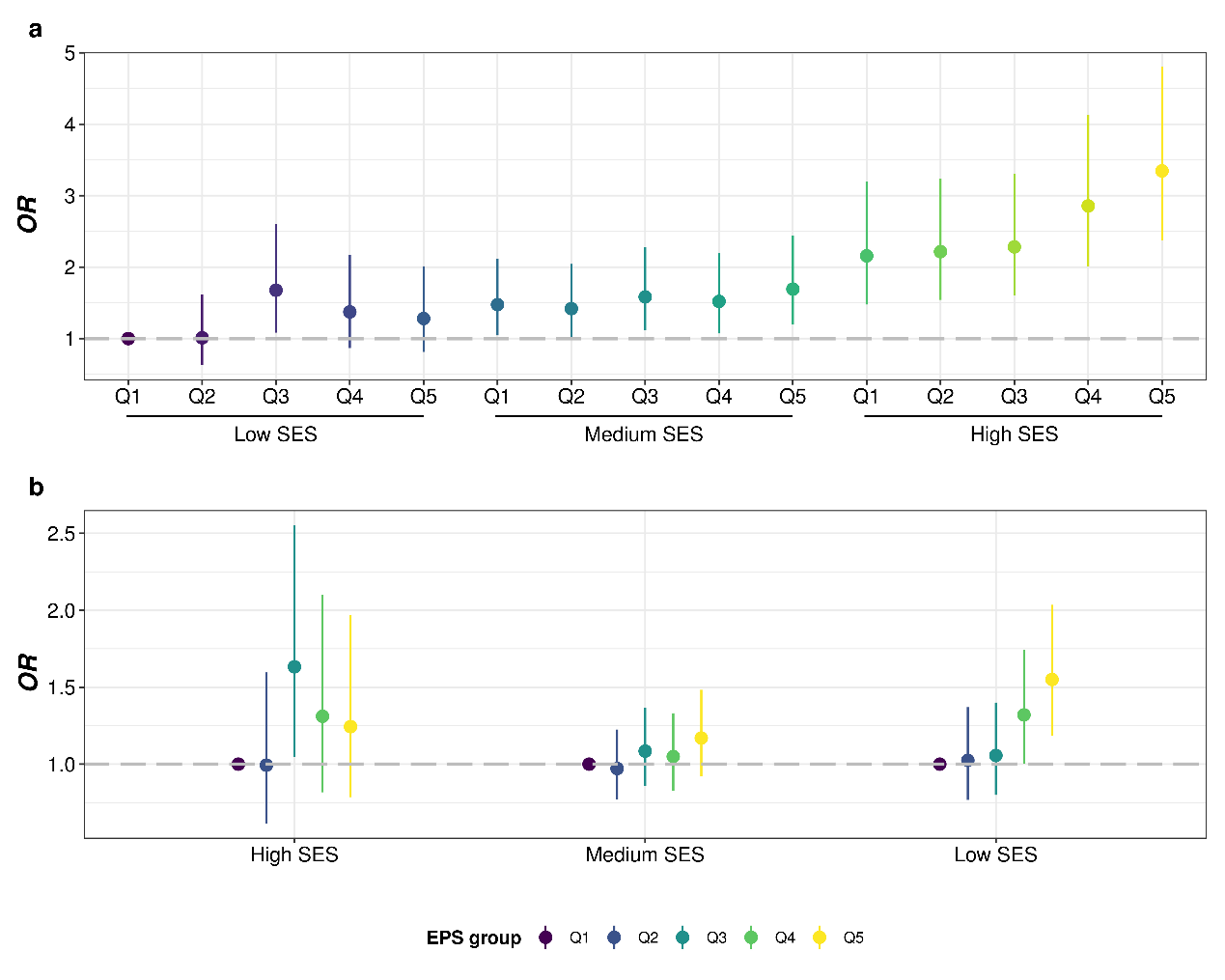


**Figure S7 Forest plot indicating environmental pollution score (EPS) groups on respiratory infectious diseases in different SES subgroups from UK biobank.** The group with high SES and low EPS (top fifth, Q1) was selected as the overall control group **(a)**, or for each SES subgroup individually, that with low EPS (Q1) was selected as the control group **(b)**. Odds ratios (*ORs*) were adjusted for age, sex, ethnic and assessment center. Dashed line represents no significant association. Abbreviations: SES: Socioeconomic status.


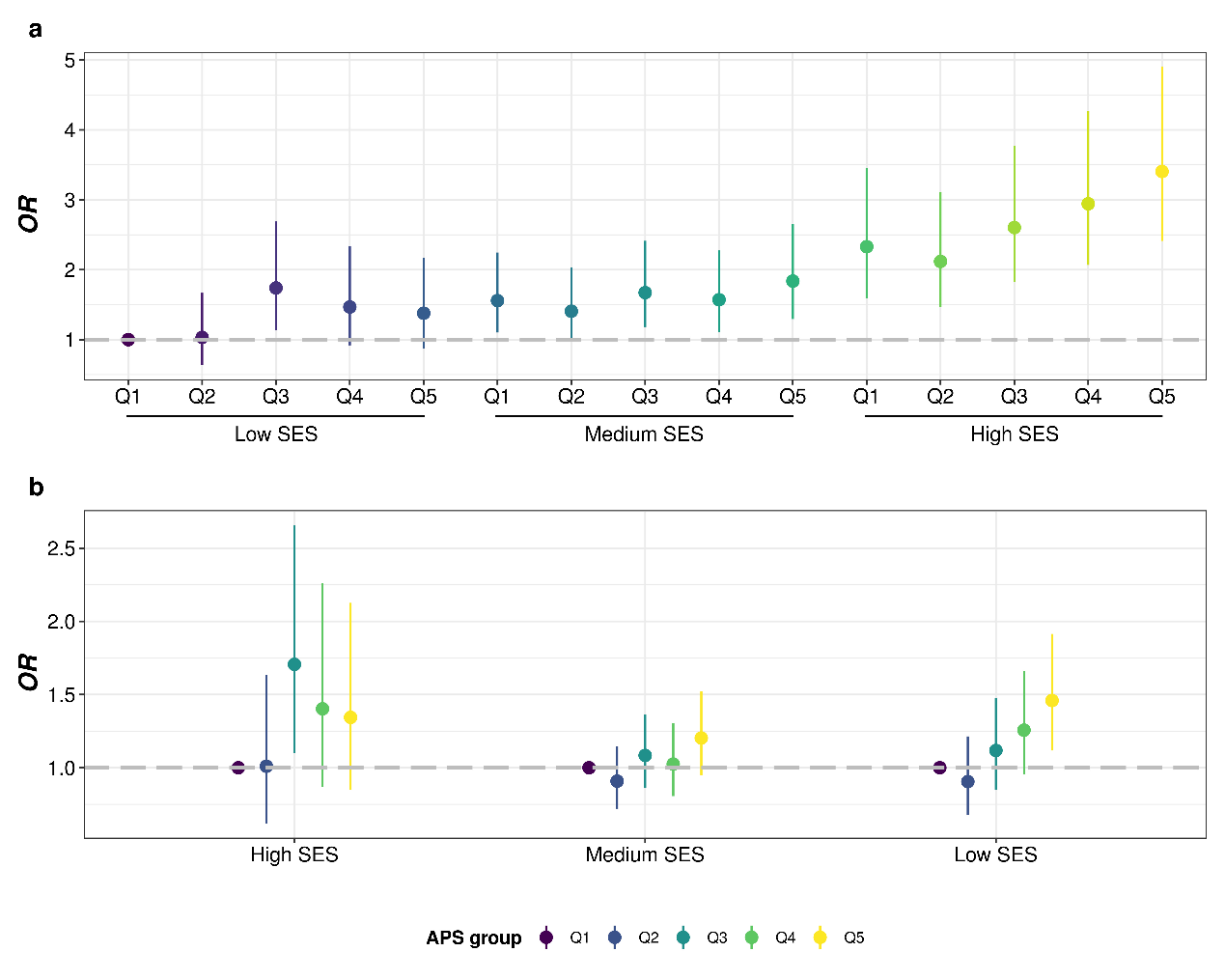


**Figure S8 Forest plot indicating air pollution score (APS) groups on respiratory infectious diseases in different SES subgroups from UK biobank.** The group with high SES and low APS (top fifth, Q1) was selected as the overall control group **(a)**, or for each SES subgroup individually, that with low APS (Q1) was selected as the control group **(b)**. Odds ratios (*ORs*) were adjusted for age, sex, ethnic and assessment center. Dashed line represents no significant association. Abbreviations: SES: Socioeconomic status.


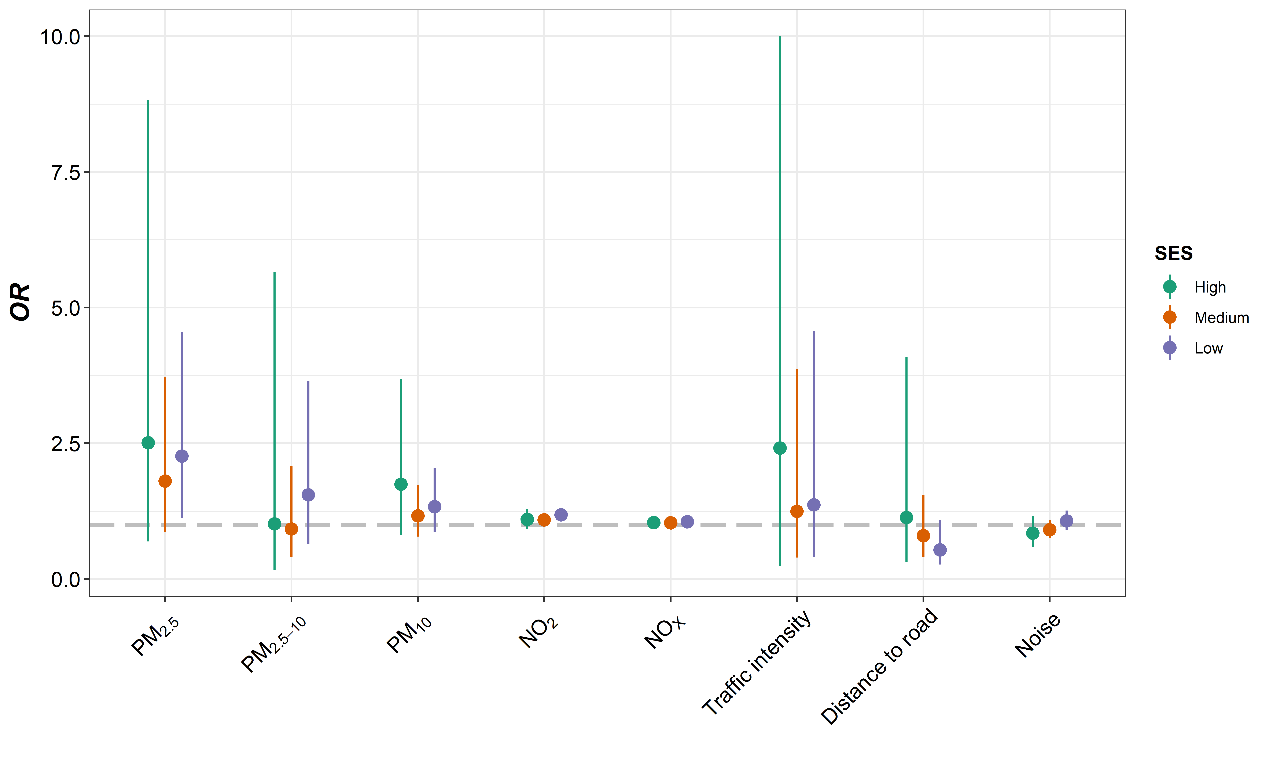


**Figure S9 Forest plot indicating individual environment pollution factors on respiratory infectious diseases in different SES subgroups from UK biobank.** Odds ratios (*ORs*) were estimated on per 10-unit increase, and were adjusted for age, sex, ethnic and assessment center. Confidence intervals (*CIs*) are truncated at 10. Dashed line represents no significant association. Abbreviations: PM_2.5_: Particulate matter ≤ 2.5 μm; PM_2.5–10_: Particulate matter 2.5–10 μm; PM_10_: Particulate matter ≤ 10 μm; NO_x_: Nitrogen oxides; NO_2_: Nitrogen dioxide.


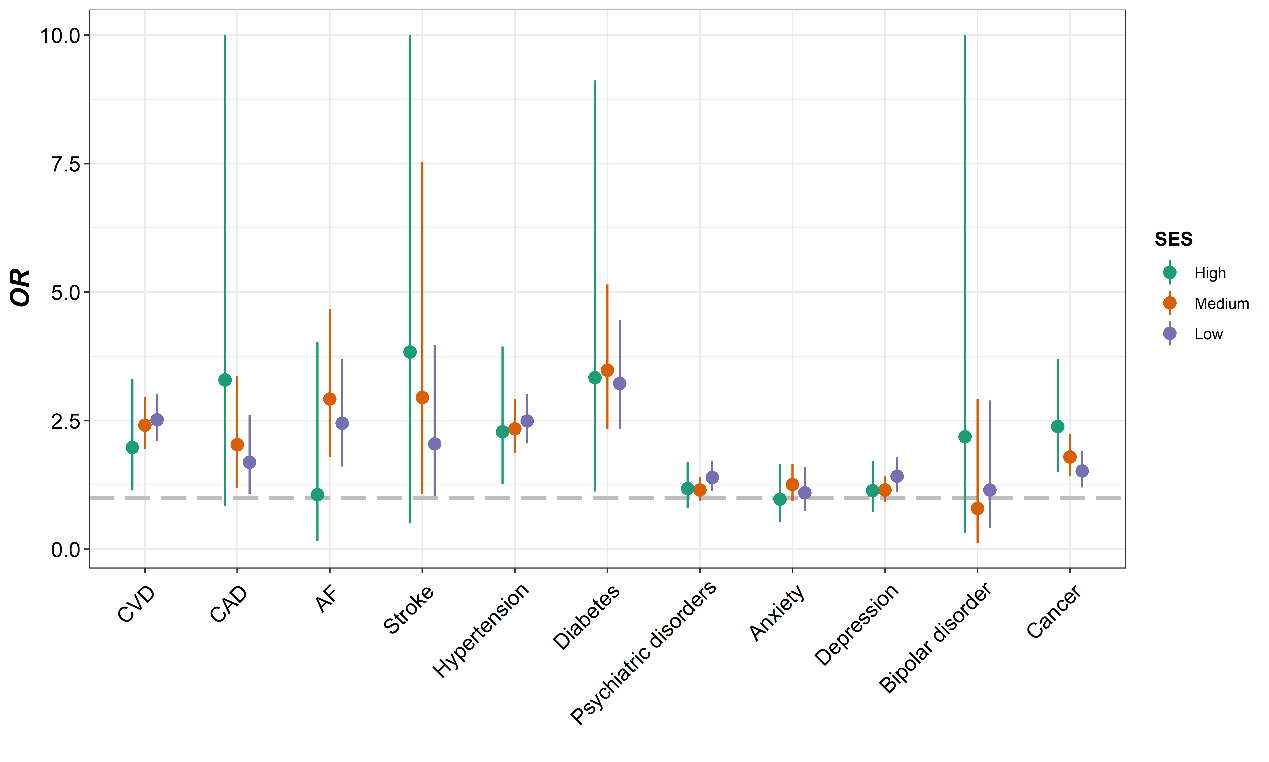


**Figure S10 Forest plot indicating chronic comorbidity factors on respiratory infectious diseases in different SES subgroups from UK biobank.** Odds ratios (*ORs*) were adjusted for age, sex, ethnic and assessment center. Confidence intervals (*CIs*) are truncated at 10. Dashed line represents no significant association. Abbreviations: CVD: Cardiovascular disease; CAD: Cardiovascular diseases; AF: Atrial fibrillation.


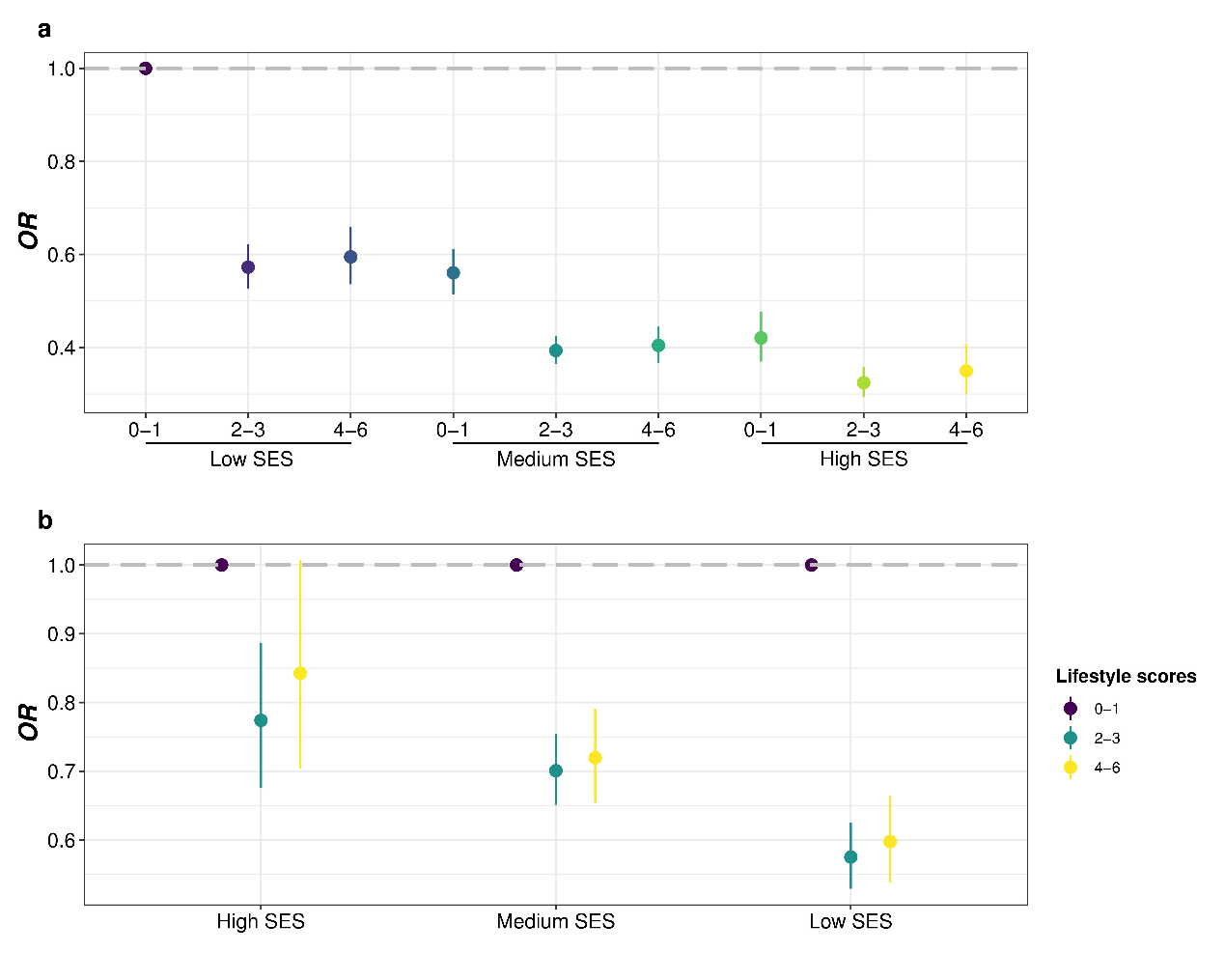


**Figure S11 Forest plot indicating lifestyle scores on digestive infectious diseases in different SES subgroups from UK biobank.** The group with low SES and poor lifestyle scores (0–1) was selected as the overall control group **(a)**, or for each SES subgroup individually, that with poor lifestyle scores (0–1) was selected as the control group **(b)**. Odds ratios (*ORs*) were adjusted for age, sex, ethnic and assessment center. Dashed line represents no significant association. Abbreviations: SES: Socioeconomic status.


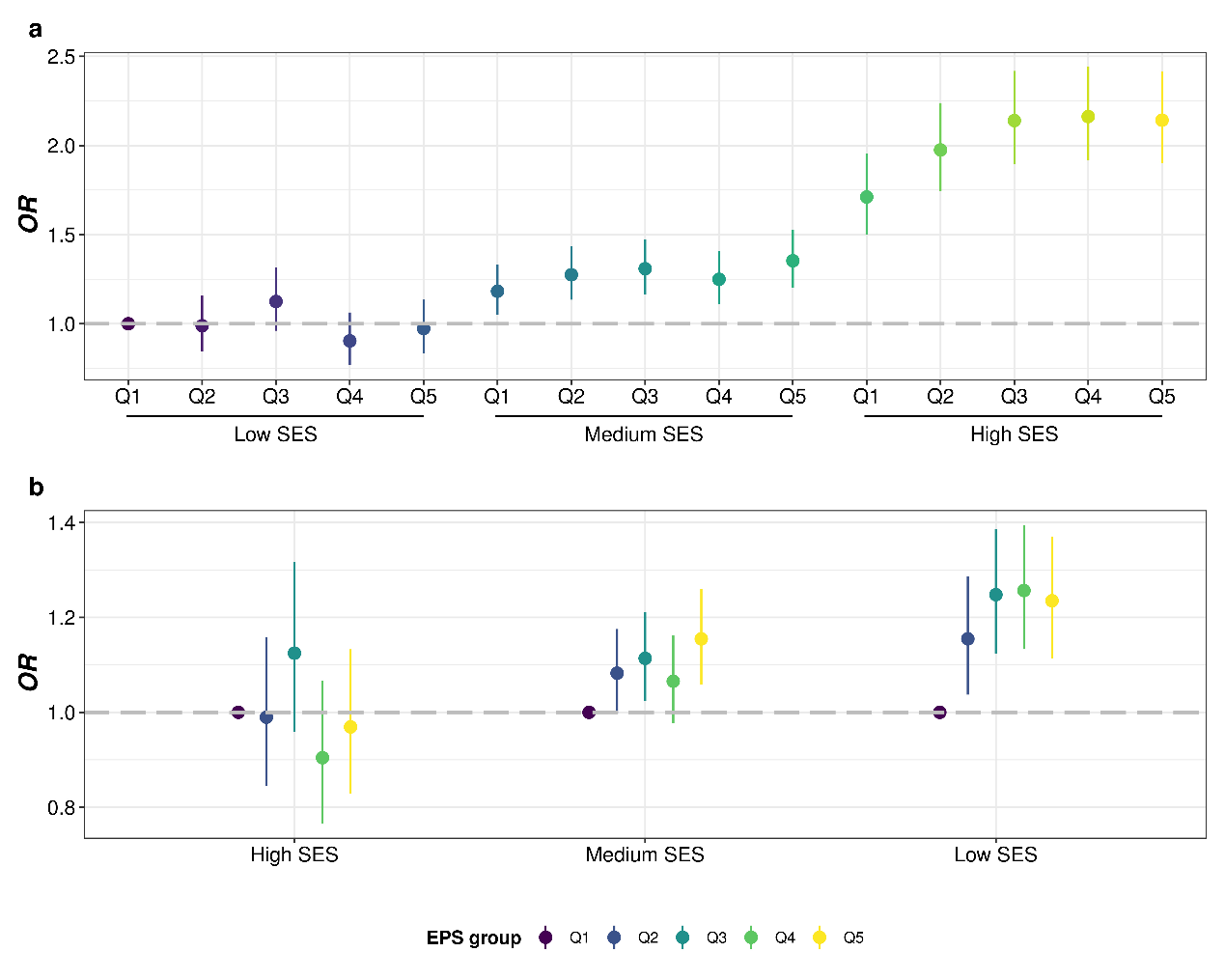


**Figure S12 Forest plot indicating environmental pollution score (EPS) groups on digestive infectious diseases in different SES subgroups from UK biobank.** The group with high SES and low EPS (top fifth, Q1) was selected as the overall control group **(a)**, or for each SES subgroup individually, that with low EPS (Q1) was selected as the control group **(b)**. Odds ratios (*ORs*) were adjusted for age, sex, ethnic and assessment center. Dashed line represents no significant association. Abbreviations: SES: Socioeconomic status.


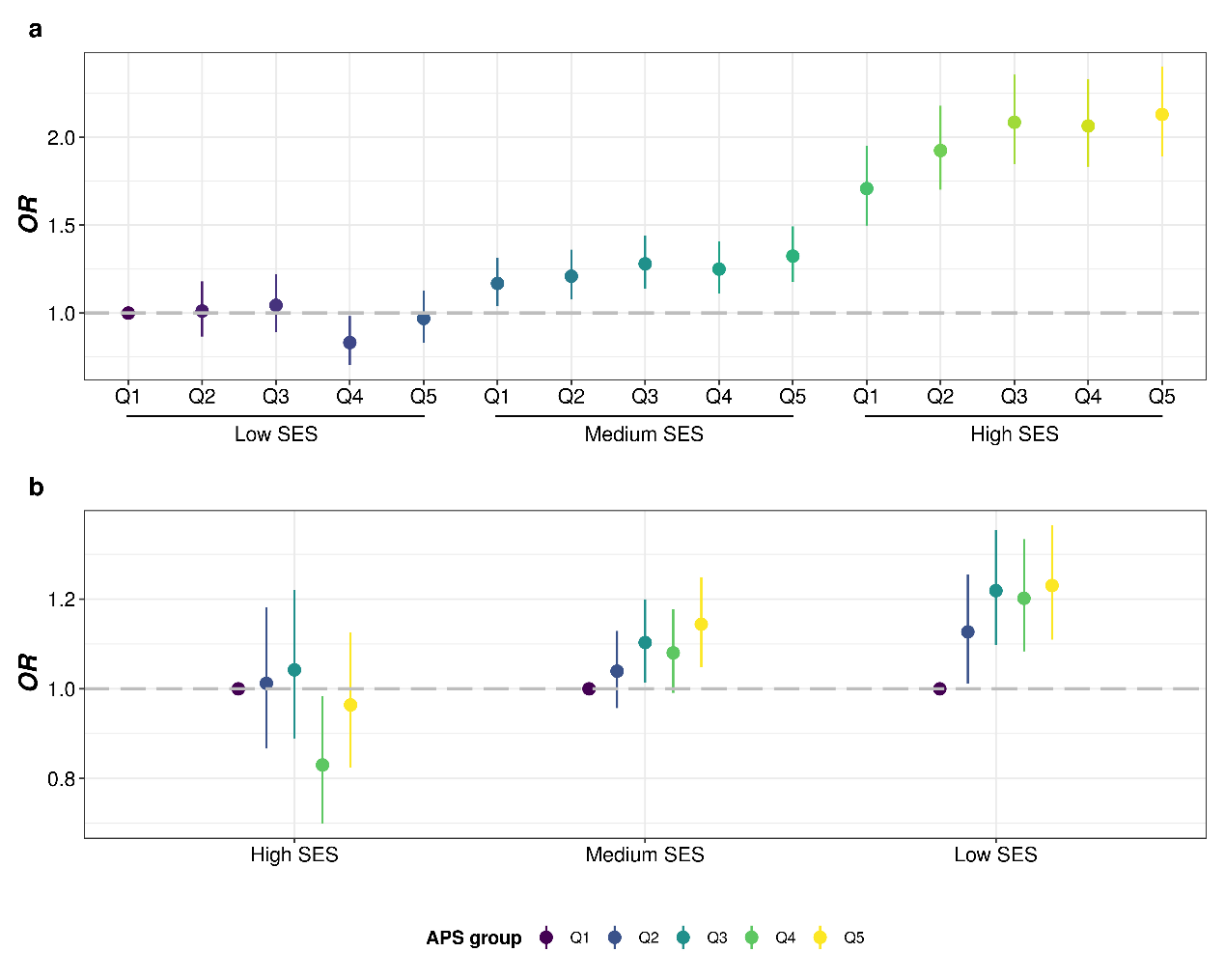


**Figure S13 Forest plot indicating air pollution score (APS) groups on digestive infectious diseases in different SES subgroups from UK biobank.** The group with high SES and low EPS (top fifth, Q1) was selected as the overall control group **(a)**, or for each SES subgroup individually, that with low EPS (Q1) was selected as the control group **(b)**. Odds ratios (*ORs*) were adjusted for age, sex, ethnic and assessment center. Dashed line represents no significant association. Abbreviations: SES: Socioeconomic status.


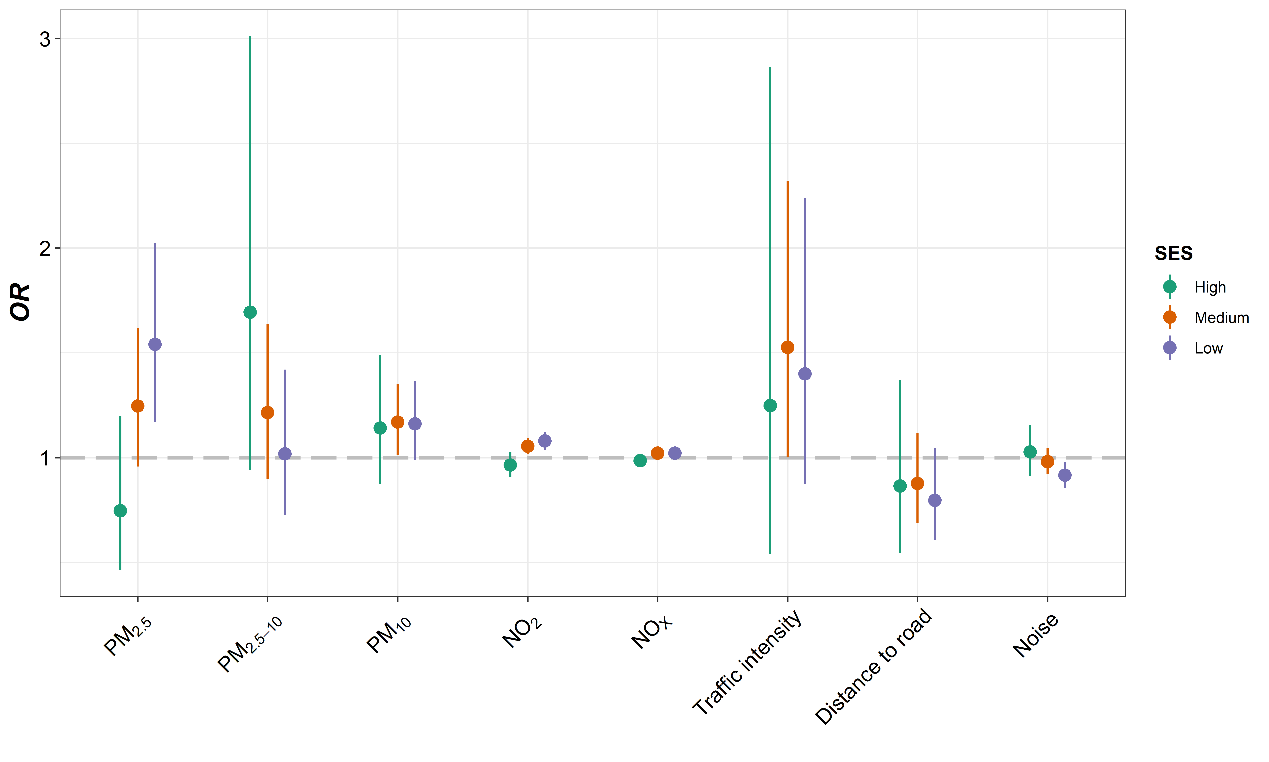


**Figure S14 Forest plot indicating individual environment pollution factors on digestive infectious diseases in different SES subgroups from UK biobank.** Odds ratios (*ORs*) were estimated on per 10-unit increase, and were adjusted for age, sex, ethnic and assessment center. Dashed line represents no significant association. Abbreviations: PM_2.5_: Particulate matter ≤ 2.5 μm; PM_2.5–10_: Particulate matter 2.5–10 μm; PM_10_: Particulate matter ≤ 10 μm; NO_x_: Nitrogen oxides; NO_2_: Nitrogen dioxide.


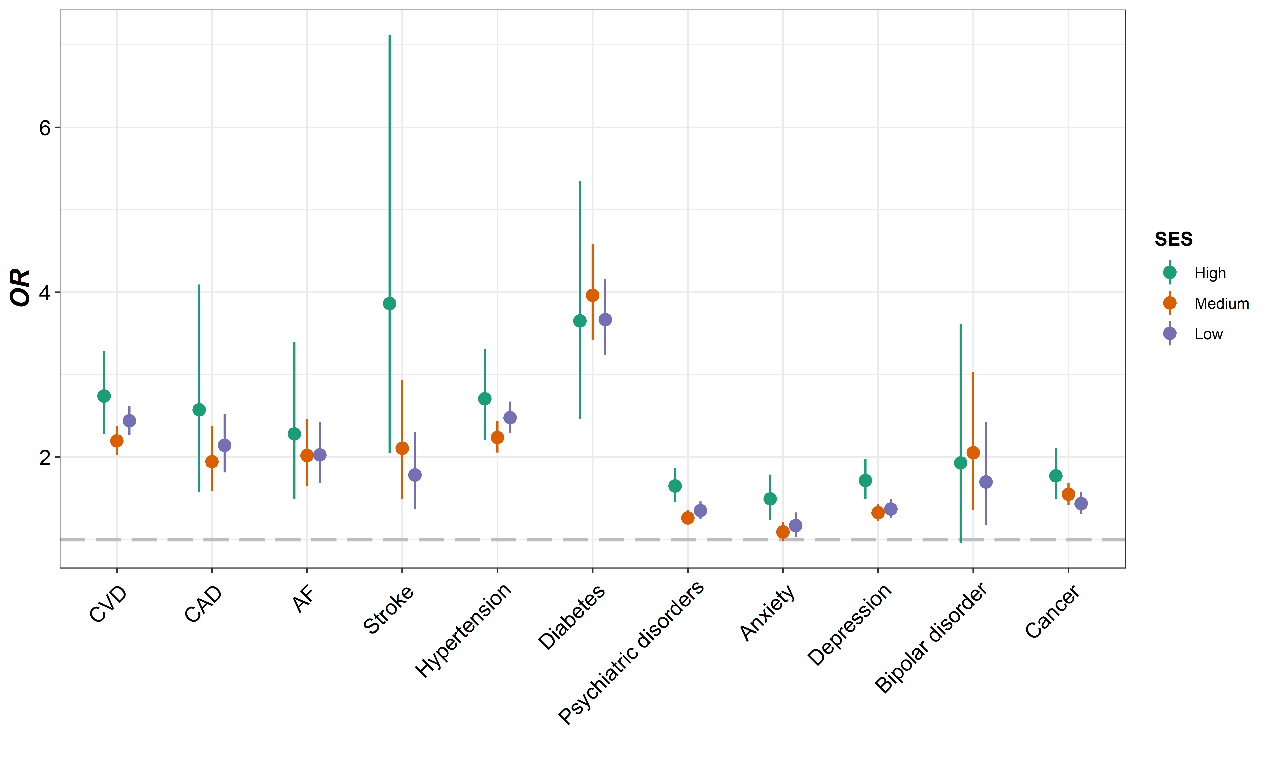


**Figure S15 Forest plot indicating chronic comorbidity factors on digestive infectious diseases in different SES subgroups from UK biobank.** Odds ratios (*ORs*) were adjusted for age, sex, ethnic and assessment center. Dashed line represents no significant association. Abbreviations: CVD: Cardiovascular disease; CAD: Cardiovascular diseases; AF: Atrial fibrillation.


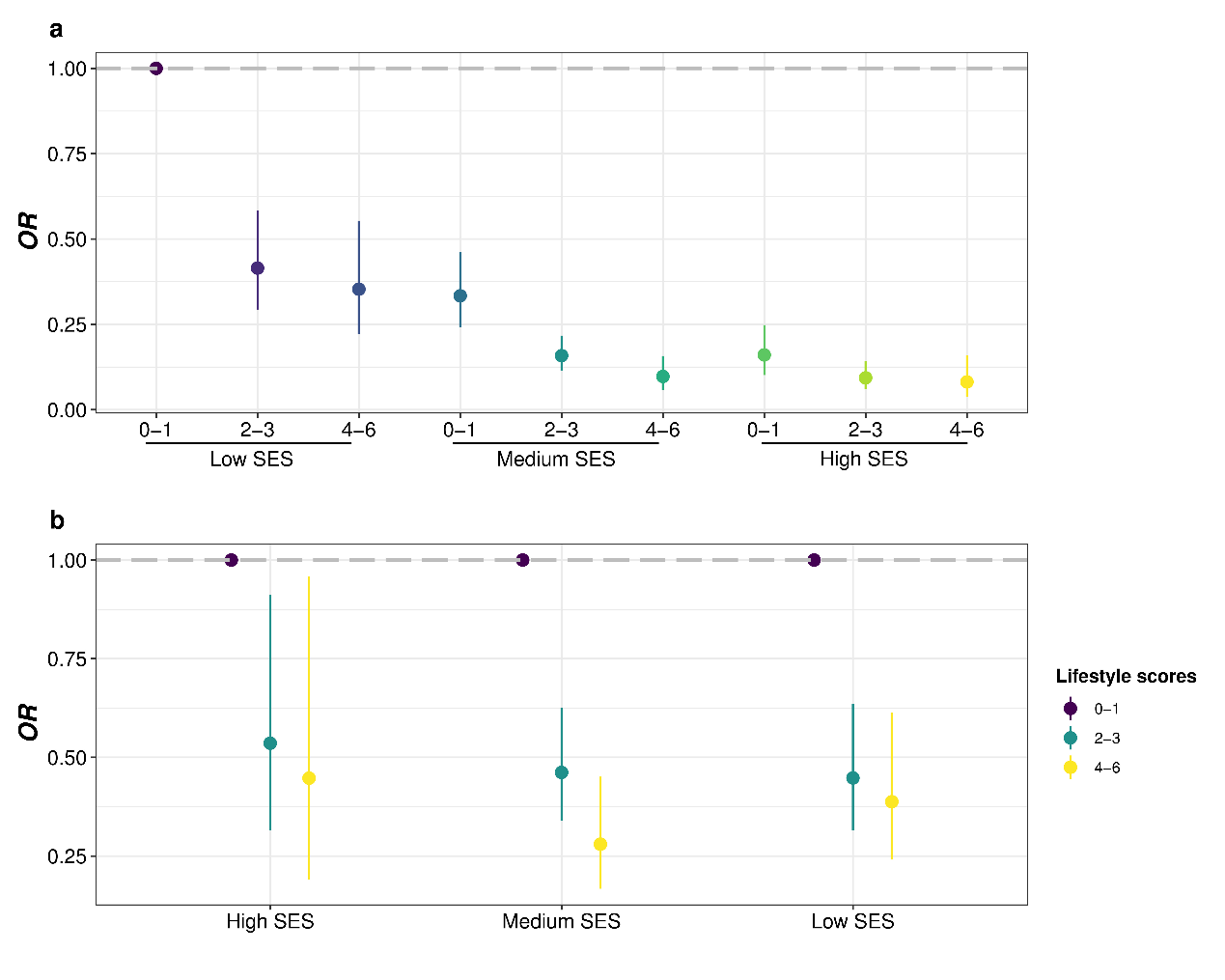


**Figure S16 Forest plot indicating lifestyle scores on blood or sexually transmitted infectious diseases in different SES subgroups from UK biobank.** The group with low SES and poor lifestyle scores (0–1) was selected as the overall control group **(a)**, or for each SES subgroup individually, that with poor lifestyle scores (0–1) was selected as the control group **(b)**. Odds ratios (*ORs*) were adjusted for age, sex, ethnic and assessment center. Dashed line represents no significant association. Abbreviations: SES: Socioeconomic status.


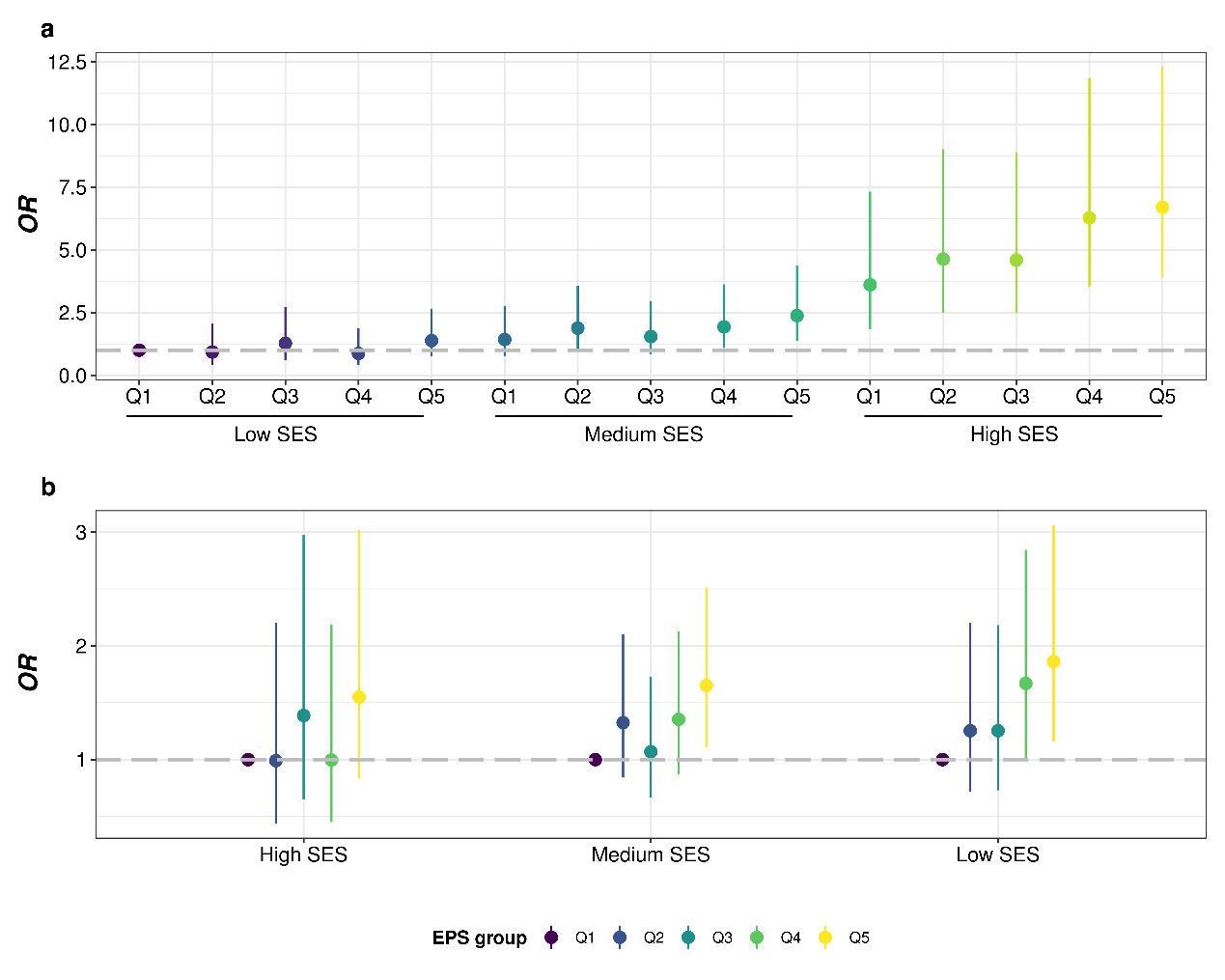


**Figure S17 Forest plot indicating environmental pollution score (EPS) groups on blood or sexually transmitted infectious diseases in different SES subgroups from UK biobank.** The group with high SES and low EPS (top fifth, Q1) was selected as the overall control group **(a)**, or for each SES subgroup individually, that with low EPS (Q1) was selected as the control group **(b)**. Odds ratios (*ORs*) were adjusted for age, sex, ethnic and assessment center. Dashed line represents no significant association. Abbreviations: SES: Socioeconomic status.


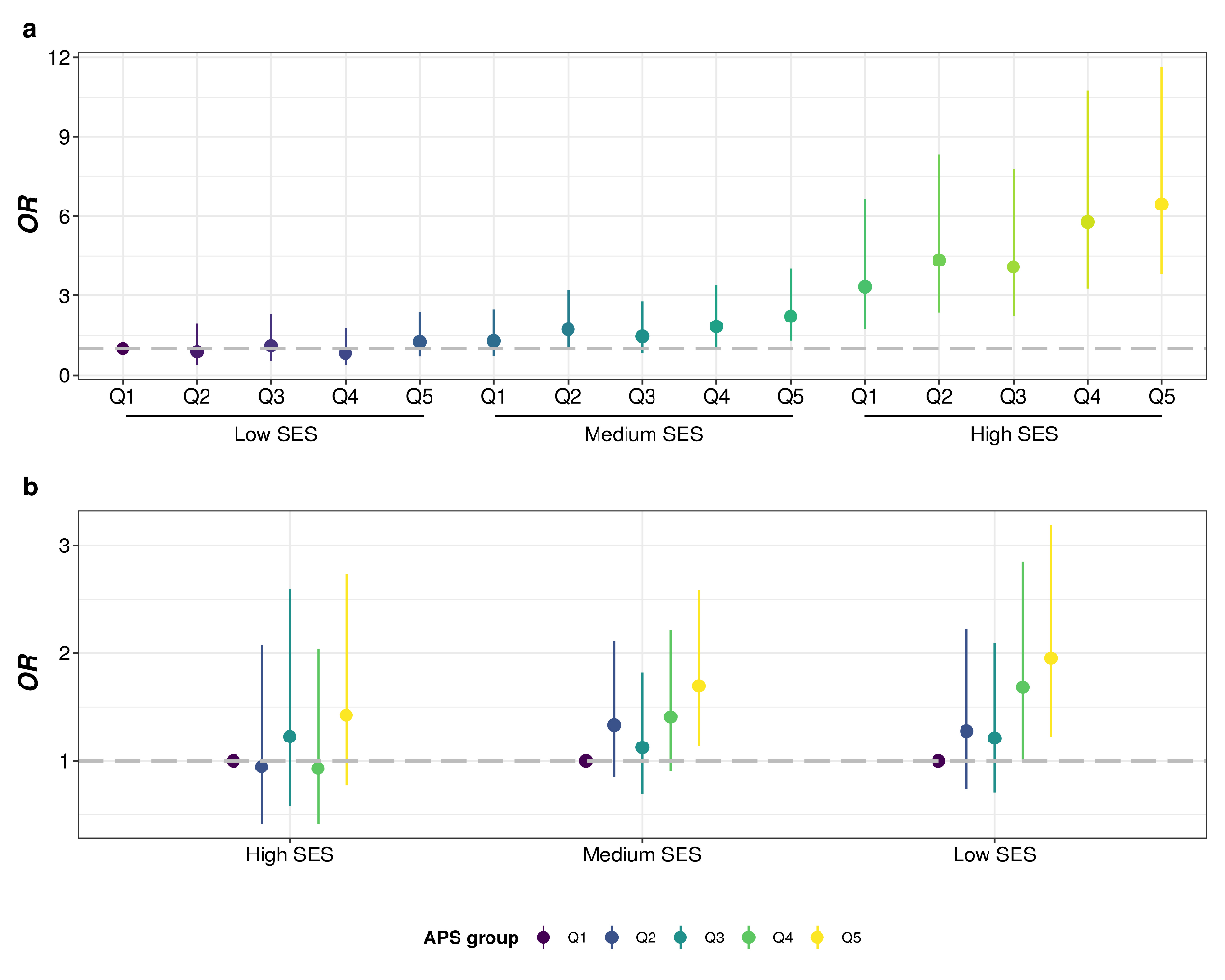


**Figure S18 Forest plot indicating air pollution score (APS) groups on blood or sexually transmitted infectious diseases in different SES subgroups from UK biobank.** The group with high SES and low EPS (top fifth, Q1) was selected as the overall control group **(a)**, or for each SES subgroup individually, that with low EPS (Q1) was selected as the control group **(b)**. Odds ratios (*ORs*) were adjusted for age, sex, ethnic and assessment center. Dashed line represents no significant association. Abbreviations: SES: Socioeconomic status.


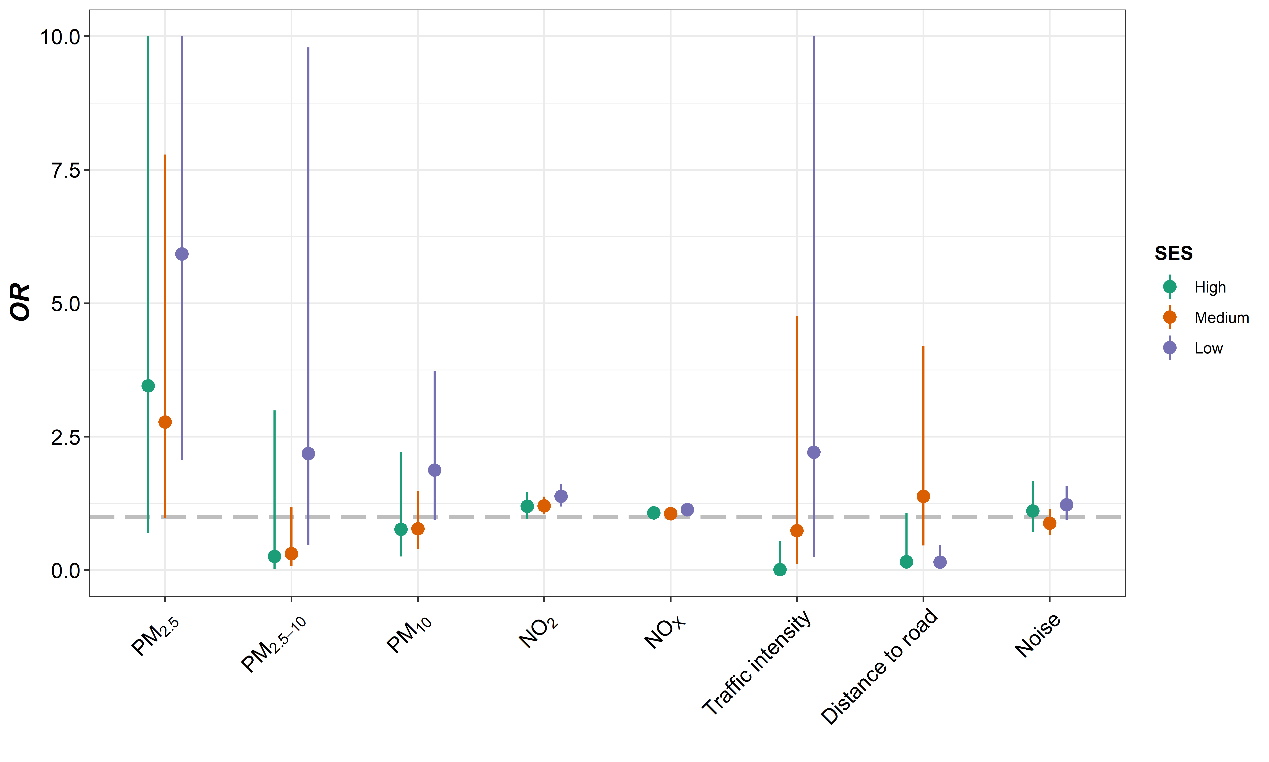


**Figure S19 Forest plot indicating individual environment pollution factors on blood or sexually transmitted infectious diseases in different SES subgroups from UK biobank.** Odds ratios (*ORs*) were estimated on per 10-unit increase, and were adjusted for age, sex, ethnic and assessment center. Confidence intervals (*CIs*) are truncated at 10. Dashed line represents no significant association. Abbreviations: PM_2.5_: Particulate matter ≤ 2.5 μm; PM_2.5–10_: Particulate matter 2.5–10 μm; PM_10_: Particulate matter ≤ 10 μm; NO_x_: Nitrogen oxides; NO_2_: Nitrogen dioxide.


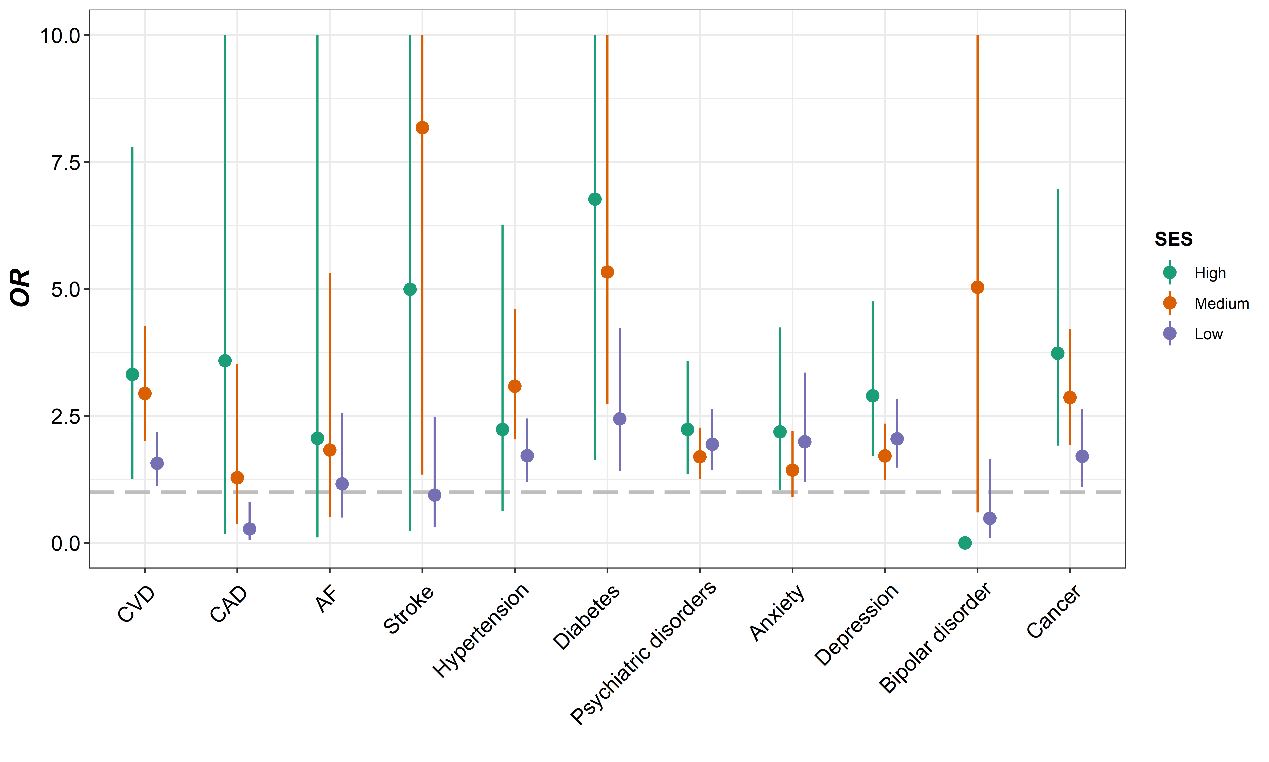


**Figure S20 Forest plot indicating chronic comorbidity factors on blood or sexually transmitted infectious diseases in different SES subgroups from UK biobank.** Odds ratios (*ORs*) were adjusted for age, sex, ethnic and assessment center. Confidence intervals (*CIs*) are truncated at 10. Dashed line represents no significant association. Abbreviations: CVD: Cardiovascular disease; CAD: Cardiovascular diseases; AF: Atrial fibrillation.


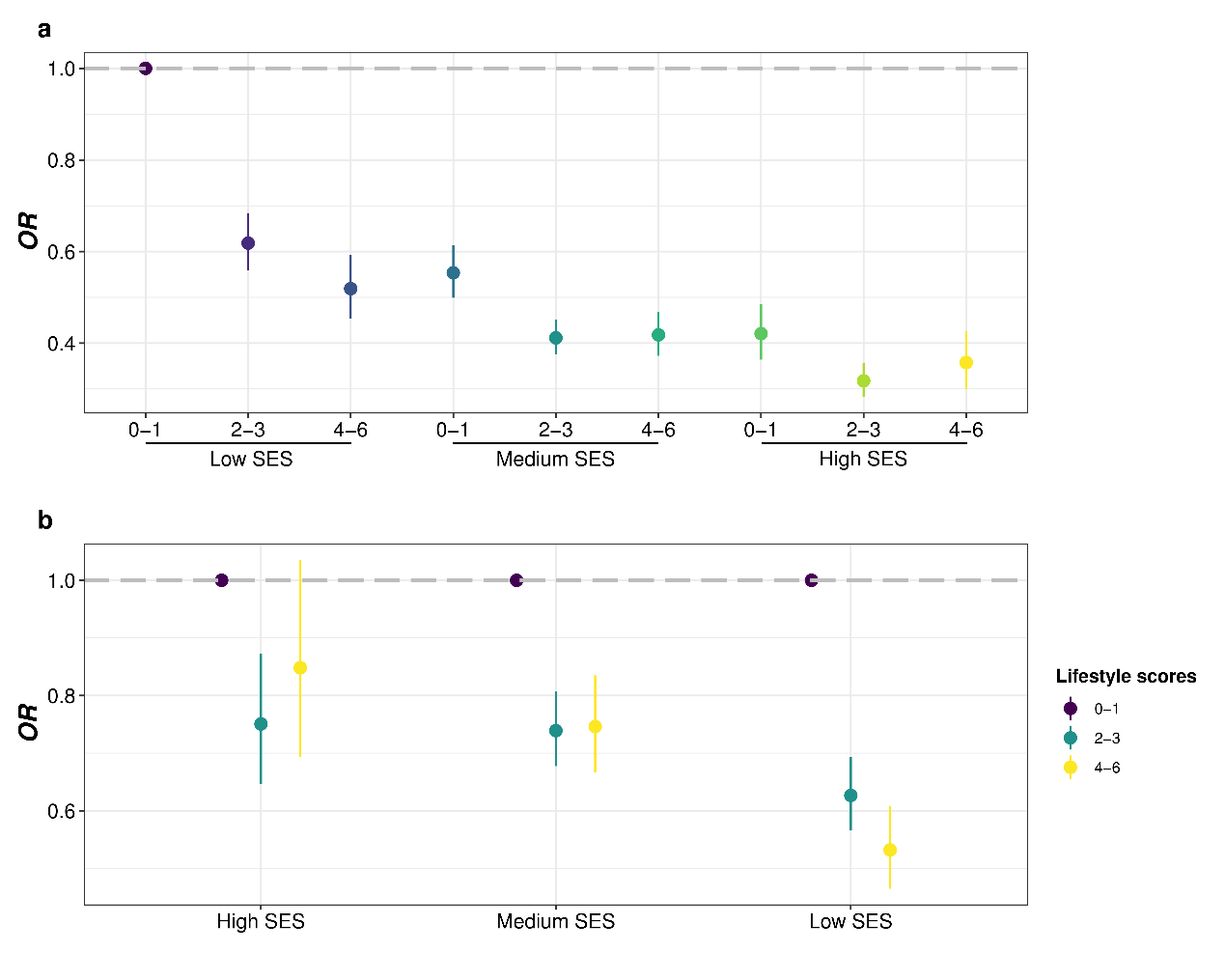


**Figure S21 Forest plot indicating lifestyle scores on infectious diseases in 2010 in different SES subgroups from UK biobank.** The group with low SES and poor lifestyle scores (0–1) was selected as the overall control group **(a)**, or for each SES subgroup individually, that with poor lifestyle scores (0–1) was selected as the control group **(b)**. Odds ratios (*ORs*) were adjusted for age, sex, ethnic and assessment center. Dashed line represents no significant association. Abbreviations: SES: Socioeconomic status.


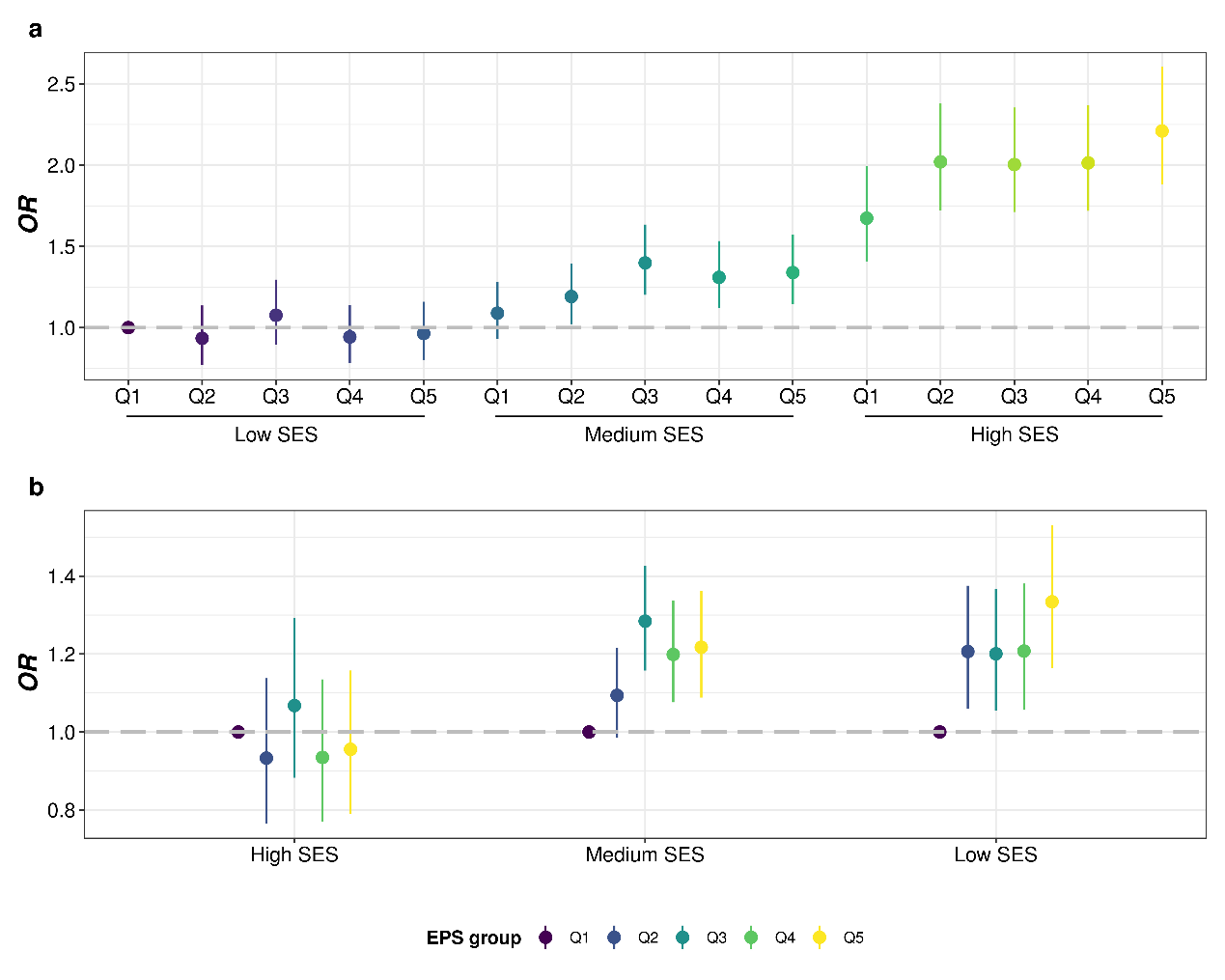


**Figure S22 Forest plot indicating environmental pollution score (EPS) groups on infectious diseases in 2010 in different SES subgroups from UK biobank.** The group with high SES and low EPS (top fifth, Q1) was selected as the overall control group **(a)**, or for each SES subgroup individually, that with low EPS (Q1) was selected as the control group **(b)**. Odds ratios (*ORs*) were adjusted for age, sex, ethnic and assessment center. Dashed line represents no significant association. Abbreviations: SES: Socioeconomic status.


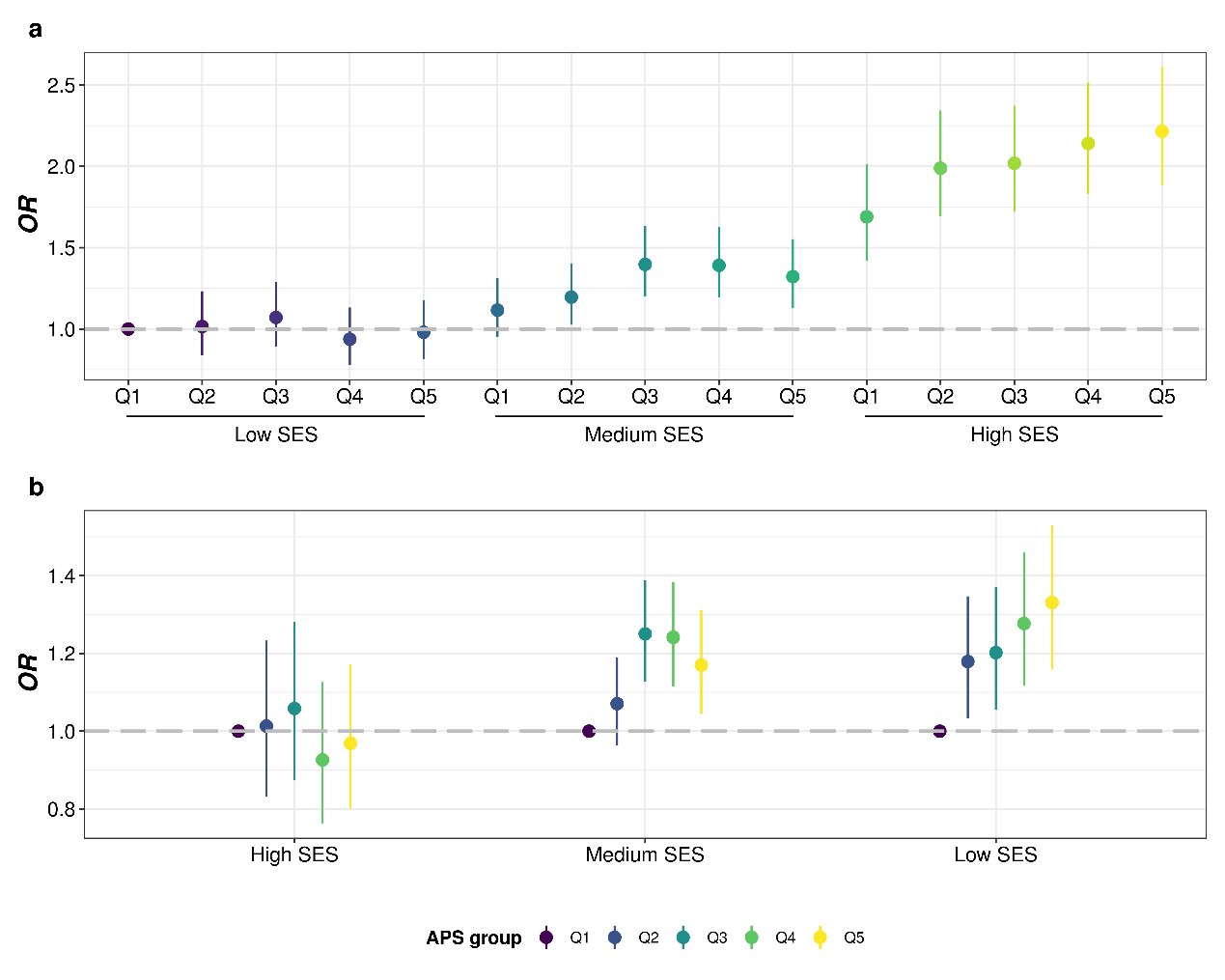


**Figure S23 Forest plot indicating air pollution score (APS) groups on infectious diseases in 2010 in different SES subgroups from UK biobank.** The group with high SES and low EPS (top fifth, Q1) was selected as the overall control group **(a)**, or for each SES subgroup individually, that with low EPS (Q1) was selected as the control group **(b)**. Odds ratios (*ORs*) were adjusted for age, sex, ethnic and assessment center. Dashed line represents no significant association. Abbreviations: SES: Socioeconomic status.


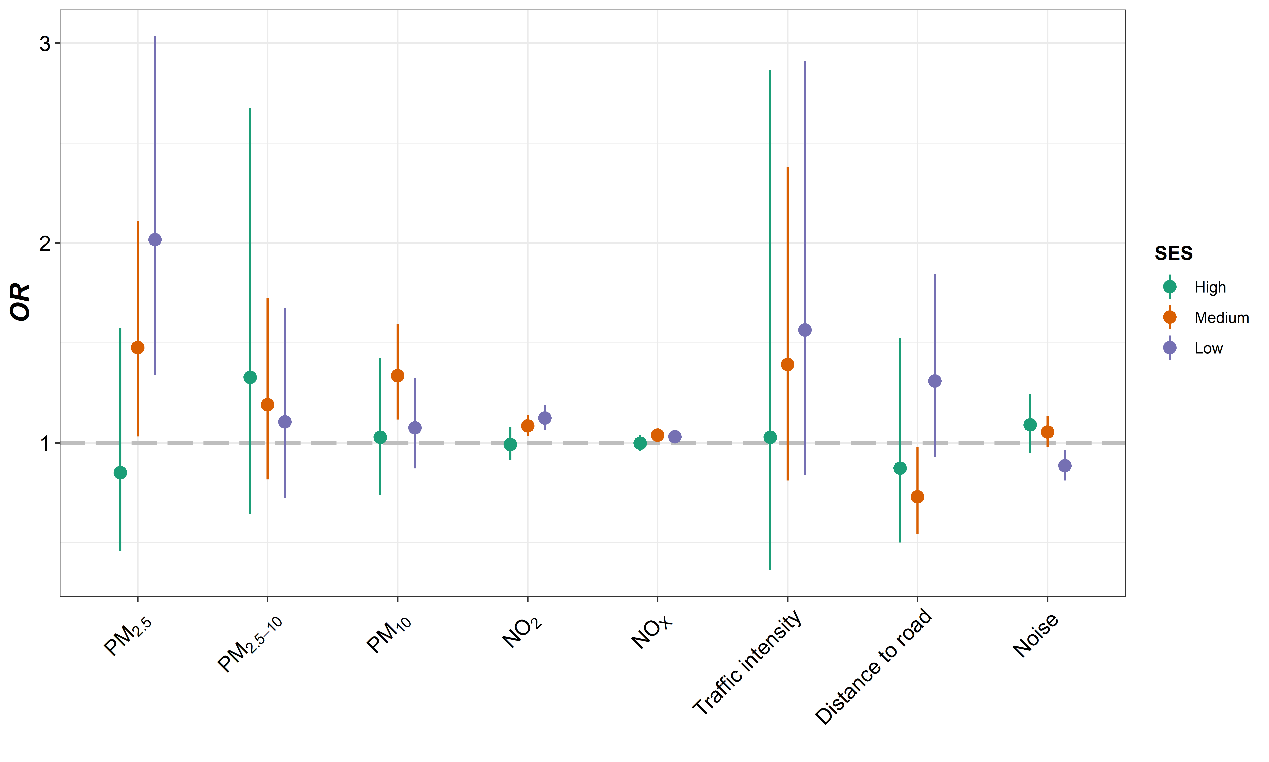


**Figure S24 Forest plot indicating individual environment pollution factors on infectious diseases in 2010 in different SES subgroups from UK biobank.** Odds ratios (*ORs*) were estimated on per 10-unit increase, and were adjusted for age, sex, ethnic and assessment center. Dashed line represents no significant association. Abbreviations: PM_2.5_: Particulate matter ≤ 2.5 μm; PM_2.5–10_: Particulate matter 2.5–10 μm; PM_10_: Particulate matter ≤ 10 μm; NO_x_: Nitrogen oxides; NO_2_: Nitrogen dioxide.


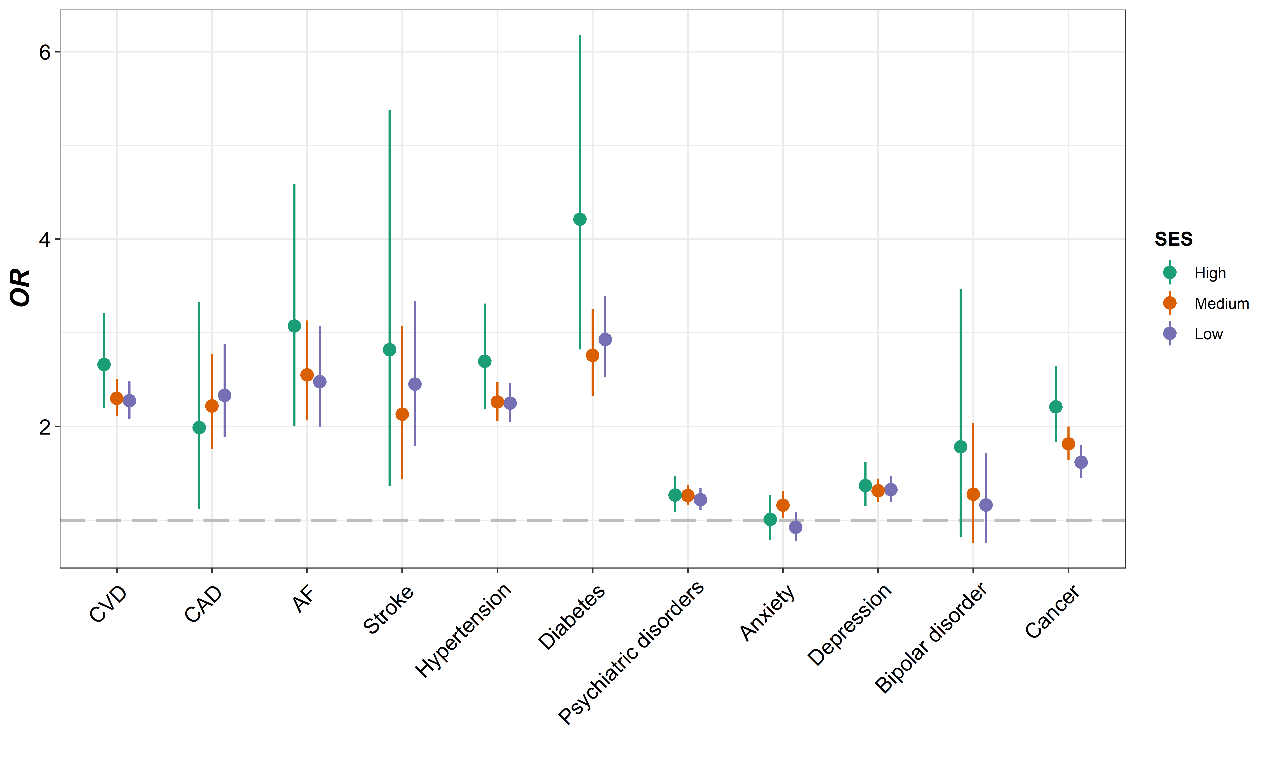


**Figure S25 Forest plot indicating chronic comorbidity factors on infectious diseases in 2010 in different SES subgroups from UK biobank.** Odds ratios (*ORs*) were adjusted for age, sex, ethnic and assessment center. Dashed line represents no significant association. Abbreviations: CVD: Cardiovascular disease; CAD: Cardiovascular diseases; AF: Atrial fibrillation.


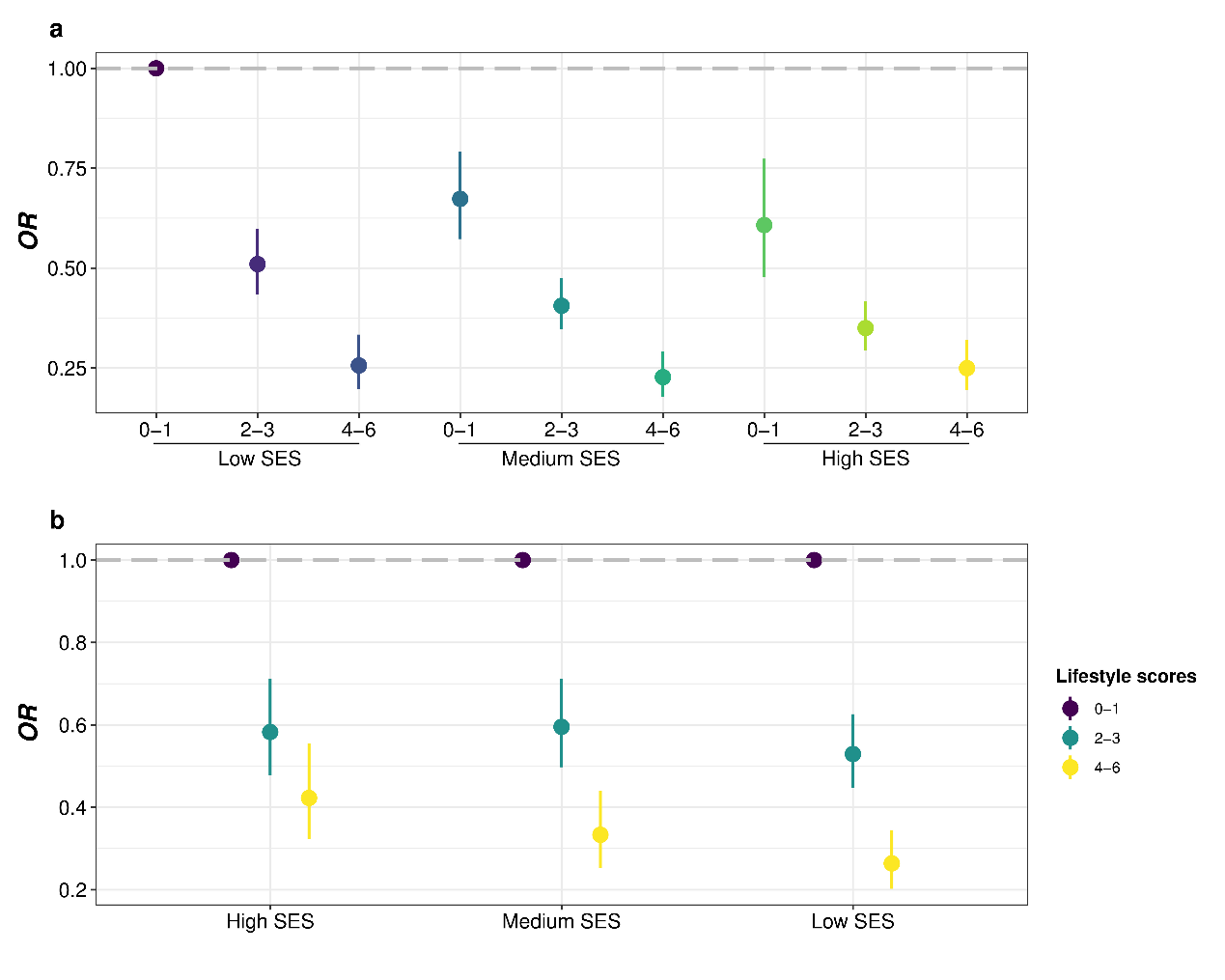


**Figure S26 Forest plot indicating lifestyle scores on infectious diseases in different SES subgroups from US NHANES.** The group with low SES and poor lifestyle scores (0–1) was selected as the overall control group **(a)**, or for each SES subgroup individually, that with poor lifestyle scores (0–1) was selected as the control group **(b)**. Odds ratios (*ORs*) were adjusted for age, sex, ethnic and survey cycle. Dashed line represents no significant association. Abbreviations: SES: Socioeconomic status.


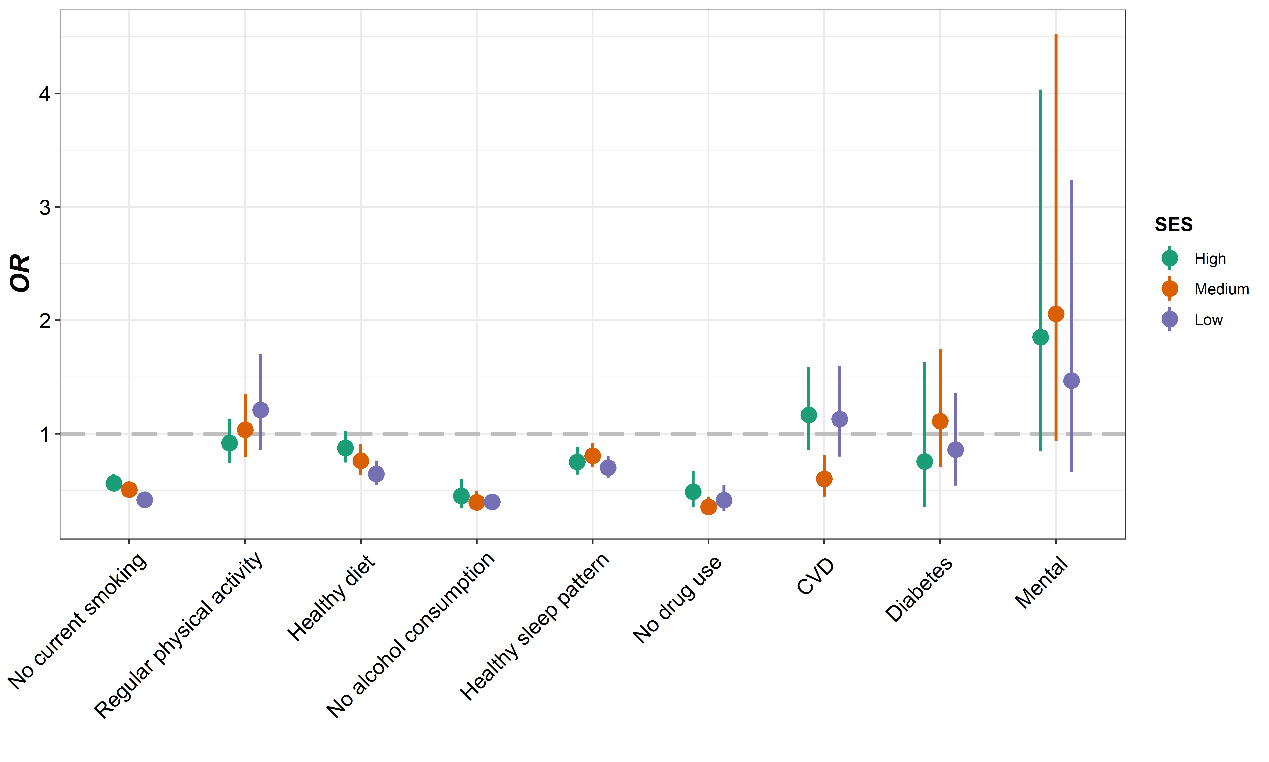


**Figure S27 Forest plot indicating individual lifestyle factors and chronic comorbidity factors on infectious diseases in different SES subgroups from US NHANES.** Odds ratios (*ORs*) were adjusted for age, sex, ethnic and survey cycle. Dashed line represents no significant association. Abbreviations: CVD: Cardiovascular disease.


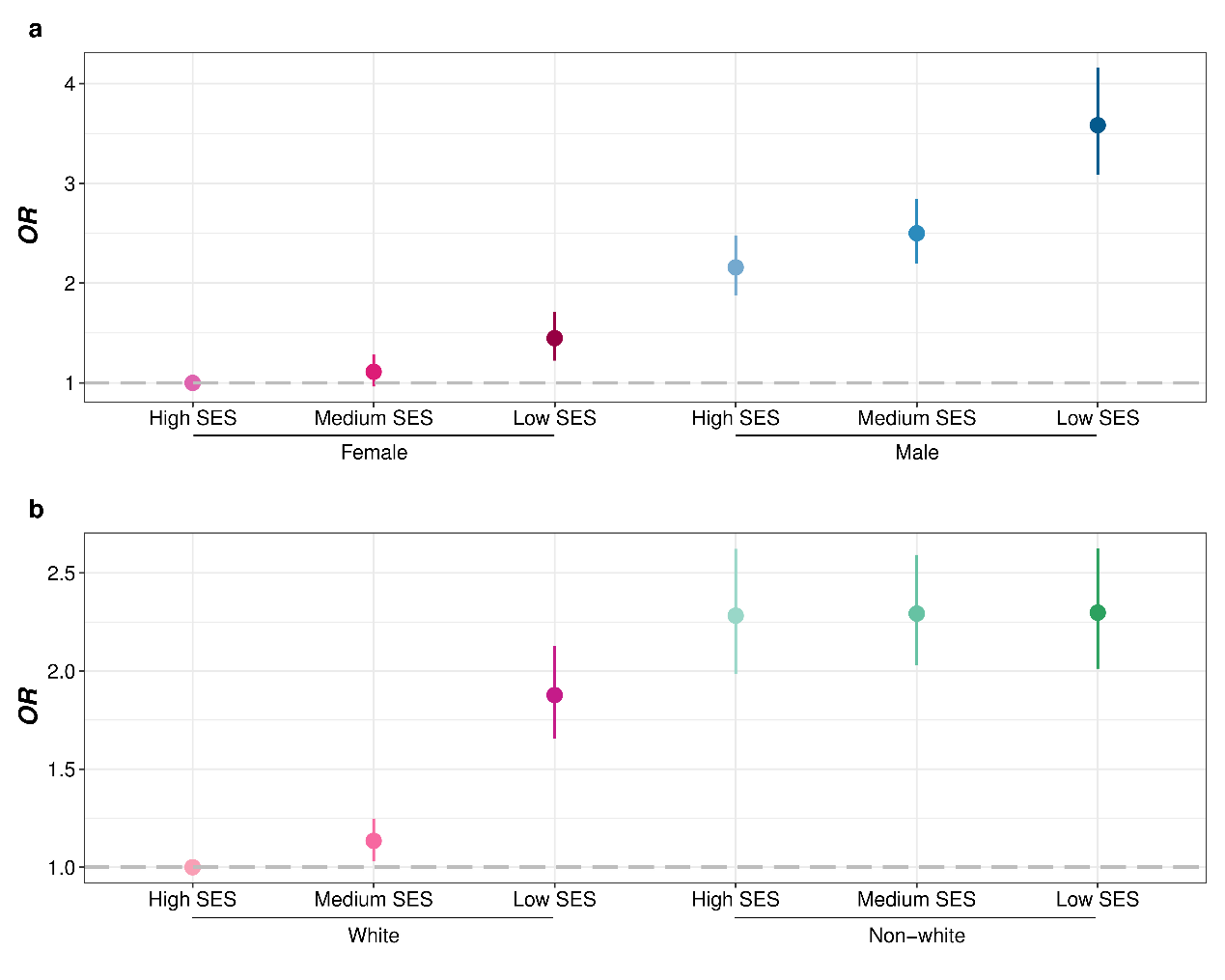


**Figure S28 Forest plot indicating SES on infectious diseases in different sex (a) and ethnic (b) subgroups from US NHANES.** Odds ratios (*ORs*) were adjusted for age, sex, ethnic and survey cycle. Dashed line represents no significant association. Abbreviations: SES: Socioeconomic status.
